# Competitive Binding of Viral Nuclear Localization Signal Peptide and Inhibitor Ligands to Importin-α Nuclear Transport Protein

**DOI:** 10.1021/acs.jcim.4c00626

**Published:** 2024-06-13

**Authors:** Bryan M. Delfing, Xavier E. Laracuente, William Jeffries, Xingyu Luo, Audrey Olson, Kenneth W. Foreman, Greg Petruncio, Kyung Hyeon Lee, Mikell Paige, Kylene Kehn-Hall, Christopher Lockhart, Dmitri K. Klimov

**Affiliations:** † School of Systems Biology, George Mason University, Manassas, Virginia 20110, United States; ‡ Department of Chemistry and Biochemistry, George Mason University, Fairfax, Virginia 22030, United States; § Center for Molecular Engineering, George Mason University, Manassas, Virginia 20110, United States; ∥ Department of Biomedical Sciences and Pathobiology, Virginia-Maryland College of Veterinary Medicine, Virginia Polytechnic Institute and State University, Blacksburg, Virginia 24061, United States; ⊥ Center for Emerging, Zoonotic, and Arthropod-Borne Pathogens, Virginia Polytechnic Institute and State University, Blacksburg, Virginia 24061, United States

## Abstract

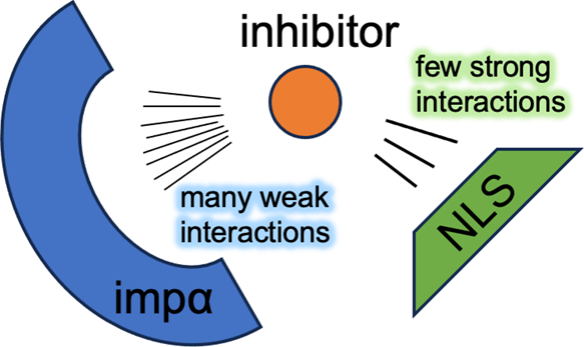

Venezuelan equine encephalitis virus (VEEV) is a highly virulent pathogen whose nuclear localization signal (NLS) sequence from capsid protein binds to the host importin-α transport protein and blocks nuclear import. We studied the molecular mechanisms by which two small ligands, termed I1 and I2, interfere with the binding of VEEV’s NLS peptide to importin-α protein. To this end, we performed all-atom replica exchange molecular dynamics simulations probing the competitive binding of the VEEV coreNLS peptide and I1 or I2 ligand to the importin-α major NLS binding site. As a reference, we used our previous simulations, which examined noncompetitive binding of the coreNLS peptide or the inhibitors to importin-α. We found that both inhibitors completely abrogate the native binding of the coreNLS peptide, forcing it to adopt a manifold of nonnative loosely bound poses within the importin-α major NLS binding site. Both inhibitors primarily destabilize the native coreNLS binding by masking its amino acids rather than competing with it for binding to importin-α. Because I2, in contrast to I1, binds off-site localizing on the edge of the major NLS binding site, it inhibits fewer coreNLS native binding interactions than I1. Structural analysis is supported by computations of the free energies of the coreNLS peptide binding to importin-α with or without competition from the inhibitors. Specifically, both inhibitors reduce the free energy gain from coreNLS binding, with I1 causing significantly larger loss than I2. To test our simulations, we performed AlphaScreen experiments measuring IC50 values for both inhibitors. Consistent with in silico results, the IC50 value for I1 was found to be lower than that for I2. We hypothesize that the inhibitory action of I1 and I2 ligands might be specific to the NLS from VEEV’s capsid protein.

## Introduction

Venezuelan equine encephalitis virus (VEEV) is a zoonotic alphavirus whose highly infectious nature threatens epidemic outbreaks.^1−3^ VEEV infections most commonly cause flu-like symptoms in humans; however, roughly 14% of cases result in severe neurological disorders, including encephalitis, with <1% being fatal. Despite this, there are currently no FDA-approved vaccines or antivirals available.^2^ VEEV’s capsid protein has previously been demonstrated to be a virulence factor that suppresses host immune response, blocking the nuclear pore complex (NPC) and disrupting nucleocytoplasmic trafficking.^2^ Specifically, VEEV’s capsid protein binds to the nuclear import protein, importin-α (impα), via its N-terminal nuclear localization signal (NLS) region.^4,5^ VEEV’s capsid is also able to bind to the nuclear export protein CRM1 via its nuclear export signal (NES). As a result, a tetrameric complex can form between impα, its partner protein importin-β, VEEV capsid, and CRM1 that blocks the NPC channel and interferes with nucleocytoplasmic traffic.^5^

**Figure 1:**
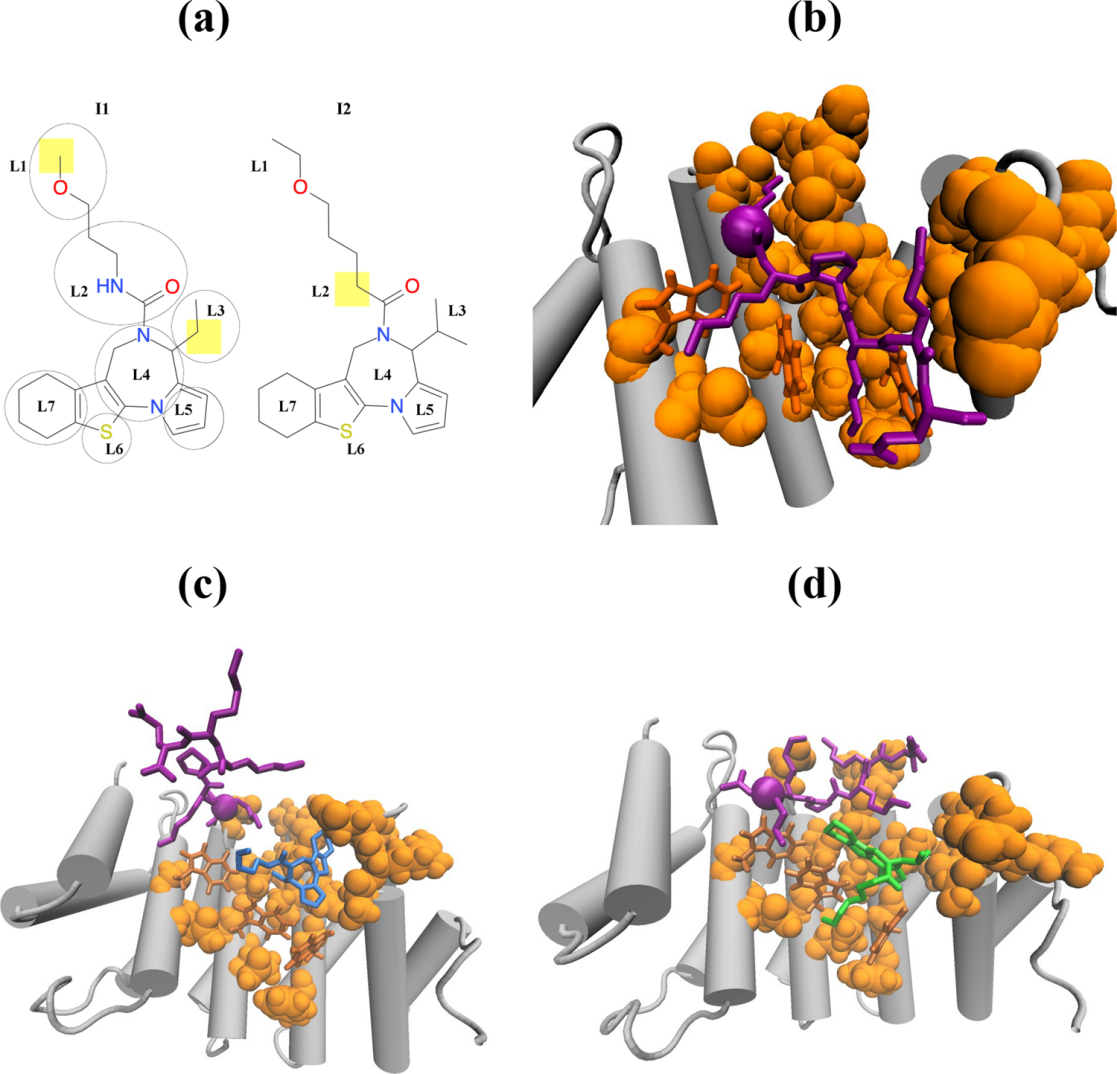
(a) Chemical structures of inhibitors I1 and I2 competing with the coreNLS peptide for binding to impα. Differences in their structures are highlighted in yellow. Ligand structural groups are marked. (b) Native pose of the coreNLS peptide in 3VE6 PDB structure. (c, d) Representative snapshots of the CB of the coreNLS peptide and I1 (c) or I2 (d) to impα major NLS binding site. The inhibitors I1 and I2 are colored blue and green, respectively. Impα amino acids constituting the coreNLS binding site are colored in orange. Three tryptophan amino acids are presented in licorice. The rest of the impα structure is in gray. The Lys6 Cα atom from the coreNLS peptide is represented by a sphere. Panels (b, d) demonstrate that although the I1 or I2 interference does not prevent the coreNLS binding to impα, it completely abrogates the peptide native pose.

Because this tetrameric complex plays a key role in VEEV’s pathogenesis, inhibiting the binding between VEEV capsid and either impα or CRM1 can be an effective approach to treating the virus infection.^5−7^ Following this line of reasoning, several small compounds from the CL6662 scaffold family have been examined as drug targets against VEEV.^8^ This scaffold was originally prepared as part of the Queensland Compound Library Open Scaffolds collection,^9^ with specific members, namely G281–1485 and its derivative I1 (Figure 1a), selected based on their inhibition of the VEEV-impα complex.^8^ Importantly, I1 showed specificity toward VEEV’s NLS compared to the simian virus SV40 large T-antigen NLS.^8^ Previously, we analyzed the binding of some of these compounds to impα in the absence of the viral NLS, identifying potential candidates for inhibition.^10^ We showed that these ligands, including I1 and I2 in Figure 1a, bind to the impα major NLS binding site diffusively without forming specific poses. We estimated from the experimental EC50 values that the inhibitor binding free energy Δ*G*_b_ is about −7 kcal/mol. For comparison, previous studies have argued that the typical free energy of binding of an NLS sequence, e.g., from SV40 virus, to impα is about −10 kcal/mol.^11^ A more recent report indicated that some NLS sequences, for example, from chloride intracellular channel proteins, may have markedly lower affinities of about −5 kcal/mol.^12^ Since VEEV NLS must compete with the host proteins for binding to impα, its Δ*G*_b_ should be in the same range. (For brevity, we refer to VEEV capsid NLS as VEEV NLS.) These free energy estimates suggest that the binding affinities of inhibitors might be sufficient to interfere with VEEV NLS binding. However, to directly test this hypothesis, one should simulate the inhibitors together with the viral NLS and analyze their competition for binding (Figure 1a). The VEEV NLS sequence A_5_KKPKKE_11_ (positions P1–P7) follows the monopartite NLS consensus sequence K-K/R-X-K/R, placing Lys, Lys, Pro, and Lys at positions P2–P5.^13^ The numbering of VEEV NLS is taken from the resolved X-ray structure, PDB entry 3VE6, depicting the complex formed between the 12-mer VEEV NLS sequence E_1_GPSAKKPKKEA_12_ and the mouse impα2 protein^14^ (Figure 1b). In this structure, Lys6 forms an electrostatic contact with Asp122 of impα, while Lys7 and Lys9 lie within the cages formed by Trp72, Trp114, and Trp161, forming π–cation interactions with their indole rings. Our recent molecular dynamics simulations of the NLS-impα complex showed that upon binding the 6-mer coreNLS sequence, K_6_KPKKE_11_, largely reproduces the native pose seen in 3VE6, while the shorter NLS sequences, the minNLS K_6_KPK_9_ or K_6_KPKK_10_, failed to reproduce the crystallographic native binding structure.^15^ We argued then that the coreNLS sequence is sufficient for native binding, but none of the amino acids flanking minNLS, including Lys10 and Glu11, is strictly necessary for the native pose. This conclusion followed from the observation that at least one other sequence KKPKIR (PDB code 4WV6) confers the same bound pose as the VEEV coreNLS. Our study has further indicated that the VEEV coreNLS sequence is unique among human and viral proteins, which interact with impα, making it a potential target for VEEV-specific inhibitors. However, the question remains how these binding interactions are affected by the inhibitors.

To investigate the inhibitory activities of the G281–1485 ligand family, we performed all-atom replica exchange molecular dynamics simulations with solute tempering (REST), probing the competitive binding (CB) of the VEEV coreNLS peptide and G281-1485 variants, I1 or I2, to impα the major NLS binding site (Figure 1). Our main result is that, although these ligands do not entirely block NLS binding to impα, they abrogate the native binding of the coreNLS peptide. This outcome is supported by a significant reduction in the free energy gain from the binding of coreNLS to impα. We found that inhibitors directly target these interactions, primarily by masking the coreNLS amino acids. The molecular mechanisms of inhibition are discussed.

## Model and Methods

### All-Atom Explicit Solvent Model

Two systems were simulated, each containing one inhibitor, denoted as I1 or I2, a truncated, 211 residue importin-α (impα) protein, and the coreNLS peptide K_6_KPKKE_11_ from the NLS region of VEEV capsid protein^5^ (Figure 1). The protein structure and the “native” bound peptide pose were taken from PDB entry 3VE6. The mouse structure of impα was used to facilitate binding analysis, as it is the only structure containing VEEV’s NLS bound to impα. The RCSB PDB pairwise structural alignment tool^16^ indicates that 3VE6 impα fold is nearly identical to its human counterpart, 3FEY,^17^ with 98% sequence similarity and a fitted RMSD of 0.69 Å between the two. The protein was truncated at position 211 to include the major NLS binding site, while reducing the computational costs. The protein and peptide were capped with neutral acetylated and amidated groups. The inhibitors I1 and I2 were derivatives of G281-1485^8^ and have been taken from our previous study.^10^ I1 designated as G281-1564 has been studied experimentally^8^ and is more polar than I2 (Figure 1a). Both the inhibitor and peptide centers of mass were confined, using soft harmonic potential, to a sphere with a radius of 18 Å. To include the major NLS binding site and adjacent amino acids in the sphere, its center was offset from the center of the major NLS binding site. As a result, about 67% of the sphere volume was protein-free, in which the coreNLS peptide and an inhibitor resided. Soft harmonic restraints^18^ were applied to the protein Cα beyond the sphere to preserve the native impα fold in the absence of its autoinhibitory domain blocking importin β binding. These restraints kept the protein structure similar to that in 3VE6, while still allowing fluctuations. Thus, these two simulation systems were designed to examine the CB of the coreNLS peptide and inhibitors to impα.

The all-atom CHARMM36m force field^19^ was used to model both the protein and peptide, while the CHARMM General Force Field (CGenFF)^20,21^ was used for the two inhibitors. The CHARMM-modified TIP3P model^22,23^ was used for water. The systems were solvated with a water box of roughly 58 x 58 x 77 Å. A NaCl salt in a concentration of 150 mM was added while neutralizing the overall charge of the system, for a total of 22 chloride ions and 24 sodium ions. Aside from the ligands, the only difference between the two systems was a slight variation in the number of water molecules, with 7686 waters in the I1 system and 7671 in the I2 system. This brought the total atom counts to 26,491 and 26,453, respectively.

### Replica Exchange Simulations

Isobaric–isothermal replica exchange with solute tempering (REST) molecular dynamics^24^ was used to sample interactions between the peptide, ligands, and protein. A short overview of REST formalism is provided below with further documentation available elsewhere.^24,25^ A total of *R* = 10 REST conditions were considered with the temperatures ranging from *T*_0_ = 310 K to *T*_*R*–1_ = 510 K. Exchanges between replicas *r* and *r* + 1, simulated at temperatures *m* and *m* + 1, occur at a rate of ω = min[1, e^–Δ^], where Δ = β_*m*_(*H*_*m*_(*X*_*r*+1_) – *H*_*m*_(*X*_*r*_)) + β_*m*+1_(*H*_*m*+1_(*X*_*r*_) – *H*_*m*+1_(*X*_*r*+1_)), β = (*R*_c_*T*)^−1^, *H* is the enthalpy, *X* refers to the system coordinates, and *R*_c_ is the gas constant. The solvent–solvent and solute–solvent interactions at a temperature *T*_m_ were scaled by the *T*_m_/*T*_0_ and (*T*_m_/*T*_0_)^1/2^ factors, respectively. This scaling serves to exclude solvent–solvent interaction energies from ω and reduce the number of replicas while maintaining a wide temperature range and reasonable exchange rates. The peptide and ligands were treated as “hot” solute, solute–solvent interactions were partially tempered, and the water, protein, and ions were left as untempered, effectively “cold” solvent. Replica exchange attempts occurred at 2 ps intervals, with success rates of about 0.34 for both systems.

NAMD^26^ was used to perform REST simulations with periodic boundary conditions and an integration step of 1 fs. Hydrogen-based covalent bonds were constrained by using the SHAKE algorithm. Ewald summation was used for computing electrostatic interactions, and van der Waals interactions were smoothly switched off from 8 to 12 Å. The temperature was controlled using underdamped Langevin dynamics with the damping coefficient γ = 5 ps^–1^. The pressure was set at 1 atm using the Nosé–Hoover Langevin piston method with a piston period of 200 fs and a decay of 100 fs. The *x*, *y*, and *z* dimensions of a simulation system were coupled.

Four REST trajectories were produced for each inhibitor system. The starting structures for the REST trajectories were prepared as follows. First, poses of the peptide and inhibitor were selected from our previous simulations probing their noncompetitive binding (NCB) to impα^10,15^ and inserted into the sphere. After energy minimization and heating to 310 K, the systems were simulated for 25 ns at 700 K by employing REST energy scaling. The purpose of these high temperature simulations was to randomize the peptides and inhibitors in the sphere. The structures were then selected from these simulations at the interval of 1 ns between 16 and 25 ns, with each undergoing further 1 ns of equilibration at the respective REST temperature. From these final equilibration steps, the initial structures were selected for REST simulations. As a result, each REST trajectory has unique initial structures at each temperature.

For the competitive simulations with I1 inhibitor, each replica in a trajectory was simulated for 200 ns, for a total of 0.8 μs per temperature and 8.0 μs of sampling across all temperatures and trajectories. Due to a longer equilibration period, the competitive simulations with I2 were performed for 400 ns per replica in each trajectory, totaling 1.6 μs per temperature and 16.0 μs of sampling in all. For I1 and I2 simulations, we excluded as nonequilibrated the first 140 and 260 ns of sampling, respectively, at each temperature and trajectory. Thus, 240 and 560 ns of equilibrated 310 K sampling were retained for analysis of the respective systems. Analysis of the REST performance and convergence can be found in Supporting Information 1 (SI1).

### Computation of Structural Probes

Peptide or inhibitor binding to impα was probed by computing the contacts between residues or ligand groups (Figure 1a), defined by a minimum distance of 4.5 Å between the heavy atoms. Two molecules are assumed bound if at least one contact is formed between them. The coreNLS native binding pose and site were defined by using the contacts made in the 3VE6 structure (Figure 1b). Based on this definition, we computed the fraction of native contacts *P*_n_(*j*) present in the 3VE6 structure between a given peptide residue *j* and impα that are also retained in REST simulations. Thus, *P*_n_(*j*) = 1.0 implies that *j* forms all of its native interactions. Similarly, we computed the fraction of nonnative contacts *P*_nn_(*j*) among all contacts formed by *j* in our REST sampling. The probabilities for impα amino acid *i* to bind either the peptide or the ligand, respectively, were computed as *P*_b,p_(*i*) or *P*_b,i_(*i*). The impα amino acids with the top ten such probabilities, either for the peptide or ligand, were selected for analysis. VMD was used to measure hydrogen bonding,^27^ applying a donor (D)–acceptor (A) cutoff distance of 3.5 Å and a minimum DHA angle of 135°. All data are computed by averaging across equilibrated data at *T*_0_ = 310 K. Standard errors were computed using each REST trajectory as an individual sample.

### Conformational Ensembles and Clustering

Density-based conformational clustering of peptide poses was performed using the method of Daura et al.^28^ Prior to peptide clustering, the sampled impα structures were aligned based on minimal root-mean-squared deviations (RMSD) of impα side chains from the coreNLS native binding site. The distributions of RMSDs between peptide poses were then computed without aligning the peptides themselves. Therefore, this procedure captured the distributions of coreNLS binding poses quantified by the RMSD values. A total of 10,000 poses were sampled for each system, selected periodically from the equilibrated REST data. Besides computing RMSD distributions, we perform peptide clustering using the RMSD cutoff *R*_0_ = 2 Å^29^ and retaining clusters with a minimum of 1% of all structures.

### Computation of Binding Free Energies

To compute the free energy of the coreNLS peptide binding to impα Δ*G*_b_, we used the MM-GBSA approach.^30^ Then, the free energy of a solute is
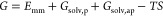
1where *E*_mm_ is the molecular mechanical energy of solute, *G*_*solv,p*_ is the polar contribution to the solute solvation free energy computed using the Generalized Born implicit solvent model,^31^*G*_solv,ap_ is the apolar contribution to the solvation free energy, and *TS* is the solute conformational entropy. We set the dielectric constant to 78.5, estimated the solvent accessible surface area with a probe of 1.4 Å radius, and set the nonpolar surface tension coefficient to γ = 0.005 kcal/mol/Å^2^.^32^ The solute entropy *S* was given by Gibbs expression *S* = −*R*_c_ ∑_*E*_mm__*P*(*E*_mm_) ln *P*(*E*_mm_), where *P*(*E*_mm_) is the probability distribution of *E*_mm_. The bin size for *E*_mm_ was 1 kcal/mol. The free energy of the coreNLS binding to impα is
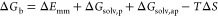
2where Δ*E*_mm_, Δ*G*_solv,p_, Δ*G*_solv,ap_, and *T*Δ*S* are the changes in the terms from eq 1 upon binding. To evaluate the impact of inhibitor on the coreNLS binding to impα, we defined Δ Δ*G*_b_(*x*) = Δ*G*_b_(CB;*x*) – Δ*G*_b_(NCB), where *x* = I1 or I2 and CB or NCB stand for CB and NCB of the peptide to impα. The contributions of translational, rotational, or vibrational entropies of impα and coreNLS to Δ*G*_b_ are not accounted in eq 2. However, we can reasonably assume that their changes upon binding are not affected by the inhibitors. Consequently, we excluded these terms from the analysis.

We first computed the binding free energy Δ*G*_b_(NCB) from NCB simulations^15^ following a straightforward application of MM-GBSA methodology. Evaluation of Δ*G*_b_(CB;*x*) for the binding of the coreNLS to impα in the presence of the inhibitor *x* is more difficult because it requires delineation of impα, coreNLS, and inhibitor contributions. We treat *x* as an environmental factor, which upon binding to impα or the coreNLS peptide excludes a part of their surface from the interactions with water substituting them with the interactions of *x* with impα or the peptide. Thus, the molecular mechanical and solvation energies of *x* are omitted from Δ*G*_b_(CB;*x*). Further, to compute Δ*G*_b_(CB;*x*), we need to establish if *x* binds to impα or the coreNLS peptide before the formation of the impα + NLS complex. To distinguish between the two possibilities, we used AutoDock Vina^33,34^ and compared the binding affinities of *x* to impα and the coreNLS. The former affinities have been computed as −6.0 ± 0.1 kcal/mol for I1 and −6.2 ± 0.0 kcal/mol for I2. To compute the affinity of *x* to the coreNLS peptide, we performed separate REST simulations of the peptide in water and clustered its structures at 310 K. The centroids of the 12 largest clusters, which together represent at least 50% of the conformational ensemble, were used as targets for *x* binding. The AutoDock Vina scores were −4.0 ± 0.0 kcal/mol for I1 and −3.9 ± 0.1 kcal/mol for I2. Qualitatively similar results follow if we compute the average energies of interaction between the inhibitors and the coreNLS or impα in the CB simulations. Thus, because the affinity of the inhibitors *x* to impα is stronger than to the coreNLS, we assumed that *x* binds to impα prior to forming the impα + NLS complex. This assumption was used in computing Δ*G*_b_(CB;*x*).

## Results and Discussion

### CB of the coreNLS Peptide and Inhibitor I1 to Impα

The coreNLS peptide KKPKKE spans the NLS positions P2–P7.^15^ In the PDB structure 3VE6 this peptide, while being a fragment of a 12-mer VEEV NLS sequence, forms a native pose tightly bound to importin-α (impα) (Figure 1b). In all, the coreNLS peptide forms 32 native contacts with impα amino acids (see the Model and Methods section), of which those made by Lys6 (P2), Lys7 (P3), Lys9 (P5), and Lys10 (P6) are the most important. In particular, Lys6 establishes a salt bridge with impα Asp122, while Lys7 contacts the side chains of Trp114 and Trp161 forming a π–cation interaction with the latter. Lys9 resides in the cage formed by Trp72 and Trp114 and forms π-cation contacts with both. Finally, Lys10 acquires a salt bridge with Glu37.

**Figure 2:**
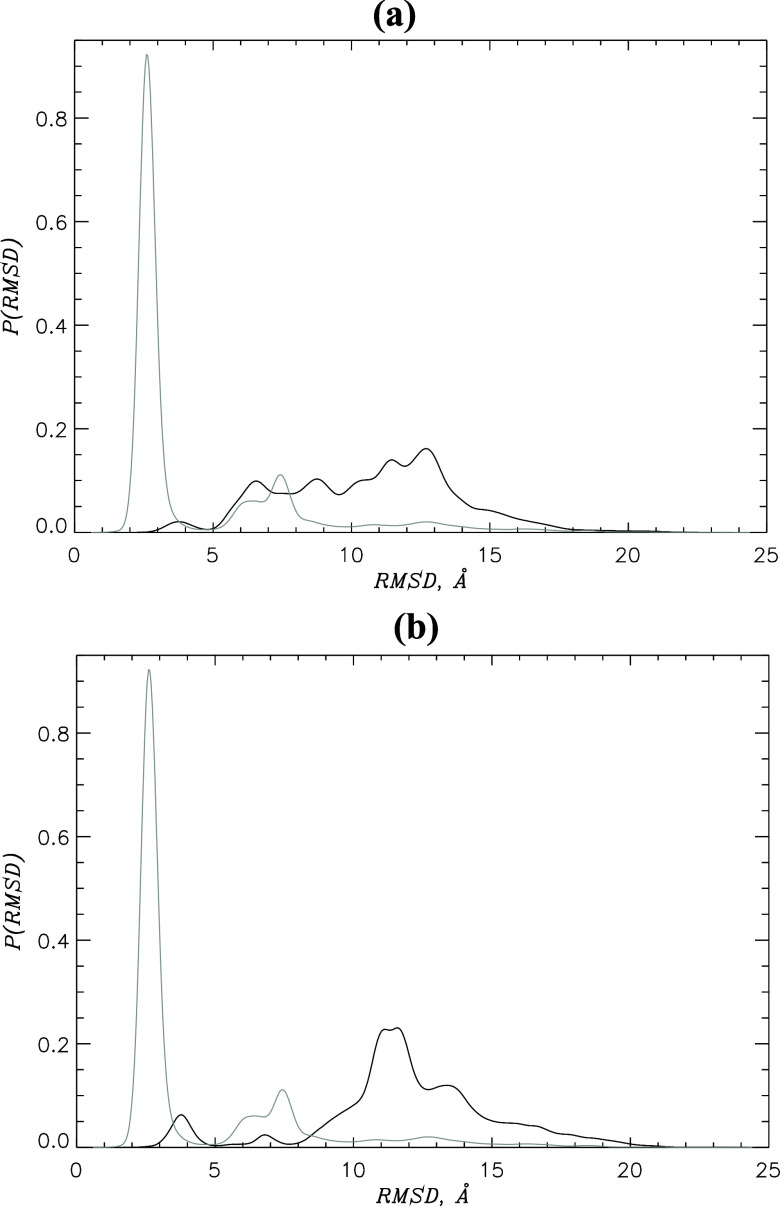
Probability distributions *P*(RMSD) of RMSD values computed between the native pose observed in 3VE6 structure and coreNLS binding poses at 310 K. Panels (a) and (b) refer to the coreNLS peptide competing with I1 or I2 inhibitors. The data in black and gray correspond to CB and NCB,^15^ respectively. Approximately unimodal NCB distribution peaking at ∼2.5 Å indicative of native binding contrasts sharply with the broad CB *P*(RMSD) distributions representing disordered peptide binding.

Our first objective was to study the impact of inhibitor I1 on the binding of the coreNLS peptide to impα. To this end, we performed REST simulations to probe their CB to impα. Our previous REST simulations, in which we examined an NCB of the same peptide to impα, served as a reference. The NCB simulations revealed that the coreNLS adopts nearly native pose at 310 K.^15^ The new, CB simulations show that at 310 K the coreNLS peptide still binds to impα major NLS binding site with the probability *P*_b_ ≃ 0.99 ± 0.00, which is similar to the NCB value (0.97 ± 0.02^15^). Furthermore, in the CB simulations, the probabilities of I1 binding to the coreNLS and impα are 0.80 ± 0.01 and 0.97 ± 0.00 implying that the inhibitor should influence the peptide–impα interactions. Indeed, although the inhibitor does not prevent peptide binding, its impact is nonetheless striking. Figure 2a compares the probability distributions *P*(RMSD) of RMSD values computed between the native pose and in silico coreNLS poses in CB and NCB^15^ simulations. NCB features an almost unimodal *P*(RMSD) distribution, in which the dominant peak represents the native cluster with the average native RMSD of 2.5 ± 0.1 Å gathering the fraction of 0.67 ± 0.01 of all peptide poses.^15^ The average RMSD computed for all peptide poses is <RMSD> = 4.6 ± 0.4 Å. In CB, there is a broad, multipeak *P*(RMSD) distribution extending up to RMSD > 15 Å and implicating a heterogeneous bound ensemble. The average RMSD for all peptide poses is increased to <RMSD> = 10.7 ± 0.5 Å that is a 2.3-fold higher than in the NCB. Critically, the cluster collecting native peptide poses with the average RMSD of 3.8 Å is virtually obliterated as its population fraction decreases to 0.02.

**Figure 3:**
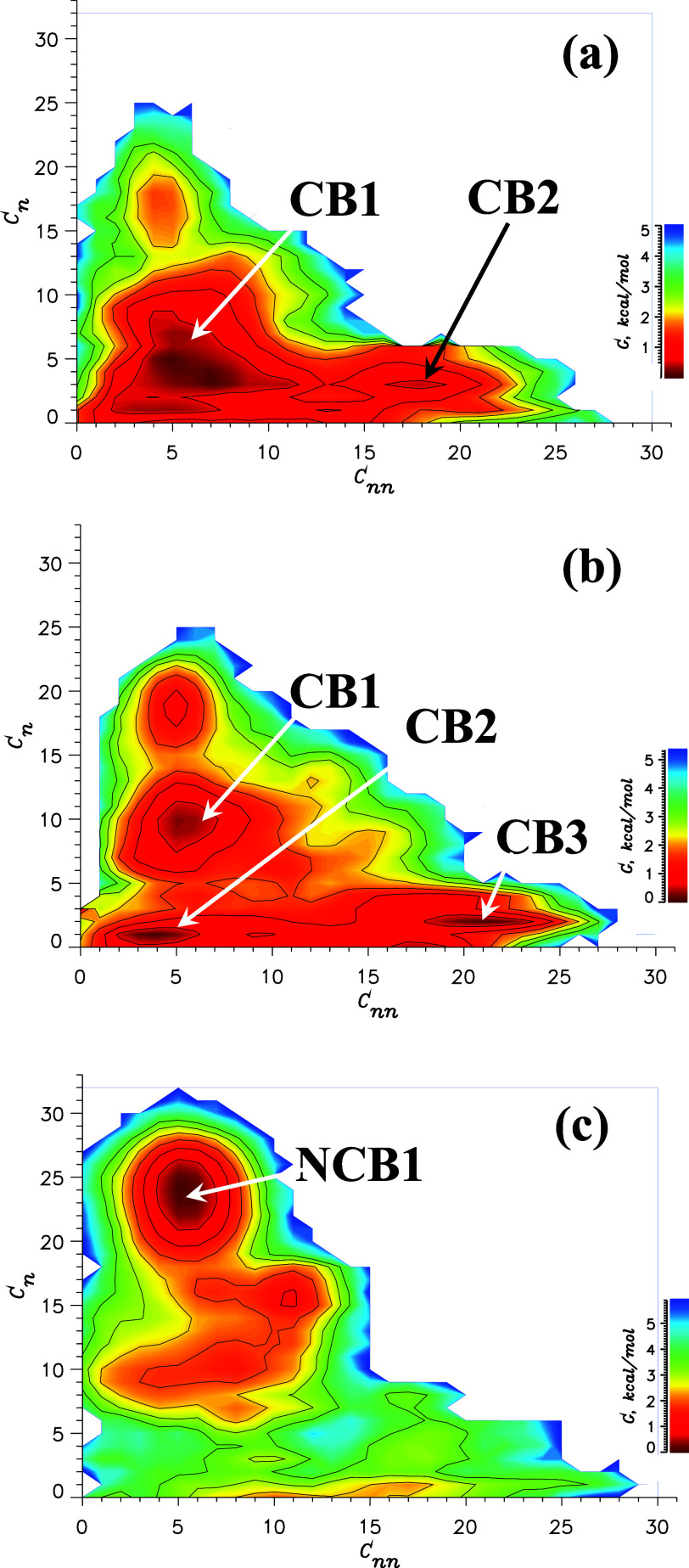
Two-dimensional free energy landscapes *G*(*C*_n_, *C*_nn_) are shown as functions of the numbers of native *C*_n_ and nonnative *C*_nn_ contacts for the CB of the coreNLS peptide and inhibitors I1 (a) or I2 (b). For reference, panel (c) presents *G*(*C*_n_, *C*_nn_) for the NCB.^15^ The contour lines have increments of 0.5 kcal/mol. The low free energy states are marked. The figure shows a dramatic loss of native interactions in the low free energy states in CB.

To further compare the CB and NCB of the coreNLS peptide, we computed the binding free energy *G*(*C*_n_, *C*_nn_) = −*RT* ln *P*(*C*_n_, *C*_nn_), where *P*(*C*_n_, *C*_nn_) is the probability for a bound peptide to form the numbers of native and nonnative contacts *C*_n_ and *C*_nn_, respectively. Figure 3 comparing *G*(*C*_n_, *C*_nn_) for CB and NCB reveals that the inhibitor dramatically shifts the peptide low free energy states. Consistent with the RMSD analysis, NCB features a single, native-like state NCB1 with the large number of native contacts *C*_n_ ∼ 24 and few nonnative interactions *C*_nn_ ∼ 5.^15^ The population of this state is 0.67. Although in CB there is also a single, dominant low free energy state CB1 incorporating 64% of coreNLS poses, it is wide and has almost no native content. Indeed, the number of native contacts in CB1 is reduced 6-fold to *C*_n_ ∼ 4, while the contribution of nonnative interactions remains low. The minor state CB2 with the population of 0.08 has an elevated number of nonnative binding interactions. The shift in the CB free energy basins reflects a dramatic loss of binding, including native, interactions formed by the coreNLS peptide with impα upon the competition from I1.

It is instructive to examine the specific changes in the peptide and I1 binding interactions. To this end, we computed the probabilities *P*_b,p_(*i*) of forming contacts between impα amino acids *i* and the coreNLS peptide. Table 1 lists the ten impα amino acids with the largest *P*_b,p_(*i*) observed in CB and, for reference, in NCB.^15^ This table allows us to identify the peptide binding sites on impα and the nature of the binding interactions. It follows from Table 1 that seven out of ten impα amino acids are native; i.e., they also interact with the coreNLS peptide in the PDB structure. For comparison, all top ten impα amino acids in NCB are native. Furthermore, six amino acids present in NCB are retained in CB. Thus, I1 does not block the coreNLS from binding to the major NLS impα site, but, as alluded by Figures 2a and 3a, it disorganizes peptide binding. This conclusion is supported by the computation of the correlation coefficient *r* between the *P*_b,p_(*i*) vectors computed for CB and NCB. (For this analysis, we selected impα amino acids *i* with *P*_b,p_(*i*) > 0.1 in either of the two binding simulations.) We found *r* = 0.27 indicating that the I1 inhibitor severely changes the coreNLS binding to impα. The computations of the probabilities *P*_b,i_(*i*) probing I1 binding (see Table S1 in SI1) revealed a different outcome. Indeed, nine out of the top ten impα amino acids are native, and nine observed in NCB of I1 are present in CB. The correlation coefficient between CB and NCB *P*_b,i_(*i*) vectors is surprisingly strong being *r* = 0.97. Thus, competition from the peptide makes little changes in I1 binding.

**Table 1:** Impα Amino Acids with the Strongest Affinities toward the coreNLS Peptide^a^^,^^b^

rank	noncompeting^15^		competing with I1		competing with I2	
	amino acid *i*	*P*_b,p_(*i*)	amino acid *i*	*P*_b,p_(*i*)	amino acid *i*	*P*_b,p_(*i*)
1	**Trp161**	0.99 ± 0.00	***Trp161***	0.89 ± 0.01	***Trp161***	0.88 ± 0.00
2	**Ser79**	0.97 ± 0.00	Asp200	0.74 ± 0.00	***Ser79***	0.78 ± 0.02
3	**Asn118**	0.97 ± 0.00	Glu196	0.66 ± 0.00	***Asp122***	0.76 ± 0.01
4	**Gly80**	0.95 ± 0.00	***Trp114***	0.64 ± 0.01	***Gly80***	0.76 ± 0.02
5	**Ala78**	0.95 ± 0.00	***Ser79***	0.51 ± 0.02	***Asn118***	0.76 ± 0.02
6	**Asp122**	0.95 ± 0.00	***Trp72***	0.43 ± 0.01	Gly121	0.69 ± 0.02
7	**Thr85**	0.95 ± 0.00	***Asn118***	0.42 ± 0.01	***Ala78***	0.68 ± 0.01
8	**Trp114**	0.92 ± 0.00	**Asn158**	0.41 ± 0.00	***Thr85***	0.67 ± 0.01
9	**Thr81**	0.86 ± 0.00	Arg157	0.35 ± 0.00	Asp200	0.59 ± 0.00
10	**Trp72**	0.86 ± 0.00	**Asp122**	0.34 ± 0.01	Glu196	0.59 ± 0.00

a Amino acids in bold belong to the coreNLS native binding site.

b Italicized amino acids also appear in NCB.

Table 2 examines the binding interactions formed by the coreNLS amino acids with impα. It follows from the table that in CB the peptide loses about half of all interactions with impα, reducing the number of binding contacts ⟨*C*_b_⟩ from 28.1 to 12.9. The fraction of retained native contacts *P*_n_ is reduced almost 5-fold, from 0.62 in NCB to 0.14 in CB, while the fraction of nonnative binding interactions *P*_nn_ doubles. The largest losses of native interactions measured by *P*_n_(*j*) occur at the positions *j* = Lys6 (Δ*P*_n_(Lys6) = *P*_n_(Lys6;CB) – *P*_n_(Lys6, NCB) = −0.70), Lys9 (−0.51), Pro8 (−0.53), and Lys7 (−0.57). As a result, the peptide binding and native interaction along the sequence are greatly reduced compared with NCB. Specifically, the amino acids Lys6-Lys9 retain, on average, only 17% of native interactions, while Lys10-Glu11 retain 10%. These two coreNLS regions form, per residue, 2.0 and 1.8 binding contacts. This is in contrast to NCB, where Lys6-Lys9 retain 73% of native interactions and maintain 5.6 binding contacts, whereas Lys10-Glu11 retain 29% and 2.9, respectively. To illustrate the impact of I1, we consider the binding of coreNLS peptide to impα tryptophan residues, Trp72, Trp 114, and Trp161. In NCB, the coreNLS Lys7 forms a stable π–cation contact with the side chains of Trp161 in addition to π–cation interactions of Lys9 with Trp72 and Trp114. Furthermore, Lys9 resides with a probability of 0.58 in the cage formed by Trp72 and Trp114. These specific interactions are completely wiped out by I1 as their probabilities drop below 0.11, and the probability for Lys9 to occupy the Trp cage vanishes. (However, coreNLS Lys and, particularly, Pro still bind to Trp side chains without forming cages or π–cation interactions.) It is of note that I1 interference induces strong nonnative binding interactions formed with anionic Glu196 and Asp200 and with cationic Arg157. Strikingly, the first two form the largest number of hydrogen bonds with the peptide (0.78 and 0.68), which exceed those formed by any other impα amino acid at least 3-fold. Thus, I1 forces the peptide to acquire new electrostatic interactions and hydrogen bonds outside of the coreNLS binding pose. To compare the coreNLS dimensions in CB and NCB, we display in Figure S7 (SI1) the probability distributions *P*(*R*_g_) of the radius of gyration of coreNLS peptide *R*_g_. It is seen that *P*(*R*_g_) computed for CB and NCB has similar unimodal shapes peaking at about 7 Å. Therefore, I1 does not appreciably change the dimensions of the peptide. Taken together, the RMSD and free energy computations, the analysis of the interactions between the peptide and impα, and the locations of the peptide binding demonstrate that, although the coreNLS peptide still binds to the major NLS binding site, I1 interference completely obliterates its native pose (Figure 1c).

**Table 2:** Binding Interactions between the coreNLS Amino Acids and Impα Protein

	*j* = Lys6	*j* = Lys7	*j* = Pro8	*j* = Lys9	*j* = Lys10	*j* = Glu11	coreNLS peptide
noncompeting^15^							
*P*_n_	0.86 ± 0.01	0.67 ± 0.01	0.75 ± 0.01	0.65 ± 0.00	0.21 ± 0.00	0.36 ± 0.00	0.62 ± 0.02
*P*_nn_	0.26 ± 0.00	0.03 ± 0.01	0.11 ± 0.01	0.24 ± 0.01	0.55 ± 0.08	0.75 ± 0.02	0.29 ± 0.02
⟨*C*_b_⟩	10.6 ± 0.1	4.1 ± 0.2	2.6 ± 0.2	5.1 ± 0.2	2.8 ± 0.3	2.9 ± 0.0	28.1 ± 0.4
competing with I1							
*P*_n_	0.18 ± 0.03	0.16 ± 0.02	0.24 ± 0.03	0.08 ± 0.02	0.08 ± 0.01	0.12 ± 0.02	0.14 ± 0.01
*P*_nn_	0.56 ± 0.03	0.46 ± 0.01	0.59 ± 0.04	0.78 ± 0.06	0.76 ± 0.10	0.86 ± 0.04	0.64 ± 0.06
⟨*C*_b_⟩	3.6 ± 0.4	1.8 ± 0.1	1.8 ± 0.1	2.0 ± 0.5	2.0 ± 0.3	1.8 ± 0.3	12.9 ± 0.9
competing with I2							
*P*_n_	0.35 ± 0.06	0.20 ± 0.03	0.16 ± 0.03	0.05 ± 0.00	0.03 ± 0.00	0.05 ± 0.00	0.17 ± 0.03
*P*_nn_	0.33 ± 0.07	0.47 ± 0.07	0.77 ± 0.09	0.88 ± 0.07	0.92 ± 0.07	0.92 ± 0.02	0.64 ± 0.15
⟨*C*_b_⟩	4.7 ± 0.5	2.2 ± 0.1	2.1 ± 0.6	2.5 ± 0.8	2.3 ± 0.4	1.3 ± 0.3	15.1 ± 0.7

**Figure 4:**
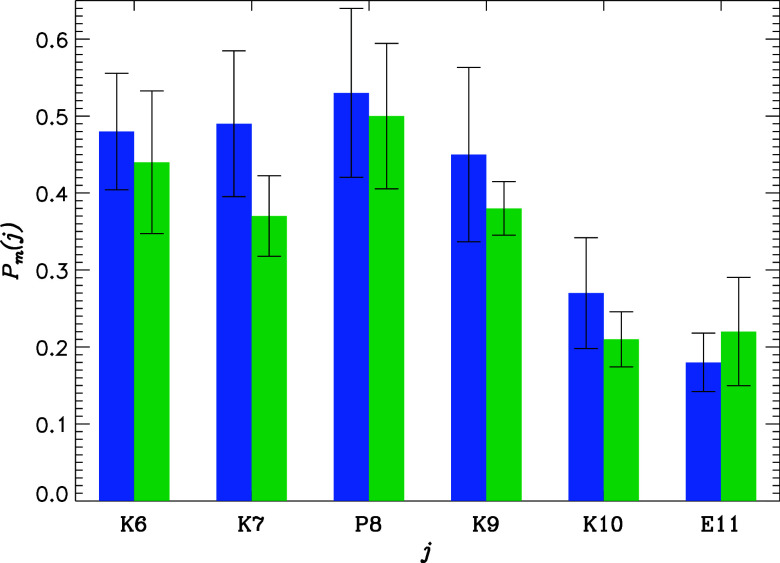
Probabilities of the inhibitor binding *P*_m_(*j*) to the coreNLS amino acids *j* observed in the CB simulations. Data in blue and green represent I1 and I2, respectively. The figure underscores a strong preference for both inhibitors to interact with the N-terminus of the peptide compared with the C-terminus.

To explore the mechanism of I1 interference, we checked if the coreNLS peptide and I1 inhibitor compete for binding to the same impα amino acids. The survey of Tables 1 and S1 indicates that, in CB, 50% of top ten binding impα amino acids are shared between the peptide and the inhibitor. In NCB, this fraction of common impα amino acids is 60%,^10,15^ and all five shared in CB appear in NCB simulations. If the competition for binding to the same impα amino acids is the basis for inhibition, then one may expect a correlation between the inhibitor probabilities *P*_b,i_(*i*) of binding to impα amino acids *i* and the difference Δ*P*_b,p_(*i*) = *P*_b,p_(*i*;CB) – *P*_b,p_(*i*;NCB), which measures the changes between peptide CB and NCB. However, the respective correlation coefficient is only −0.11 implying that I1 binding to impα does not guide the inhibition. This conclusion remains intact if we replace Δ*P*_b,p_(*i*) with the changes in native binding interactions formed by impα amino acids with the peptide caused by I1. Specifically, *P*_b,i_(*i*) does not correlate with Δ*P*_n_(*i*) = *P*_n_(*i*;CB) – *P*_n_(*i*;NCB), where *P*_n_(*i*;CB) and *P*_n_(*i*;NCB) are the fractions of native binding contacts formed by impα amino acid *i* in CB and NCB. The respective correlation coefficient is *r* = 0.13. If the interactions of I1 with impα are not responsible for the loss of native peptide binding, then what could be the origin of I1 inhibition activity? We hypothesize that it is due to I1 interfering with the coreNLS binding interactions that result in the effective “masking” of its amino acids. We showed above that I1 is almost always bound to the peptide. Furthermore, according to Figure 4, I1 primarily binds to Lys6-Lys9 amino acids in the coreNLS peptide, which are exactly the positions exhibiting the largest losses in native binding interactions (Table 2). In fact, we can evaluate the correlation between the probability *P*_m_(*j*) of the inhibitor binding to the coreNLS amino acids *j* in Figure 4 and the loss of native interactions formed by *j* Δ*P*_n_(*j*) = *P*_n_(*j*;CB) – *P*_n_(*j*;NCB). The resulting correlation is surprisingly strong, being *r* = −0.85 (significance *p* = 0.03). Interestingly, the correlation coefficient between *P*_m_(*j*) and the loss in any binding interactions formed by *j* ⟨Δ*C*(*j*)⟩ = ⟨*C*(*j*;CB) ⟩ – ⟨*C*(*j*;NCB)⟩ is very weak, dropping to *r* = −0.39. These observations suggest that masking the coreNLS peptide I1 interferes with its native binding interactions. However, I1 does not appear to compete with the coreNLS peptide for binding to impα.

### CB of the coreNLS Peptide and the Inhibitor I2 to Impα

Our next objective was to examine the CB of the coreNLS peptide and the inhibitor I2 to impα. Again, the NCB of the coreNLS or of I2 to impα served as a reference.^15^ Similar to I1 CB the coreNLS peptide remains almost always bound to the impα major NLS binding site (*P*_b_ ≃ 0.96 ± 0.01). Also, because I2 binds to the coreNLS and impα with the probabilities 0.91 ± 0.00 and 0.99 ± 0.00, it is, as I1, expected to interfere with the peptide–impα interactions. Indeed, Figure 2b shows that the probability distribution *P*(*RMSD*) of the peptide RMSD values is broadened and dramatically shifted to larger RMSDs compared with the NCB. The CB *P*(*RMSD*) implicates a heterogeneous ensemble of the peptide bound poses with most *RMSD* > 10 Å. Furthermore, clustering of the coreNLS poses reveals a multitude of small clusters, none of which capture more than 8% of the poses. The cluster closest to the native PDB structure has an average RMSD of 3.6 Å and a population fraction of 0.05. These findings are in line with I1 CB data, but in sharp contrast to the coreNLS NCB, in which the native cluster captures 67% of poses and has the average RMSD of 2.5 Å.^15^ Overall, the average RMSD for all CB peptide poses raises to <RMSD> = 11.9 ± 0.3 Å that is 2.6-fold higher than in NCB and about the same as for the I1 CB. Thus, as with I1, the inhibitor I2 disorders the coreNLS binding poses (Figure 1d).

Further insights are gleaned from the free energies *G*(*C*_n_, *C*_nn_) = −*RT* ln *P*(*C*_n_, *C*_nn_) in Figure 3b. In contrast to the NCB free energy landscape, the I2 interference creates three new low free energy basins but none with a high fraction of native interactions. In the state CB1, the number of native contacts *C*_n_ is reduced to ∼10, while the number of nonnative contacts *C*_nn_ is ∼5. The two other states, CB2 and CB3, feature virtually no native interactions and either few (*C*_nn_ ∼ 3 in CB2) or a large number of nonnative contacts (*C*_nn_ ∼ 21 in CB3). Compared with I1 CB, the free energy for I2 CB reveals a fragmented coreNLS binding ensemble with some native interactions retained. To map the coreNLS binding site, we used the probabilities *P*_b,p_(*i*), of which the top ten are listed in Table 1. Among them, seven are native, and all of these seven are retained from NCB. There are three nonnative impα amino acids, two of which are negatively charged, Glu196 and Asp200. Interestingly, in the I2 CB, the five retained native impα amino acids occupy the top five positions in Table 1, whereas in the I1 CB the top five feature two nonnative Glu196 and Asp200 residues, which appear in the bottom of the I2 list. Furthermore, among the five top binding impα amino acids seen in I2 CB and NCB, four are common, whereas for I1 there are only two. Thus, although the probability distribution *P*(*RMSD*) in Figure 2b implicates a loss of native binding pose, Table 1 demonstrates that I2 does not perturb native coreNLS binding as severely as I1. In fact, the correlation coefficient *r* computed between *P*_b,p_(*i*) probabilities from CB and NCB is 0.48, which is twice that for I1. The top ten probabilities *P*_b,i_(*i*) probing the CB of I2 show strikingly that only two amino acids are preserved from NCB (see Table S1 in SI1). In agreement, the correlation coefficient between the CB and NCB *P*_b,i_(*i*) vectors is *r* = −0.18. Thus, opposite to I1, competition from the peptide makes sweeping changes in the binding of I2 inhibitor.

The loss of native coreNLS binding interactions is detailed in Table 2. Similar to I1, competition from I2 forces the coreNLS peptide to lose almost 50% of its binding interactions with impα as ⟨*C*_b_⟩ decreases from 28.1 to 15.1. The fraction of retained native contacts *P*_n_ in CB is reduced almost 4-fold to 0.17, while the fraction of nonnative binding interactions *P*_nn_ doubles. These results are on par with those of I1. The largest losses of native interactions are observed at the positions *j* = Lys9 (Δ*P*_n_(*j*) = −0.60), Pro8 (−0.59), Lys6 (−0.51), and Lys7 (−0.47). As a result, the peptide forms more even numbers of binding and native interactions along the sequence, but the exception is Lys6, which keeps the largest fraction of native (0.35) and binding (4.7) interactions among all coreNLS amino acids. Similar to I1 CB the peptide C-terminus (Lys10, Glu11) maintains few (4%) native interactions, whereas the N-terminus (Lys6-Lys9) preserves a larger fraction (18%). Also similar to I1 the π-cation interactions between the coreNLS Lys amino acids and impα tryptophans are erased as their probabilities do not exceed 0.07. I2 interference also blocks Lys from residing in the Trp cages. In short, I2 CB differs from the NCB, but to a lesser extent than I1 CB does. Finally, Figure S7 in SI1 compares the probability distributions *P*(*R*_g_) of the radius of gyration of coreNLS peptide *R*_g_. It is evident that I2 interference changes *P*(*R*_g_) by making it bimodal and shifting it to a smaller *R*_g_. Therefore, I2 competition forces the peptide to partially collapse.

It follows from Tables 1 and S1 that only 30% of the top ten impα binding amino acids are shared between the peptide and the inhibitor CB, which is smaller than for I1. This outcome can be expected because opposite to I1 the NCB of I2 is off-target being shifted away from the coreNLS binding site.^10^ Following the analysis performed for I1, we computed the correlation between the inhibitor probabilities *P*_b,i_(*i*) of binding to impα and the difference in peptide binding affinities to impα amino acids Δ*P*_b,p_(*i*) (see previous section). The resulting correlation coefficient of −0.47. If we consider the correlation between *P*_b,i_(*i*) and the changes in native peptide binding affinities Δ*P*_n_(*i*), then *r* = −0.36. These computations suggest that in contrast to I1, the interactions of I2 with impα weakly contribute to destabilizing coreNLS binding. This finding is consistent with overall stronger binding of I2 to impα compared to I1.^10^ Another, more important factor responsible for the inhibitory activity of I2 and the one shared with I1 is the masking of the coreNLS peptide. Figure 4 demonstrates that I2 primarily binds to the coreNLS Lys6-Lys9 amino acids, which are also those exhibiting the largest loss of native interactions (Table 2). Specifically, the correlation coefficient between the probability *P*_m_(*j*) of inhibitor binding to the coreNLS amino acids *j* and the loss of native interactions Δ*P*_n_(*j*) is *r* = −0.89 (significance *p* = 0.02), which is as strong as for I1. Note that as for I1 the correlation between *P*_m_(*j*) and the loss in binding interactions < Δ*C*(*j*) > is very weak (*r* = −0.35).

### Comparison of the Inhibitor Binding Mechanisms and Broader Implications

Taking our analyses together, we can summarize the mechanisms of inhibition as follows. The outcomes of I1 and I2 interference with the coreNLS binding to impα are largely similar manifested in a complete loss of native binding pose for the peptide. For both ligands, this outcome engenders from masking the coreNLS amino acids and primarily targeting the coreNLS native binding interactions. The plausible source of this outcome is the distribution of binding interactions. On an average, I1 binds to a peptide amino acid with the probability 0.40. In contrast, I1 binds to an impα amino acid (involved in binding) with the probability 0.21. The corresponding values for I2 are 0.35 and 0.23. Thus, as illustrated in the “Table of contents” graphics, I1 forms twice stronger interactions with coreNLS rather than impα amino acids. As a result, the I1 effect on peptide binding interactions is more pronounced than on the binding interactions formed by impα amino acids. To a lesser extent, the same conclusions apply to I2. In summary, masking is a consequence of the diffusive nature of inhibitor binding to impα,^10^ in which inhibitor, while making few strong interactions with the coreNLS, forms multiple weak interactions with impα. Consequently, masking does not necessarily imply stronger overall binding of inhibitors to the coreNLS peptide compared to impα.

The inhibitors’ modes of action also show differences. I1 binding to impα amino acids does not appear to drive the inhibition of coreNLS-impα interactions. Furthermore, CB induces strong nonnative interactions into coreNLS binding to impα. This outcome is consistent with our earlier study of I1 NCB, which showed that I1 primarily targets the NLS binding site but forms relatively weak binding interactions with impα (an “on-target nonspecific” ligand).^10^ In contrast to I1, I2 interactions with impα amino acids weakly contribute to its inhibitory activity. Also, opposite to I1, the I2 CB retains more native coreNLS binding interactions. These observations agree well with our earlier study of I2 NCB, where we found I2 targeting the edges of the NLS binding site and forming relatively strong binding interactions with impα (an “off-target specific” ligand).^10^ Additional supporting evidence came from energy computations. The average energy of I1 and I2 interactions with impα are −26.8 and −33.2 kcal/mol. Since I2 interactions with impα are stronger than those of I1, we can expect them to contribute to I2 inhibitory activity. Analysis of the distributions of the coreNLS radius of gyration revealed that I2 but not I1 forces a partial collapse of the peptide bound to impα. These differences in ligand inhibitory mechanisms must be ultimately related to their different hydrophobicities (Figure 1a). Indeed, due to the presence of two extra hydrocarbon groups and elimination of an amino group, I2 is more apolar than I1. However, independent of these differences, both inhibitors share the same basic inhibitory action based on masking the NLS peptide.

Although the bound free energy landscapes in Figure 3 provide insights into inhibitor interference, they cannot estimate changes in the free energy of binding of the coreNLS peptide to impα ΔΔ*G*_b_(*x*) caused by inhibitor *x*. To compute ΔΔ*G*_b_(*x*), we applied the MM-GBSA approach outlined in Models and Methods.^30^ The free energy data are presented in Tables S2 and S3 in SI1. It follows from these tables that the competition from I1 and I2 increases the binding free energy of the coreNLS peptide by 19.4 and 8.5 kcal/mol, respectively (see SI1 for an additional discussion). The decomposition of ΔΔ*G*_b_(*x*) with respect to the terms listed in eq 1 in Table S4 (see SI1) shows that I1 decreases the gain in molecular mechanical energy upon binding by ΔΔ*E*_mm_ = 148.5 kcal/mol, which is almost compensated by the reduction in the loss of polar solvation energy ΔΔ*G*_solv,p_ = −131.8 kcal/mol. For I2 the respective quantities are ΔΔ*E*_mm_ = 73.8 kcal/mol and ΔΔ*G*_solv,p_ = −68.3 kcal/mol. These calculations have several implications. First, consistent with our structural analysis, both inhibitors strongly destabilize the coreNLS binding to impα. The inhibitors primarily compromise the gain in molecular mechanical energy occurring upon the coreNLS binding to impα, but also reduce the loss in polar solvation interactions. Second, I1 destabilizes the coreNLS bound state more severely than does I2. This outcome results from I1 reducing the molecular mechanical energy gain ΔΔ*E*_mm_ upon the coreNLS binding to impα much more severely than I2. Free energy computations are in line with our previous findings that contrary to I1 the inhibitor I2 is off-target ligand binding on the edges of the major NLS binding site. Importantly, structural analysis in Tables 1 and 2 supports weaker inhibiting activity of I2. Indeed, all top ten probabilities *P*_b,p_(*i*) of forming contacts between impα amino acids *i* and the coreNLS peptide in CB with I2 exceed 0.59, whereas in I1 CB only four *P*_b,p_(*i*) do so (Table 1). In summary, the analysis of binding free energies suggests that the inhibiting activity of I1 is stronger than that of I2 as it was already alluded in the previous sections.

**Figure 5:**
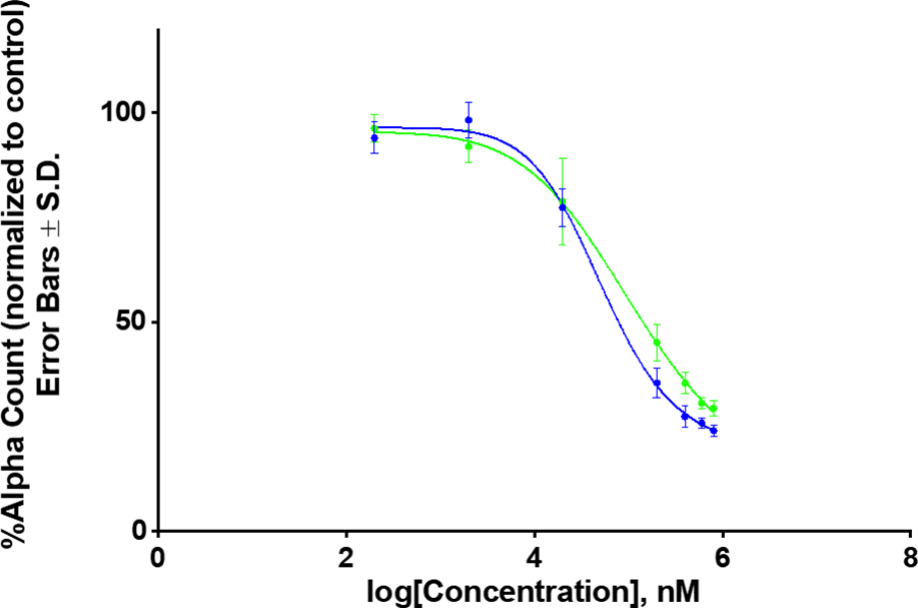
Alpha counts dose–response inhibition assay. The *y*-axis is the percentage of the alpha counts normalized to the DMSO control. Error bars represent the standard deviation and *n* = 6 for each concentration. The IC50 values are reported in μM ± SEM (see the text). In agreement with the free energy analysis, the inhibition curves suggest that I1 (in blue) is a stronger inhibitor than I2 (in green).

It is imperative to provide an experimental verification of I1 and I2 inhibiting activities. To this end, we have performed experimental AlphaScreen measurements following the methodology described in Supporting Information 2 (SI2). In these experiments, we probed the inhibitory effect of the ligands I1 and I2 on the binding of VEEV NLS peptide to impα. The normalized Alpha counts were fitted with the four-parameter nonlinear regression curve AlphaCount = AlphaCount_max_ + (AlphaCount_min_ – AlphaCount_max_)/(1 + (IC50/log[inhibitor])^HillSlope^). The resulting plots are shown in Figure 5, which yield the IC50 values for I1 and I2 inhibitors as 53 ± 6 and 98 ± 30 μM, respectively. Since lower IC50 implicates stronger inhibition of NLS binding to impα, the experimental data qualitatively confirm our conclusions based on free energy computations and structural analysis. It must be noted though that the IC50 values cannot be quantitatively compared to our free energy estimates because the former might be effected by multiple binding locations or interligand interactions in addition to binding affinity of a single ligand itself. More generally, the experimental data support the inhibition activities of I1 and I2.

If masking of NLS amino acids primarily drives the inhibiting activity of I1 and I2, would this translate into the specificity of these ligands toward the VEEV NLS? Experimental studies have shown that the free energy of binding of NLS to impα is about −10 kcal/mol,^11^ although it can be reduced to ≈ – 5 kcal/mol for some human proteins.^12^ Recent molecular dynamics studies of SV40 NLS peptide PKKKRKV and the cryptic NLS from the fatty acid binding protein suggested that both experience significant structural fluctuations while bound to the impα major NLS binding site.^35^ In particular, the bound ensemble of SV40 large T-antigen NLS peptide fragments into 27 clusters defined with the cutoff of 0.5 Å, although the peptide generally retains the interactions with three impα tryptophan amino acids and the ionic contact of P2 with impα. Furthermore, the NLS fragment from the critical pluripotency factor Oct4 adopts a bound pose in the impα major site, which is inconsistent with that of VEEV NLS. For example, P5 Arg escapes the cage formed by the tryptophan side chains.^36^ In contrast to these NLS fragments, the VEEV coreNLS peptide retains native-like binding pose in the major NLS binding site.^15^ It is notable that none of these other NLS sequences harbor the VEEV minNLS sequence KKPK. Moreover, our previous database analysis showed that the sequence KKPK appears among 242 distinct Swiss-Prot human entries, with only five containing the coreNLS sequence KKPKKE.^15^ Strikingly, only one of these proteins is predicted to interact with the impα isoforms. Finally, in this article, we argue that the inhibitors primarily interfere with the coreNLS rather than impα binding interactions. Therefore, one may hypothesize that VEEV inhibitors are specific to VEEV NLS sequence, in part because it is so rare among proteins. In fact, prior experimental studies offer some support for I1 selectivity.^8^ In this respect, it will be prudent to repeat our CB simulations with the I1 and SV40 NLS peptide.

Because the disordered coreNLS peptide adopts a specific conformation upon NCB to impα, it appears to follow an “induced fit” binding mechanism.^37^ In competitive simulations, both inhibitors destabilize the native pose of the coreNLS peptide within the impα major NLS binding site, but they do not prevent its disordered and diffusive binding. This outcome suggests that the inhibitors target the “induced fit” binding, but not the binding itself. If inhibitors are also specific to VEEV coreNLS peptide, then by weakening the VEEV NLS binding to impα they allow host cargo proteins to outcompete VEEV NLS at the impα major NLS binding site.

## Conclusions

In this article, we investigated the mechanisms by which the two ligands from the G281–1485 family, I1 and I2, interfere with VEEV NLS binding to impα cargo transport protein. To this end, we performed all-atom REST simulations probing the CB of the VEEV coreNLS peptide and I1 or I2 ligand to the impα major NLS binding site. As a reference, we used our previous simulations, which studied NCB of the coreNLS peptide or the inhibitors to impα. Our main finding is that both inhibitors abrogate native binding of the coreNLS peptide, forcing it to adopt a manifold of nonnative loosely bound poses within the impα major NLS binding site. We found that the inhibitors primarily destabilize the coreNLS binding by masking its amino acids rather than competing for binding to impα. Due to unequal hydrophobicities, there are differences in I1 and I2 inhibitory activities. In particular, opposite to I1, the interactions of I2 with impα weakly contribute to its inhibitory activity. Also, because, upon binding, I2 localizes on the edge of the major NLS binding site, it inhibits fewer coreNLS native binding interactions than I1. Our structural analysis is supported by the computations of the free energies of the coreNLS peptide binding to impα with or without competition from the inhibitors. We showed that both inhibitors reduce the free energy gain from the coreNLS binding, although I1 causes a significantly larger loss in the coreNLS binding affinity than I2. To test our simulations, we performed AlphaScreen experiments measuring IC50 values for both inhibitors. Consistent with in silico results, the IC50 value for I1 was found to be lower than that for I2. We hypothesized that the inhibitory action of I1 and I2 ligands might be specific to the NLS from VEEV’s capsid protein. More generally, the main outcome of our investigation is that the ligands binding diffusively without well-defined binding poses may still act as inhibitors of protein–protein interactions.

## Data Availability

NAMD is available at https://www.ks.uiuc.edu/Research/namd/. VMD is available at https://www.ks.uiuc.edu/Research/vmd/. Initial structures, topology files, and NAMD configuration files are available at https://github.com/KlimovLab/CB. Codes used for data analysis are available from the authors upon request.

## Supporting Information Available

Walks of REST replicas, replica mixing parameter, replica exchange rates, numbers of binding contacts, contact root-mean-squared deviations, and peptide root-mean-squared deviations for convergence analysis, top binding impα amino acids, probability distributions of peptide radius of gyration, binding free energies with their components and contributions to their changes (PDF)
Compound synthesis procedures (PDF)

## References

[ref1] Zacks M. A.; Paessler S. Encephalitic alphaviruses. Vet. Microbiol. 2010, 140, 281–286. 10.1016/j.vetmic.2009.08.023. 19775836 PMC2814892

[ref2] Lundberg L.; Carey B.; Kehn-Hall K. Venezuelan equine encephalitis virus capsid—the clever caper. Viruses 2017, 9, 279 10.3390/v9100279. 28961161 PMC5691631

[ref3] Aguilar P. V.; Estrada-Franco J. G.; Navarro-Lopez R.; Ferro C.; Haddow A. D.; Weaver S. C. Endemic Venezuelan equine encephalitis in the Americas: Hidden under the dengue umbrella. Future Virol. 2011, 6, 721–740. 10.2217/fvl.11.50. 21765860 PMC3134406

[ref4] Atasheva S.; Garmashova N.; Frolov I.; Frolova E. Venezuelan equine encephalitis virus capsid protein inhibits nuclear import in mammalian but not in mosquito cells. J. Virol. 2008, 82, 4028–4041. 10.1128/JVI.02330-07. 18256144 PMC2293000

[ref5] Atasheva S.; Fish A.; Fornerod M.; Frolova E. I. Venezuelan equine encephalitis virus capsid protein forms a tetrameric complex with CRM1 and importin α/β that obstructs nuclear pore complex function. J. Virol. 2010, 84, 4158–4171. 10.1128/JVI.02554-09. 20147401 PMC2863722

[ref6] Kosyna F. K.; Depping R. Controlling the gatekeeper: Therapeutic targeting of nuclear transport. Cells 2018, 7, 221 10.3390/cells7110221. 30469340 PMC6262578

[ref7] Atasheva S.; Kim D. Y.; Frolova E. I.; Frolov I. Venezuelan equine encephalitis virus variants lacking transcription inhibitory functions demonstrate highly attenuated phenotype. J. Virol. 2015, 89, 71–82. 10.1128/JVI.02252-14. 25320296 PMC4301144

[ref8] Thomas D. R.; Lundberg L.; Pinkham C.; Shechter S.; DeBono A.; Baell J.; Wagstaff K. M.; Hick C. A.; Kehn-Hall K.; Jans D. A. Identification of novel antivirals inhibiting recognition of Venezuelan equine encephalitis virus capsid protein by the importin α/β 1 heterodimer through high-throughput screening. Antiviral Res. 2018, 151, 8–19. 10.1016/j.antiviral.2018.01.007. 29337164

[ref9] Simpson M.; Poulsen S. A. An overview of Australia’s compound management facility: The Queensland Compound Library. ACS Chem. Biol. 2014, 9, 28–33. 10.1021/cb400912x. 24432754

[ref10] Delfing B. M.; Olson A.; Laracuente X. E.; Foreman K. W.; Paige M.; Kehn-Hall K.; Lockhart C.; Klimov D. K. Binding of Venezuelan Equine Encephalitis Virus Inhibitors to Importin-alpha Receptors Explored with All-Atom Replica Exchange Molecular Dynamics. J. Phys. Chem. B 2023, 127, 3175–3186. 10.1021/acs.jpcb.3c00429. 37001021 PMC10358320

[ref11] Hodel M. R.; Corbett A. H.; Hodel A. E. Dissection of a nuclear localization signal. J. Biol. Chem. 2001, 276, 1317–1325. 10.1074/jbc.M008522200. 11038364

[ref12] Faerch O.; Worth R.; Achilonu I.; Dirr H. Nuclear localisation sequences of chloride intracellular channels 1 and 4 facilitate nuclear import via interactions with import mediator importin-α: An empirical and theoretical perspective. J. Mol. Recognit. 2022, 36, e2996 10.1002/jmr.2996. 36175369 PMC10078197

[ref13] Christie M.; Chang C.-W.; Róna G.; Smith K. M.; Stewart A. G.; Takeda A. A.-S.; Fontes M. R. M.; Stewart M.; Vértessy B. G.; Forwood J. K.; et al. Structural biology and regulation of protein import into the nucleus. J. Mol. Biol. 2016, 428, 2060–2090. 10.1016/j.jmb.2015.10.023. 26523678

[ref14] Fan F. Crystal structure analysis of Venezuelan equine encephalitis virus capsid protein NLS and importin alpha, 2012.

[ref15] Delfing B. M.; Laracuente X. E.; Olson A.; Foreman K. W.; Paige M.; Kehn-Hall K.; Lockhart C.; Klimov D. K. Binding of Viral Nuclear Localization Signal Peptides to Importin-α Nuclear Transport Protein. Biophys. J. 2023, 122, 3476–3488. 10.1016/j.bpj.2023.07.024. 37542371 PMC10502480

[ref16] Prlić A.; Bliven S.; Rose P. W.; Bluhm W. F.; Bizon C.; Godzik A.; Bourne P. E. Pre-calculated protein structure alignments at the RCSB PDB website. Bioinformatics 2010, 26, 2983–2985. 10.1093/bioinformatics/btq572. 20937596 PMC3003546

[ref17] Dias S. M. G.; Wilson K. F.; Rojas K. S.; Ambrosio A. L. B.; Cerione R. A. The molecular basis for the regulation of the cap-binding complex by the importins. Nat. Struct. Mol. Biol. 2009, 16, 930–937. 10.1038/nsmb.1649. 19668212 PMC2782468

[ref18] Pang X.; Zhou H. X. Design Rules for Selective Binding of Nuclear Localization Signals to Minor Site of Importin α. PLoS One 2014, 9, e91025 10.1371/journal.pone.0091025. 24609064 PMC3946659

[ref19] Huang J.; Rauscher S.; Nawrocki G.; Ran T.; Feig M.; de Groot B. L.; Grubmuller H.; MacKerell A. D. Jr CHARMM36m: An improved force field for folded and intrinsically disordered proteins. Nat. Methods 2017, 14, 71–73. 10.1038/nmeth.4067. 27819658 PMC5199616

[ref20] Best R. B.; Zhu X.; Shim J.; Lopes P. E. M.; Mittal J.; Feig M.; MacKerell A. D. Optimization of the additive CHARMM all-atom protein force field targeting improved sampling of the backbone ϕ,ψ and side-chain χ1 and χ2 dihedral angles. J. Chem. Theory Comput. 2012, 8, 3257–3273. 10.1021/ct300400x. 23341755 PMC3549273

[ref21] Vanommeslaeghe K.; Raman E. P.; MacKerell A. D. Automation of the CHARMM general force field (CGenFF) II: Assignment of bonded parameters and partial atomic charges. J. Chem. Inf. Model. 2012, 52, 3155–3168. 10.1021/ci3003649. 23145473 PMC3528813

[ref22] Jorgensen W. L.; Chandrasekhar J.; Madura J. D.; Impey R. W.; Klein M. L. Comparison of simple potential functions for simulating liquid water. J. Chem. Phys. 1983, 79, 926–935. 10.1063/1.445869.

[ref23] MacKerell A. D.; Bashford D.; Bellott M.; Dunbrack R. L.; Evanseck J. D.; Field M. J.; Fischer S.; Gao J.; Guo H.; Ha S.; et al. All-Atom Empirical Potential for Molecular Modeling and Dynamics Studies of Proteins. J. Phys. Chem. B 1998, 102, 3586–3616. 10.1021/jp973084f. 24889800

[ref24] Wang L.; Friesner R. A.; Berne B. J. Replica Exchange with Solute Scaling: A More Efficient Version of Replica Exchange with Solute Tempering (REST2). J. Phys. Chem. B 2011, 115, 9431–9438. 10.1021/jp204407d. 21714551 PMC3172817

[ref25] Smith A. K.; Lockhart C.; Klimov D. K. Does Replica Exchange with Solute Tempering efficiently sample Aβ peptide conformational ensembles?. J. Chem. Theor. Comp. 2016, 12, 5201–5214. 10.1021/acs.jctc.6b00660. 27560127

[ref26] Philips J. C.; Hardy D. J.; Maia J. D. C.; Stone J. E.; Ribeiro J. V.; Bernardi R. C.; Buch R.; Fiorin G.; Hénin J.; Jiang W.; McGreevy R.; et al. Scalable molecular dynamics on CPU and GPU architectures with NAMD. J. Chem. Phys. 2020, 153, 044130 10.1063/5.0014475. 32752662 PMC7395834

[ref27] Humphrey W.; Dalke A.; Schulten K. VMD Visual molecular dynamics. J. Mol. Graph. 1996, 14, 33–38. 10.1016/0263-7855(96)00018-5. 8744570

[ref28] Daura X.; Gademann K.; Jaun B.; Seebach D.; van Gunsteren W. F.; Mark A. E. Peptide Folding: When Simulation Meets Experiment. Angew. Chem., Int. Ed. 1999, 38, 236–240. 10.1002/(SICI)1521-3773(19990115)38:1/2<236::AID-ANIE236>3.0.CO;2-M.

[ref29] Castro-Alvarez A.; Costa A. M.; Vilarrasa J. The Performance of Several Docking Programs at Reproducing Protein–Macrolide-Like Crystal Structures. Molecules 2017, 22, 136 10.3390/molecules22010136. 28106755 PMC6155922

[ref30] Vergara-Jaque A.; Comer J.; Monsalve L.; González-Nilo F. D.; Sandoval C. Computationally efficient methodology for atomic-level characterization of dendrimer-drug complexes: a comparison of amine- and acetyl-terminated PAMAM. J. Phys. Chem. B 2013, 117, 6801–6813. 10.1021/jp4000363. 23642174

[ref31] Onufriev A.; Bashford D.; Case D. A. Exploring protein native states and large-scale conformational changes with a modified Generalized Born model. Prot. Struct. Funct. Bioinf. 2004, 55, 383–394. 10.1002/prot.20033. 15048829

[ref32] Smith A. K.; Klimov D. K. De novo aggregation of Alzheimer’s Aβ 25–35 peptides in a lipid bilayer. Sci. Rep. 2019, 9, 7161 10.1038/s41598-019-43685-7. 31073226 PMC6509337

[ref33] Trott O.; Olson A. J. AutoDock Vina: Improving the speed and accuracy of docking with a new scoring function, efficient optimization, and multithreading. J. Comput. Chem. 2010, 31, 455–461. 10.1002/jcc.21334. 19499576 PMC3041641

[ref34] Eberhardt J.; Santos-Martins D.; Tillack A. F.; Forli S. AutoDock Vina 1.2.0: New docking methods, expanded force field, and Python bindings. J. Chem. Inf. Model. 2021, 61, 3891–3898. 10.1021/acs.jcim.1c00203. 34278794 PMC10683950

[ref35] Amber-Vitos O.; Kucherenko N.; Nachliel E.; Gutman M.; Tsfadia Y. The Interaction of FABP with Kapα. PLoS One 2015, 10, e0132138 10.1371/journal.pone.0132138. 26284534 PMC4540411

[ref36] Okuyama T.; Yamagishi R.; Shimada J.; Ikeda M.; Maruoka Y.; Kaneko H. Structural and mechanistic insights into nuclear transport and delivery of the critical pluripotency factor Oct4 to DNA. J. Biomol. Str. Dyn. 2018, 36, 767–778. 10.1080/07391102.2017.1289124. 28166455

[ref37] Arai M.; Sugase K.; Dyson H. J.; Wright P. E. Conformational propensities of intrinsically disordered proteins influence the mechanism of binding and folding. Proc. Natl. Acad. Sci. U.S.A. 2015, 112, 9614–9619. 10.1073/pnas.1512799112. 26195786 PMC4534220

