# Supplementary material for: Competitive Binding of Viral Nuclear Localization Signal Peptide and Inhibitor Ligands to Importin-α Nuclear Transport Protein
Source: J Chem Inf Model. 2024 Jun 13;64(13):5262–72. doi: 10.1021/acs.jcim.4c00626 (PMC11234363; doi:10.1021/acs.jcim.4c00626)
Supplement: Supplementary file 1 — ci4c00626_si_001.pdf [file ci4c00626_si_001.pdf]

Supporting Information 1

Competitive Binding of Viral Nuclear Localization Signal Peptide and Inhibitor Ligands to Importin-α Nuclear Transport Protein

Bryan M. Delfing[1], Xavier E. Laracuente[1], William Jeffries[1], Xingyu Luo[1], Audrey Olson[1], Kenneth W. Foreman[2], Greg Petruncio[2,3], Kyung Hyeon Lee[2,3], Mikell Paige[2,3], Kylene Kehn-Hall[4,5], Christopher Lockhart[1], and Dmitri K. Klimov[1*]

[1]School of Systems Biology, George Mason University, Manassas, VA 20110, USA
[2]Department of Chemistry and Biochemistry, George Mason University, Manassas, VA 20110, USA
[3]Center for Molecular Engineering, George Mason University, Manassas, VA, 20110
[4] Department of Biomedical Sciences and Pathobiology, Virginia-Maryland College of Veterinary Medicine, Virginia Polytechnic Institute and State University, Blacksburg, VA 24061, USA
[5]Center for Emerging, Zoonotic, and Arthropod-borne Pathogens, Virginia Polytechnic Institute and State University, Blacksburg, VA 24061, USA
*E-mail: dklimov@gmu.edu

REST algorithm performance: To explore the competitive binding (CB) of the coreNLS (KKPKKE) peptide and inhibitor I1 or I2 to importin-α (impα) we used replica exchange with solute tempering (REST) molecular dynamics. The REST simulations utilized R = 10 replicas with the temperatures distributed geometrically from T = 310 K to 510 K (see Methods). The REST technical performance has been tested in several ways. First, replicas are expected to randomly walk across the temperature range. This behavior is visualized in Fig. S1, where replica visits to different temperatures create a random mosaic, indicating their random exchanges between temperatures without prolonged trappings.

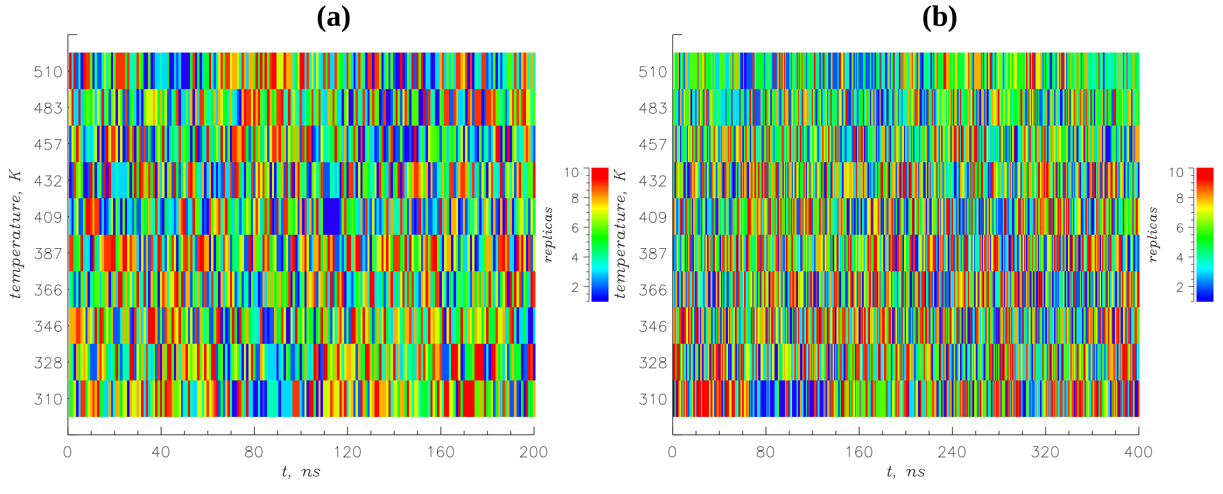

Figure S1. Walks of REST replicas across temperatures in one of the trajectories probing competitive binding of the coreNLS peptide and I1 (a) or I2 (b) inhibitor to impα. The color scales show the initial assignment of replicas to temperatures. Other REST trajectories exhibited similar behavior.

Second, to quantify the distribution of replicas across temperatures we resort to the replica mixing parameter [1],

$$m(T) = 1 - \frac{\sqrt{\sum_{r=0}^{R-1} t_r^2}}{\sum_{r=0}^{R-1} t_r}, \qquad \text{(S1)}$$

where T is the REST temperature and t_r is the time spent by replica r at T. If R = 10 replicas are randomly mixed, then $m(T)$ reaches the theoretical maximum $m_r = 1 - 1/R^{1/2} = 0.68$. Fig. S2 demonstrates that $m(T)$ approaches m_r in both CB simulations with I1 or I2. Indeed, the average $m(T)$ across all temperatures are 0.68 and 0.66 for the CB with I1 and I2, respectively. Third, we computed the replica exchange rates $a(T)$ presented in Fig. S3. Although $a(T)$ gradually increases with T, the average a is 0.34 for I1 and 0.33 for I2, which are close to the optimum range [2]. Taken together, Figs. S1-S3 suggest that replicas are well-mixed across temperatures, as prescribed by REST formalism.

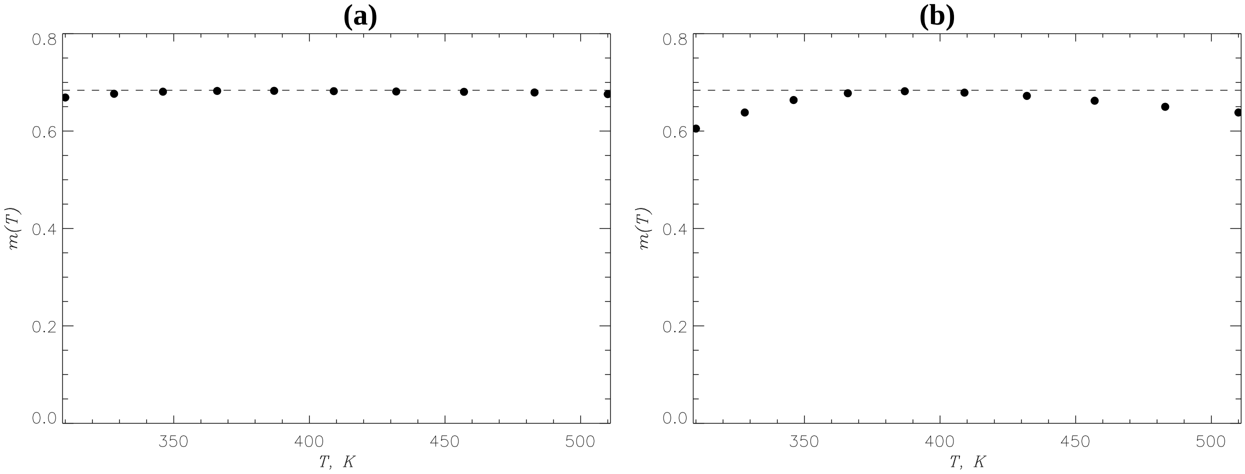

Figure S2. The replica mixing parameter $m(T)$ for the competitive binding simulations with I1 (a) and I2 (b) inhibitors is plotted as a function of REST temperature T (solid circles). The maximum theoretical value $m_r = 0.68$ is marked by a dashed line. The data are averaged across four REST trajectories. Standard errors are too small to show.

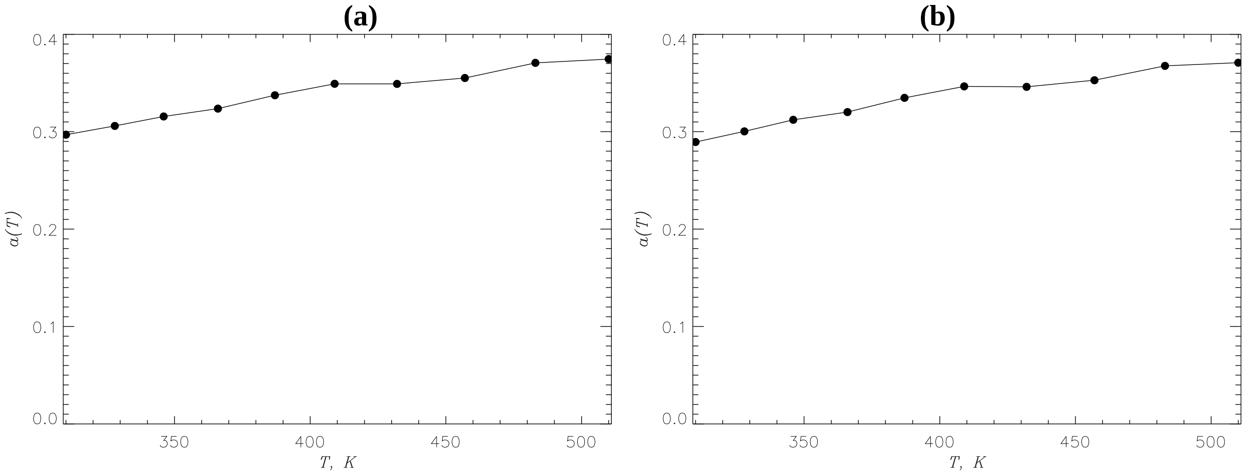

Figure S3. Replica exchange rates, $a(T)$, are plotted vs REST temperatures T for the competitive binding simulations with I1 (a) and I2 (b) inhibitors. The data are averaged across four REST trajectories. Standard errors are too small to show.

Convergence of REST sampling: Because CB involves simultaneous interactions between coreNLS peptide, inhibitor, and impα protein, it is critical to establish that the REST sampling of their interactions has converged. To evaluate peptide binding to impα, we plot in Fig. S4a,b the numbers of contacts $C(t)$

forming between coreNLS and impα amino acids in the course of REST simulations (see Models and Methods). It is seen that in the last 140 ns for I1 or 170 ns for I2 $C(t)$ follow approximate baselines. To probe inhibitor binding to the peptide, Fig. S4c,d presents the numbers of contacts $C_{i\text{-}p}(t)$ occurring between inhibitor groups and the peptide amino acids. For both inhibitors this quantity appears well equilibrated over all sampling times. Finally, to examine the interactions between inhibitor and impα, we consider the number of contacts $C_{i\text{-}imp\alpha}(t)$ between inhibitor groups and impα amino acids. It follows from Fig. S4e,f that the inhibitor-impα interactions require no equilibration for I1 or I2.

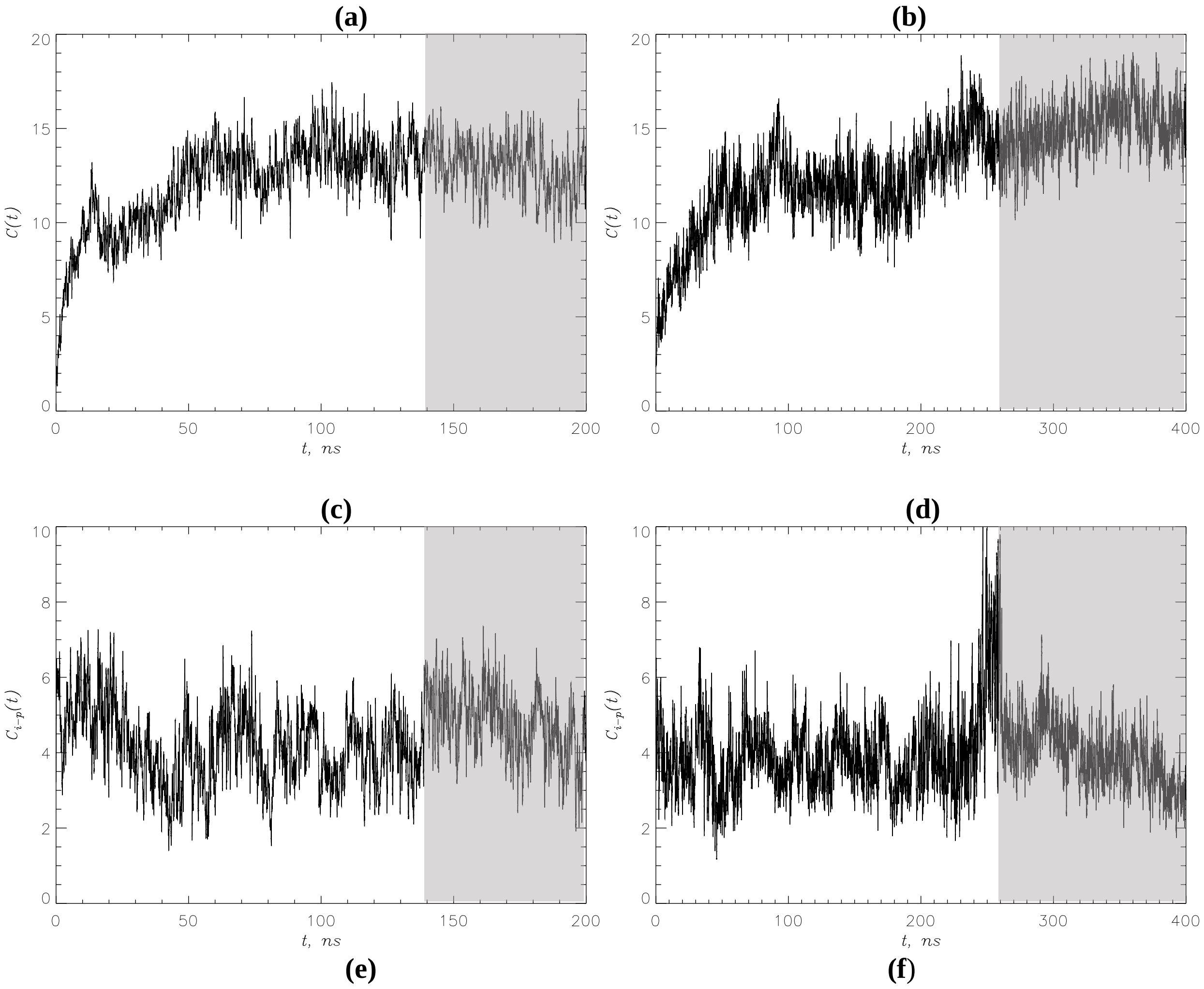

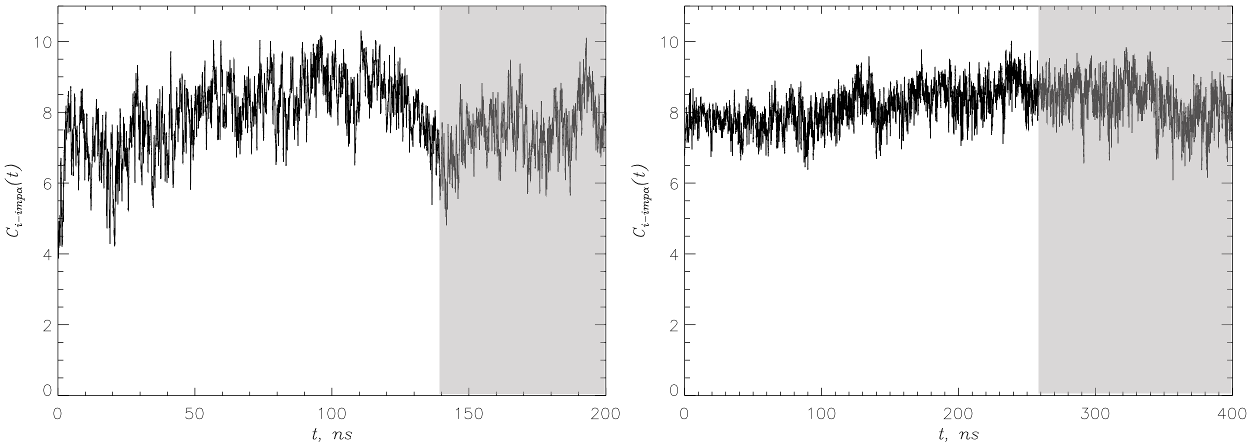

Figure S4. (a,b) The numbers of contacts $C(t)$ forming between coreNLS and impα amino acids are plotted as a function of REST sampling time t. (c,d) The numbers of contacts $C_{i\text{-}p}(t)$ occurring between an inhibitor and the peptide amino acids are plotted as a function of REST sampling time t. (e,f) The numbers of contacts $C_{i\text{-}imp\alpha}(t)$ observed between an inhibitor and impα amino acids are plotted as a function of REST sampling time t. Data in (a,c,e) and (b,d,f) refer to I1 and I2 CB, respectively. The quantities are averaged over four REST trajectories and collected at 310 K. The data are smoothed with the sliding window of 200 ps. Shaded regions show the equilibrated data collected after equilibration times t_{eq}, which are 140 ns for I1 and 260 ns for I2 CB. These equilibration times are established combining the analysis of this figure and Fig. S5.

Although the quantities plotted in Fig. S4 suggest REST convergence, they report solely the total numbers of interactions. To provide a more nuanced test of REST convergence, we used the approach based on evaluating individual contacts [3]. To this end, we first computed the contact maps $c(i,j;t,n)$ between impα amino acid i and peptide amino acid j at time t in a REST trajectory n. The contact maps $c(i,j;t,n)$ report native or non-native contacts as 1s or 0s otherwise. Then, the REST timeline was divided into ten (for I1) or twenty (for I2) 20 ns windows k, and $c(i,j;t,n)$ for a given system were averaged within a given k resulting in $C(i,j;k,n)$. Using $C(i,j;k,n)$ we computed the contact root mean squared deviation $cRMSD(k)$

$$cRMSD\,(k) = \left[\frac{1}{N_{tr}}\sum_n \frac{1}{N_c}\sum_{i,j}\left(C(i,j;k,n) - c^{ref}(i,j)\right)^2\right]^{\frac{1}{2}}, \qquad \text{(S2)}$$

where $c^{ref}(i,j)$ represents the contact map computed for the initial structure in a REST trajectory at 310 K, N_{tr} is the number of REST trajectories, N_c is the total number of possible peptide-impα contacts, and k=0, …, 9 for I1 or 19 for I2. Similar contact root mean squared deviations $cRMSD_{i\text{-}p}(k)$ and $cRMSD_{i\text{-}imp\alpha}(k)$ were computed for the contact maps describing inhibitor-peptide and inhibitor-impα interactions. Fig. S5 shows the respective contact root mean squared deviations for I1 and I2 CB. For I1 CB, consistent with Fig. S4 analysis, $cRMSD_{i\text{-}imp\alpha}(k)$ does not reveal ongoing equilibration process, whereas $cRMSD(k)$ and $cRMSD_{i\text{-}p}(k)$ suggest equilibration after approximately 60 or 140 ns of sampling. For I2 CB $cRMSD(k)$ and $cRMSD_{i\text{-}imp\alpha}(k)$ indicate that REST equilibration is completed after about 260 or 180 ns. $cRMSD_{i\text{-}p}(k)$ does not reveal equilibration. Thus, summarizing the above analysis we conclude that the latest equilibration times t_{eq} for the REST sampling of I1 and I2 CB, which provide the equilibration of all quantities in Figs. S4,5, are 140 and 260 ns, respectively.

(a) **(b)**

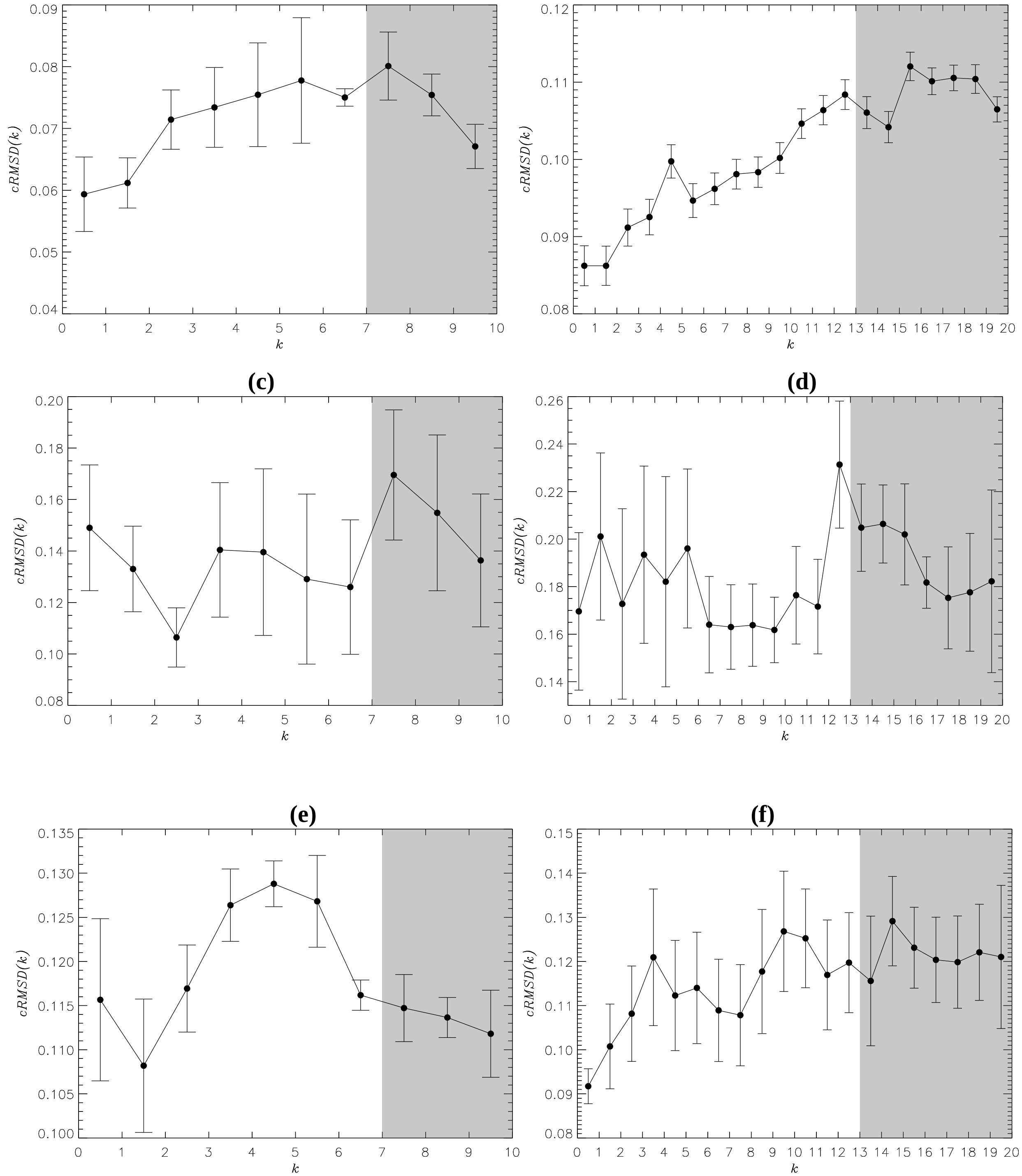

Figure S5. The contact root mean squared deviations $cRMSD(k)$ computed as a function of time windows k for the CB of the coreNLS peptide with I1 (a) and I2 (b). The contact root mean squared deviations $cRMSD_{i\text{-}p}(k)$ and $cRMSD_{i\text{-}imp\alpha}(k)$ are presented in (c,e) for I1 and (d,f) for I2 CB. Shaded regions show the equilibrated data collected after equilibration times t_{eq}, which are 140 ns for I1 and 260 ns for I2 CB.

Sampling of native coreNLS binding poses: CB simulations were initialized with the structures resulting after quenching from 700 K to the respective REST temperatures. As a result, they typically have a low native content measured by the fraction of retained native contacts $P_n(j)$. To ascertain that the abrogation of native

binding by the coreNLS peptide is not the consequence of initial conditions, we monitored the root mean squared deviations (RMSD) of the peptide poses from the PDB structure 3VE6 in the course of REST simulations at 310K. Fig. S6a,b presents the corresponding RMSD as a function of REST time *t* for I1 CB simulation and compares it with the analogous plot obtained from non-competitive binding (NCB) simulations of the coreNLS peptide [3]. Due to a single dominant native cluster capturing 67% of peptide poses in NCB [3], the coreNLS consistently samples the RMSD values below 3 Å. Although in CB the peptide samples, as a rule, much higher RMSDs, it still visits the poses with the RMSD as low as 3 Å. To verify that these low RMSD poses are similar to those sampled in NCB, we plotted the RMSD between the centroid of the native NCB cluster [3] and the poses observed in CB. Since the respective RMSD in Fig. S6c drops occasionally to as low as 2 Å, these CB poses are indeed very similar to the native NCB poses. This analysis argues that, although the coreNLS peptide in CB visits the native binding pose, it does not constitute a thermodynamically stable bound state. Consequently, the peptide rapidly escapes to the structures with low native content. Similar behavior was observed for I2 CB. Therefore, abrogation of a native binding pose by the coreNLS peptide is a genuine effect caused by inhibitor action.

(a)

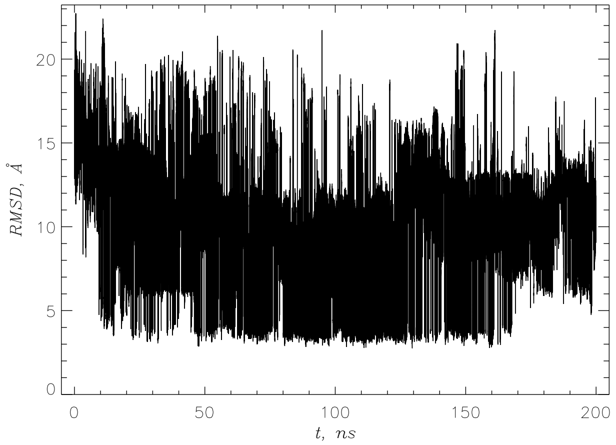

(b)

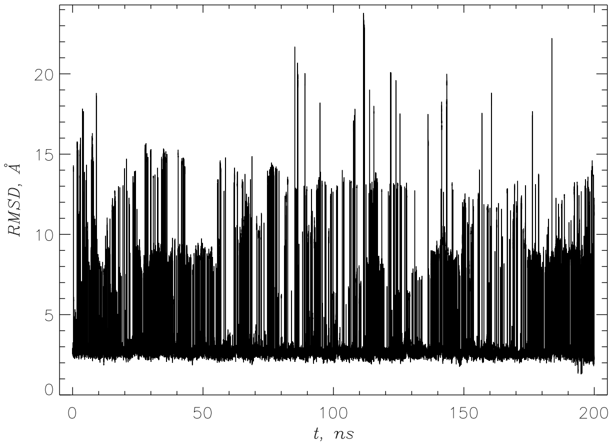

(c)

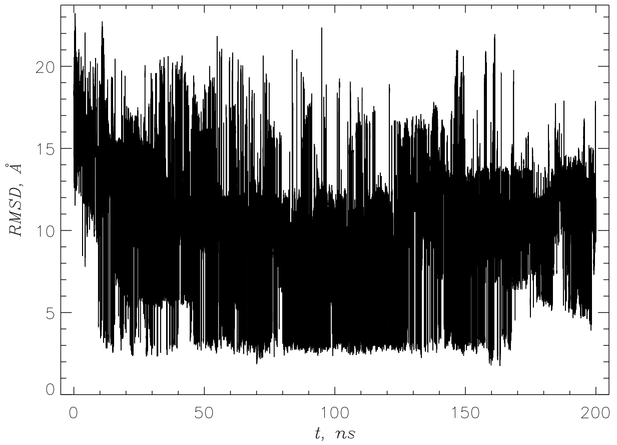

Figure S6. (a,b) The root mean squared deviations *RMSD(t)* of the coreNLS peptide poses from the PDB structure 3VE6 as a function of REST time *t* in CB (a) and NCB [3] (b) simulations at 310K. (c) *RMSD*(*t*) computed between the centroid of the native NCB cluster [3] and the poses observed in CB at 310K. The data in the figure are shown for I1 inhibitor in representative trajectories.

Inhibitor binding to importin-α: To determine the locations of inhibitor binding to impα, we computed the probabilities $P_{i,p}(i)$ of forming contacts between impα amino acids i and an inhibitor. The ten impα amino acids with the largest $P_{i,p}(i)$ observed in CB and, for reference, in NCB are listed in Table S1.

Table S1 Top impα amino acids with the strongest affinities toward inhibitors.

rank	amino acid *i*	$P_{i,p}(i)$	amino acid *i*	$P_{i,p}(i)$	amino acid *i*	$P_{i,p}(i)$	amino acid *i*	$P_{i,p}(i)$
	**Inhibitor I1**				**Inhibitor I2**			
	**Non-competitive binding** [a]		**Competitive binding**		**Non-competitive binding** [a]		**Competitive binding**	
**1**	Ser79	0.71	Ser79	0.60	Ser79	0.80	Trp114	0.83
**2**	Trp114	0.57	Asn76	0.50	Asn76	0.77	Trp72	0.74
**3**	Asn76	0.55	Trp114	0.47	Pro40	0.70	Trp161	0.66
**4**	Ile42	0.43	Ser35	0.41	Ile42	0.66	Gln111	0.59
**5**	Trp161	0.42	Trp161	0.40	Gly80	0.65	Arg158	0.57
**6**	Pro40	0.42	Trp72	0.38	Leu34	0.63	Ser79	0.49
**7**	Asn118	0.41	Pro40	0.37	Thr81	0.53	Asn76	0.48
**8**	Trp72	0.41	Ile42	0.37	Gln39	0.51	Arg157	0.45
**9**	Ser35	0.41	Leu34	0.36	Arg36	0.51	Gly154	0.41
**10**	Gly80	0.40	Asn118	0.34	Gln37	0.48	Asn118	0.30

[a] Data from [4]

Conformational ensemble of coreNLS peptide: It is of interest to evaluate the effect of inhibitors on the coreNLS conformational ensembles. To this end, we computed the probability distributions $P(R_g)$ of the radius of gyration R_g of coreNLS peptide observed in CB and NCB. Fig. S7 presents the resulting plots for both inhibitors. The implications of this figure are discussed in the main text.

(a) **(b)**

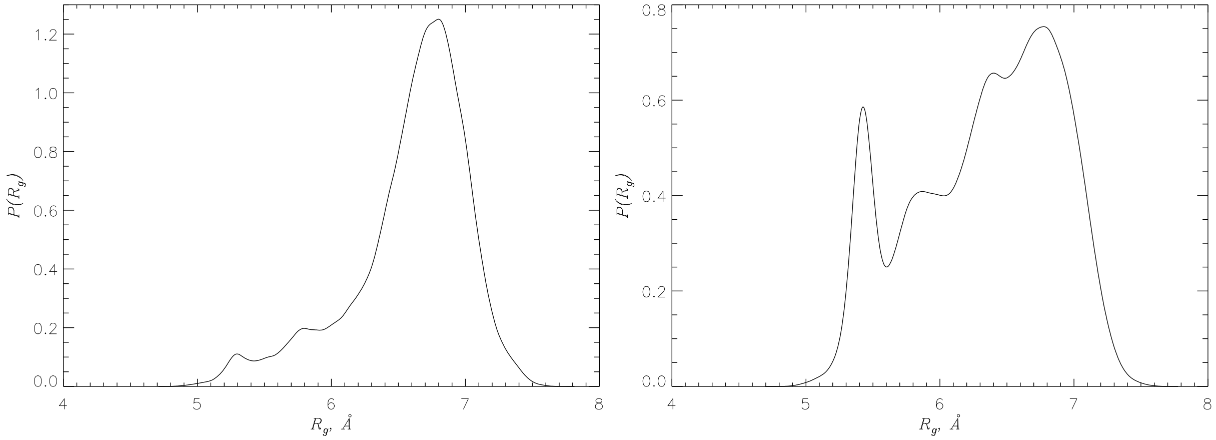

(c)

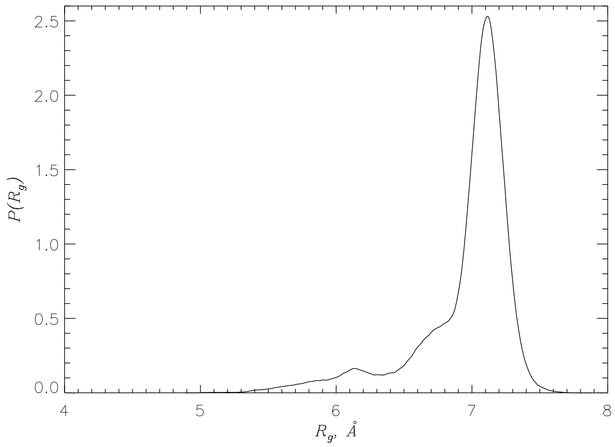

Figure S7 The probability distributions $P(R_g)$ of the radius of gyration R_g of coreNLS peptide observed in CB with I1 (a) or I2 (b) and in NCB (c).

Binding free energy: To compute the change in the free energy of binding of the coreNLS peptide to impα $\Delta\Delta G_b(x)$ caused by the inhibitor *x*, we applied the MM-GBSA approach outlined in Model and Methods. Table S2 lists the free energies and their components defined in Eqs. (1,2) in the main text for the coreNLS peptide, impα, impα+NLS complex [3], impα-*x* complex [4], and impα+NLS-*x* complex. Note that for the last two systems the potential and solvation energies of *x* are excluded but the interaction of *x* with impα and/or the coreNLS is included. Table S3 presents the binding free energy ΔG_b(NCB) for NCB, binding free energies ΔG_b(CB;*x*) for CB with the inhibitor *x*, and the changes in binding free energies caused by inhibitors $\Delta\Delta G_b$(*x*). In addition, Table S4 presents the contributions to $\Delta\Delta G_b$(*x*) from the molecular mechanical energy E_{mm}, polar and apolar solvation energies $G_{solv,p}$ and $G_{solv,ap}$, and entropy *TS* listed in Eq. (1).

Table S2 Free energies and their components for various systems [a]

System	E_{mm}, kcal/mol	$G_{solv,p}$, kcal/mol	$G_{solv,ap}$, kcal/mol	*TS*, kcal/mol	*G*, kcal/mol
**coreNLS peptide**	1492.3	-1697.7	6.2	2.2	-201.1
**Impα**	643.6	-2526.8	54.7	3.1	-1831.5
**Impα+coreNLS[b]**	389.5	-2513.7	54.5	3.3	-2073.0
**Impα-I1[c]**	625.2	-2528.6	53.7	3.2	-1852.9
**Impα-I2[c]**	693.4	-2588.0	53.1	3.2	-1844.7
**Impα+NLS-I1**	519.5	-2647.3	56.1	3.2	-2075.0
**Impα+NLS-I2**	513.0	-2643.2	56.0	3.4	-2077.6

[a] “minus” in the system name indicates that the contribution from the inhibitor is not counted, while the interaction between the inhibitor and the rest of the complex is included.
[b] data collected in [3].
[c] data collected in [4].

Table S3 Free energies of binding and their changes due to inhibitor interference.

**quantity**	**Free energy,** kcal/mol	
ΔG_b**(NCB)**	-40.3	
	**Inhibitor** x**=I1**	**Inhibitor** x**=I2**
$\Delta G_b(CB;x)$	-21.0	-31.8
$\Delta\Delta G_b(x)$	19.4	8.5

Table S4 Contributions to the changes in the free energy of coreNLS binding to impα

**quantity**	**Inhibitor I1**	**Inhibitor I2**
$\Delta\Delta E_{mm}$, kcal/mol	148.5	73.8
$\Delta\Delta G_{solv,p}$, kcal/mol	-131.8	-68.3
$\Delta\Delta G_{solv,ap}$, kcal/mol	2.6	3.1
$\Delta\Delta TS$, kcal/mol	-0.1	0.1

It is important to discuss the limitations of computing $\Delta\Delta G_b(x)$ in Table S3. In our REST simulations impα was restrained with soft harmonic potentials to the 3VE6 fold. Previous studies have shown that impα structure without N-terminal self-inhibitory domain experiences bending and twisting motions [5]. These motions may interfere with the native bound pose of the coreNLS peptide. Consequently, one may anticipate that such motions reduce the binding free energy ΔG_b(NCB), because the peptide must overcome additional free energy loss to adopt specific binding pose. This correction is unlikely to apply to the competitive binding because the peptide does not intercalate into impα. Therefore, both values of $\Delta\Delta G_b(x)$ in Table S3 are likely to be overestimated. This correction, however, does not affect our conclusion that the inhibitor I1 destabilizes the binding of the coreNLS peptide to impα stronger than I2 does.

References

[1] Han, M. and Hansmann, U. H. E. (2011) Replica exchange molecular dynamics of the thermodynamics of fibril growth of Alzheimer’s Aβ42 peptide. *J. Chem. Phys.* **135**, 065101.
[2] Denschlag, R., Lingenheil, M., and Tavan, P. (2009) Optimal temperature ladders in replica exchange simulations. *Chem. Phys. Lett.* **473**, 193-195.
[3] Delfing, B. M., Laracuente, X. E., Olson, A., Foreman, K. W., Paige, M., Kehn-Hall, K., Lockhart, C., and Klimov, D. K. (2023) Binding of Viral Nuclear Localization Signal Peptides to Importin-*α* Nuclear Transport Protein. *Biophys. J.* **122**, 3476-3488.
[4] Delfing, B. M., Olson, A., Laracuente, X., Foreman, K. W., Paige, M., Kehn-Hall, K., Lockhart, C., and Klimov, D. K. (2023) Binding of Venezuelan Equine Encephalitis Virus Inhibitors to Importin-α Receptors Explored with All-Atom Replica Exchange Molecular Dynamics. *J. Phys. Chem.* B **127**, 3175–3186.
[5] Geraldo, M. T., Takeda, A. A. S., Braz, A. S. K., and Lemke, N. (2016) Bending-Twisting Motions and Main Interactions in Nucleoplasmin Nuclear Import. PLoS ONE **11**, e0157162.
