# Supplementary material for: Competitive Binding of Viral Nuclear Localization Signal Peptide and Inhibitor Ligands to Importin-α Nuclear Transport Protein
Source: J Chem Inf Model. 2024 Jun 13;64(13):5262–72. doi: 10.1021/acs.jcim.4c00626 (PMC11234363; doi:10.1021/acs.jcim.4c00626)
Supplement: Supplementary file 2 — ci4c00626_si_002.pdf [file ci4c00626_si_002.pdf]

Supporting Information 2

Competitive Binding of Viral Nuclear Localization Signal Peptide and Inhibitor Ligands to Importin-α Nuclear Transport Protein

Bryan M. Delfing[1], Xavier E. Laracuente[1], William Jeffries[1], Xingyu Luo[1], Audrey Olson[1], Kenneth W. Foreman[2], Greg Petruncio[2,3], Kyung Hyeon Lee[2,3], Mikell Paige[2,3], Kylene Kehn-Hall[4,5], Christopher Lockhart[1], and Dmitri K. Klimov[1*]

[1]School of Systems Biology, George Mason University, Manassas, VA 20110, USA
[2]Department of Chemistry and Biochemistry, George Mason University, Manassas, VA 20110, USA
[3]Center for Molecular Engineering, George Mason University, Manassas, VA, 20110
[4] Department of Biomedical Sciences and Pathobiology, Virginia-Maryland College of Veterinary Medicine, Virginia Polytechnic Institute and State University, Blacksburg, VA 24061, USA
[5]Center for Emerging, Zoonotic, and Arthropod-borne Pathogens, Virginia Polytechnic Institute and State University, Blacksburg, VA 24061, USA
*E-mail: dklimov@gmu.edu

COMPOUND SYNTHESIS PROCEDURES

General: Special instructions regarding reagents are noted in the appropriate procedures where they are employed. “Drying an organic layer” refers to treatment with Na_2SO_4 or $MgSO_4$ after aqueous workup and then decanting. “Flame-drying” refers to heating glassware with a propane torch for one minute and allowed to cool prior to use. “Flushing/purging a reaction vessel” refers to delivering an inert gas using balloon+syringe+needle system through a septum for several minutes. A Biotage® Initiator was used for microwave reactions. Reaction progress was monitored on SiliCycle F^{254} TLC plates. “TLCMS” refers to MS analysis of spots on a TLC plate. “Concentrated” refers to removal of volatile solvent using Biotage® V10 Touch or Buchi rotary evaporator with water bath temperature between 40-50 °C. Products were purified using SiliCycle SiliaFlash® F60 silica gel (40-63 µm, 230-400 mesh) with solvent mixtures specified in the corresponding experiment.
Instrumentation: APCI-MS was performed on an Advion Expression CMS. TLCMS was performed on Advion Expression CMS Plate Express attachment, which allows for direct APCI MS analysis of spots on a TLC plate. ^{1}H and ^{13}C NMR spectra were obtained on a Bruker 400 MHz spectrometer equipped with a PA BBO probe and spectra were calibrated using TMS or the solvent residual peak. LCMS and UV-trace analysis was run on a SciEx QTRAP 4100.

Overall Scheme: Synthesis of I1 (aka 1564) and I2

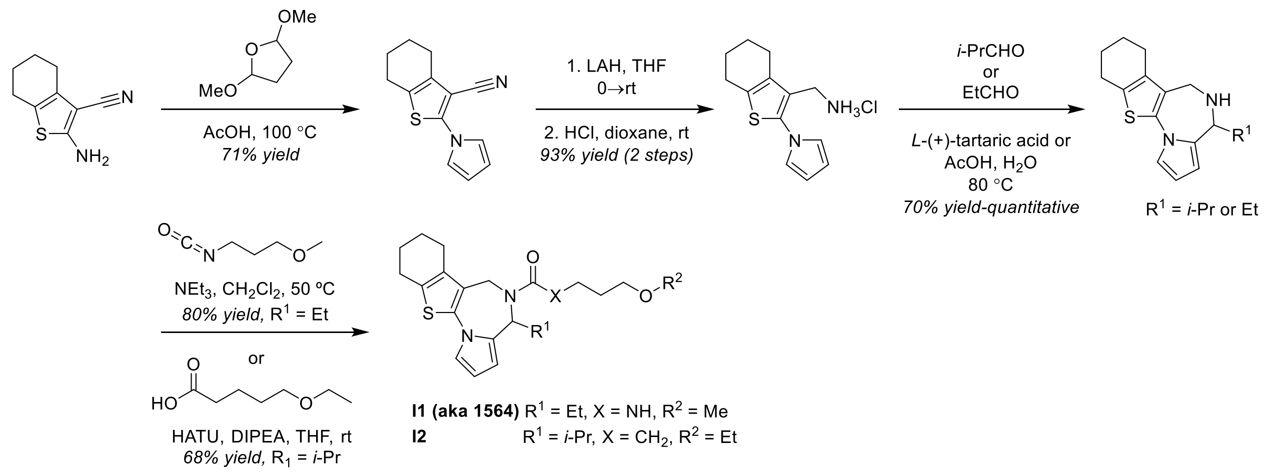

Step 1: Microwave-promoted Paal-Knorr Pyrrole Reaction (Synthesis of 2-(1H-Pyrrol-1-yl)-4,5,6,7-tetrahydrobenzo[b]thiopthene-3-carbonitrile)

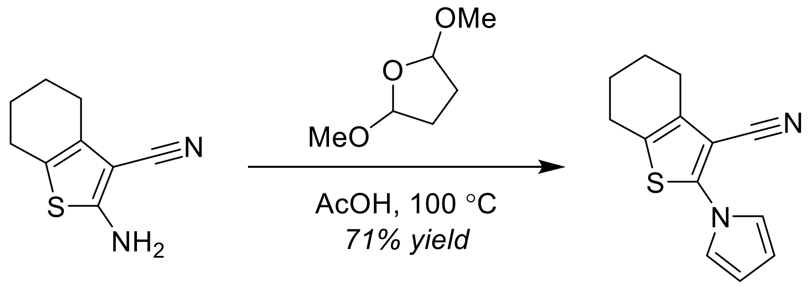

Procedure Dimethoxytetrahydrofuran (DMTHF) (7.4 mL, 56.10 mmol, 5 equiv.), thiophene-3-carbonitrile (2000 mg, 11.22 mmol, 1 equiv.), and glacial acetic (12 mL) were added to a microwave vial, sealed, and heated at 100°C for 1 hour. After TLC indicated reaction completion, the reaction was diluted with EtOAc and then washed with water 3 times. The organic layer was dried and concentrated to remove residual AcOH and DMTHF to obtain a crude brown solid. The solid was recrystallized from 1:1 *i*-PrOH-water to obtain 1828 mg light orange crystals (71% yield). Alternatively, the product can be purified by column chromatography using 1:19 EtOAc-Hex.

TLC system: 1:9 EtOAc-Hex (*From left to right: sm = starting material, rxn = reaction, co = cospot*)

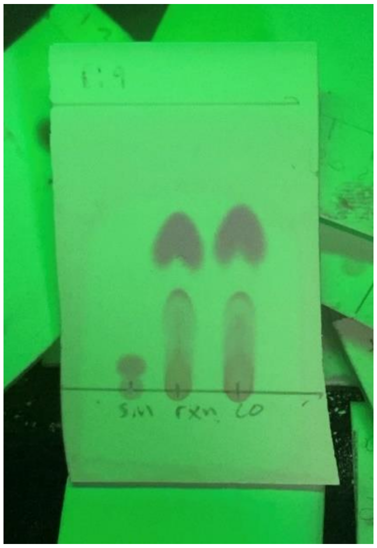

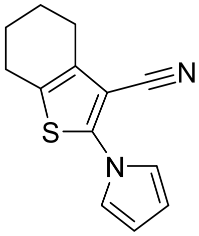

2-(1H-Pyrrol-1-yl)-4,5,6,7-tetrahydrobenzo[b]thiopthene-3-carbonitrile

^{1}H NMR (400 MHz, $CDCl_3$) δ 1.83–1.89 (m, 4H, $2CH_2$), 2.65–2.66 (m, 4H, $2CH_2$), 6.35–6.36(m, 2H, ArH), 7.14–7.26 (m, 2H, ArH) ppm.

^{13}C NMR (125 MHz, $CDCl_3$) δ 21.9, 23.0, 23.3, 24.4, 98.5, 111.8, 114.4, 121.2, 129.7, 134.9, 149.2.

APCI-MS $[M+1]^{+}$: 228.9

Step 2: Nitrile Reduction and Amine Salt Formation (Synthesis of [2-(1H-pyrrol-1-yl)-4,5,6,7-tetrahydro-1-benzothiophen-3-yl]methanaminium chloride)

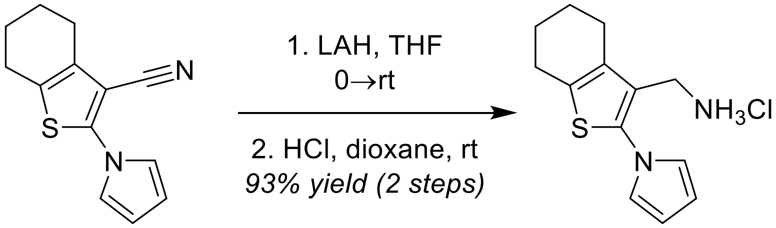

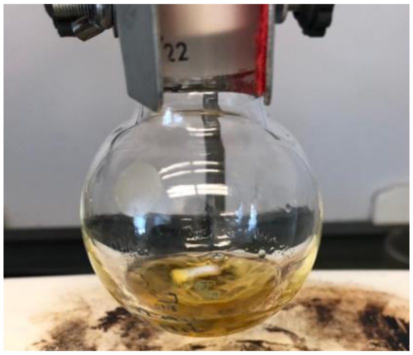

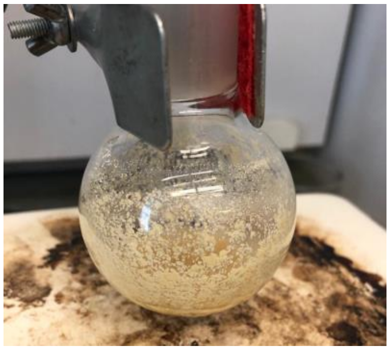

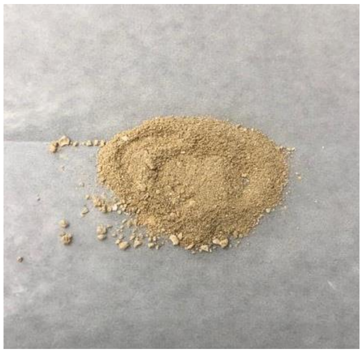

Procedure LAH was titrated with I_2 before use. 2-(1H-Pyrrol-1-yl)-4,5,6,7-tetrahydrobenzo[b]thiopthene-3-carbonitrile (1545 mg, 6.77 mmol, 1 equiv.) was dissolved in anhydrous THF (30 mL) and cooled to 0 °C under nitrogen. LAH in THF (30 mL, 0.46 M, 2 equiv.) was added dropwise using a syringe pump. After addition was complete, the reaction was allowed to slowly warm up to room temperature over 1.5 hours. After TLC indicated reaction completion, the reaction was cooled back down to 0 °C and quenched with 1 M NaOH (aq.) dropwise, which formed a white precipitate. When the reaction stopped fizzing upon NaOH addition, it was filtered through celite and the crude product was eluted with 200 mL Et_2O. The filtrate was concentrated down to ~50 mL Et_2O. To the ether solution was added approximately 3-4 mL of 4 M HCl in dioxane dropwise, which caused precipitation of crystals. The crystals were filtered and washed with cold Et_2O. Obtained 1688 mg of light brown solid (93% yield over 2 steps).

TLC system: 1:3 EtOAc-Hex (*From left to right: sm = starting material, rxn = reaction, co = cospot*)

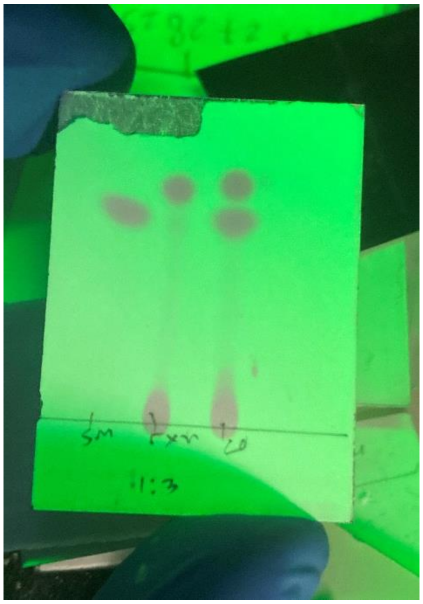

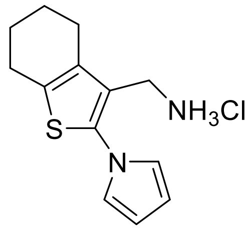

[2-(1H-pyrrol-1-yl)-4,5,6,7-tetrahydro-1-benzothiophen-3-yl]methanaminium chloride

APCI-MS $[M-NH_3]^+$: 215.9

Step 3: Pictet-Spengler Cyclization (Synthesis of 7-(propan-2-yl)-17-thia-2,8-diazatetracyclo[8.7.0.$0^{2,6}$.$0^{11,16}$]heptadeca-1(10),3,5,11(16)-tetraene or 7-ethyl-17-thia-2,8-diazatetracyclo[8.7.0.$0^{2,6}$.$0^{11,16}$]heptadeca-1(10),3,5,11(16)-tetraene)

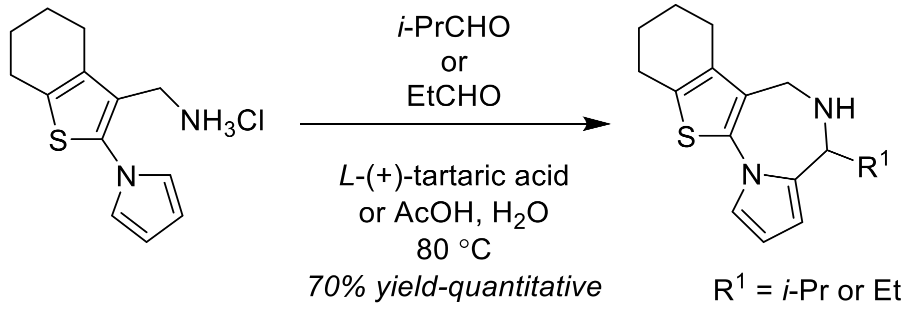

Procedure [2-(1H-pyrrol-1-yl)-4,5,6,7-tetrahydro-1-benzothiophen-3-yl]methanaminium chloride (1236 mg, 4.6 mmol, 1 equiv.) was added to H_2O (50 mL) and was partially soluble. AcOH (0.8 mL, 13.8 mmol, 3 equiv.) and isobutyraldehyde (0.67 mL, 7.36 mmol, 1.6 equiv.) were added and the reaction was heated in 80 ˚C oil bath overnight (~18 hours). After TLC indicated reaction completion, diluted the reaction with DCM and then washed with water twice, and then once with brine. The organic layer was dried and concentrated down to 1748 mg brown solid (quantitative yield), which can be used in the final step without further purification. Alternatively, the product can be purified via column chromatography using a system of 2.5-5% MeOH, 1% NH_4OH, 94-96.5% DCM to give a yield of approximately 70%.

TLC system: 1:9 MeOH-DCM + 1% NH_4OH (*From left to right: sm = starting material, rxn = reaction, co = cospot*)

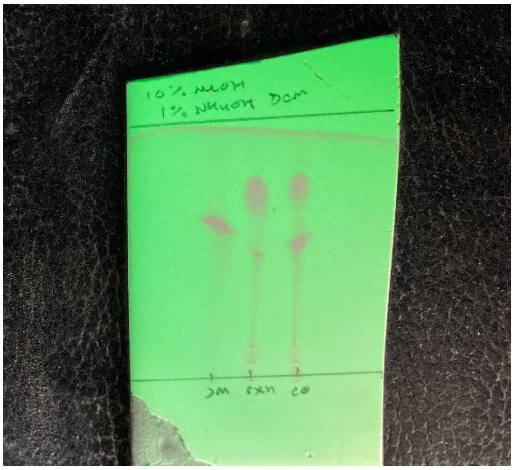

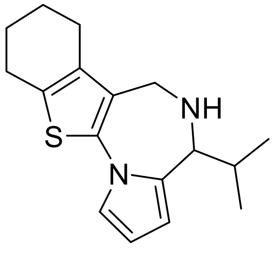

7-(propan-2-yl)-17-thia-2,8-diazatetracyclo[8.7.0.$0^{2,6}$.$0^{11,16}$]heptadeca-1(10),3,5,11(16)-tetraene

^{1}H NMR (400 MHz, $CDCl_3$) δ 1.01-1.10 (d, 6H, CH_3), 1.81–1.83 (m, 4H, 2CH_2), 2.32-2.36 (m, 2H, CH_2), 2.67-2.69 (m, 2H, CH_2), 3.48-3.49 (m, 1H, NH), 3.87-4.00 (dd, 2H, CH_2), 6.01 (dd, 1H, ArH), 6.14 (dd, 1H, ArH), 6.92 (dd, 1H, ArH) ppm.

^{13}C NMR (125 MHz, $CDCl_3$) δ 18.8, 21.1, 22.2, 22.3, 23.1, 24.0, 24.6, 29.2, 29.8, 43.1, 59.1, 62.6, 109.1, 109.5, 121.6, 124.3, 129.6, 132.6, 133.3, 135.7, 176.8.

APCI-MS $[M+1]^+$: 287.0

Procedure The following reaction conditions were adopted by Byeon et al.[1] [2-(1H-pyrrol-1-yl)-4,5,6,7-tetrahydro-1-benzothiophen-3-yl]methanaminium chloride (400 mg, 1.48 mmol, 1 equiv.) was added to H_2O (15 mL) and was partially soluble. *L*-(+)-Tartaric acid (111 mg, 0.74 mmol, 0.5 equiv.) and propionaldehyde (169 μL, 2.36 mmol, 1.6 equiv.) were added and the reaction was heated to 70 ˚C in the microwave for 12 hours. After TLC indicated reaction completion, the reaction was neutralized to pH 7 using saturated NaOH (aq.), which formed a precipitate. The product was extracted with 3 DCM washes. The organic layers, were combined, dried, and concentrated down to crude which was then purified via column chromatography using a system of 2.5-5% MeOH, 1% NH_4OH, 94-96.5% DCM. Obtained 287 mg yellow solid (71% yield).

TLC system: 1:9 MeOH-DCM + 1% NH_4OH (*From left to right: sm = starting material, rxn = reaction, co = cospot*)

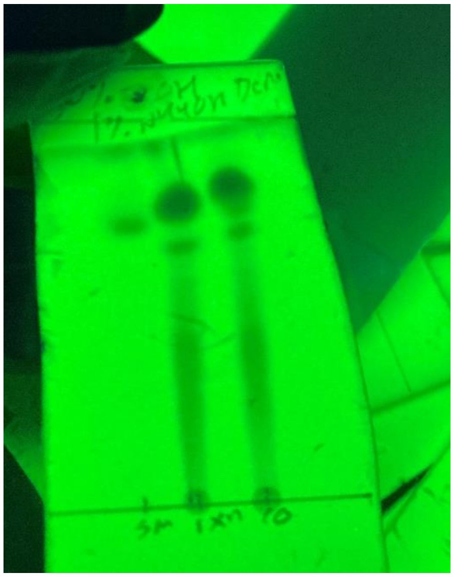

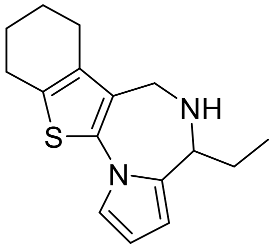

7-ethyl-17-thia-2,8-diazatetracyclo[8.7.0.0^{2,6}.0^{11,16}]heptadeca-1(10),3,5,11(16)-tetraene

^{1}H NMR (400 MHz, $CDCl_3$) δ 1.06-1.10 (t, 3H, J = 7.36 Hz), 1.73–1.85 (m, 4H), 1.95-2.00 (m, 2H), 2.37-2.39 (m, 2H), 2.67-2.70 (m, 2H), 3.57-3.60 (dd, 1H, J = 7.52 Hz, 6.16 Hz), 3.92-3.93 (d, 2H, J = 3.2 Hz), 6.01-6.02 (m, 1H), 6.15-6.16 (t, 1H, J = 3.28 Hz), 6.91-6.92 (dd, 1H, J = 2.84 Hz, 1.72 Hz).

^{13}C NMR (125 MHz, $CDCl_3$) δ 11.34, 22.34, 23.17, 24.28, 24.51, 26.42, 47.40, 55.88, 105.73, 108.86, 120.88, 126.45, 128.07, 133.29, 134.26, 138.35

APCI-MS $[M+1]^+$: 273.0

Step 4: Isocyanate Addition or Amidation (Synthesis of 5-ethoxy-1-[7-(propan-2-yl)-17-thia-2,8-diazatetracyclo[8.7.0.0^{2,6}.0^{11,16}]heptadeca-1(10),3,5,11(16)-tetraen-8-yl]pentan-1-one or 7-ethyl-N-(3-methoxypropyl)-17-thia-2,8-diazatetracyclo[8.7.0.0^{2,6}.0^{11,16}]heptadeca-1(10),3,5,11(16)-tetraene-8-carboxamide)

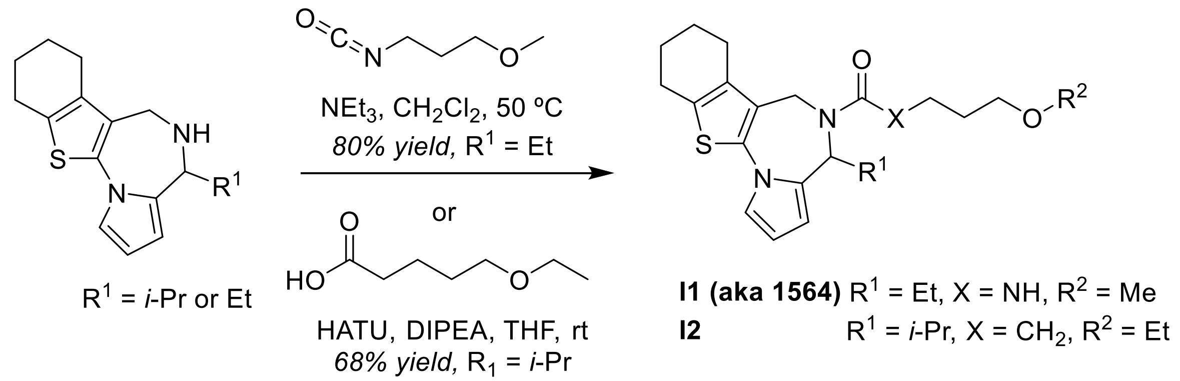

Procedure 7-ethyl-17-thia-2,8-diazatetracyclo[8.7.0.0^{2,6}.0^{11,16}]heptadeca-1(10),3,5,11(16)-tetraene (37 mg, 0.136 mmol, 1 equiv.) and 1-isocyanato-3-methoxypropane (46 mg, 0.39 mmol, 2.9 equiv.) were added to a flame dried microwave vial. These were then dissolved in anhydrous DCM (3 mL) and to this solution was added NEt_3 (38 µL, 0.272 mmol, 2 equiv.). The reaction was heated to 50 °C in the microwave for 1 hour. When TLC indicated reaction completion, the reaction was concentrated, and purified via column chromatography using 60:40 EtOAc-Hex. Obtained 42 mg clear film (80% yield).

TLC system: 1:1 EtOAc-Hex (*From left to right: sm = starting material, rxn = reaction, co = cospot*)

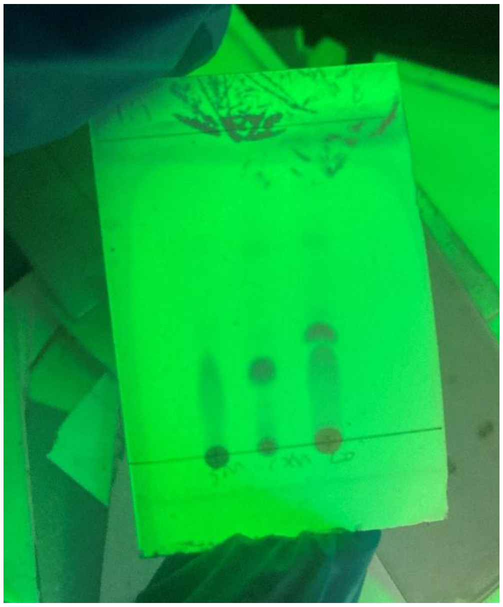

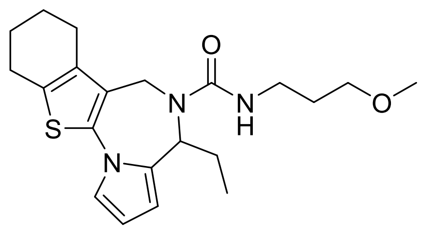

I1 (aka 1564) 7-ethyl-N-(3-methoxypropyl)-17-thia-2,8-diazatetracyclo[8.7.0.0^{2,6}.0^{11,16}]heptadeca-1(10),3,5,11(16)-tetraene-8-carboxamide

^{1}H NMR (400 MHz, $CDCl_3$) δ 0.79-0.83 (t, 3H, J = 7.48 Hz), 1.61-1.68 (m, 3H), 1.74-1.88 (m, 6H), 2.36 (m, 2H), 2.54-2.64 (m, 2H), 3.20 (m, 2H), 3.25 (s, 3H), 3.30-3.33 (td, 2H, 6.08 Hz, 2 Hz), 4.36-4.40 (d, 1H, 16.36 Hz), 4.50-4.54 (t, 1H, 7.52 Hz), 4.66-4.71 (d, 1H, 17.64 Hz), 5.04 (s, 1H), 6.01-6.03 (m, 1H), 6.05-6.07 (m, 1H), 6.83-6.85 (dd, 1H, 2.88 Hz, 1.84 Hz)

^{13}C NMR (125 MHz, $CDCl_3$) δ 11.41, 22.33, 23.15, 24.37, 24.50, 25.20, 29.40, 39.63, 56.14, 58.82, 71.94, 108.79, 109.05, 121.65, 123.78, 128.00, 132.96, 133.20, 134.38, 157.93

Purity (LC-UV trace): 98%

APCI-MS $[M+1]^+$: 388.0

Procedure To vial was added 5-ethoxypentanoic acid (270 mg, 1.84 mmol, 1 equiv.) and 7-(propan-2-yl)-17-thia-2,8-diazatetracyclo[8.7.0.0^{2,6}.0^{11,16}]heptadeca-1(10),3,5,11(16)-tetraene (632 mg, 2.2 mmol, 1.2 equiv) and THF (20 mL). Then added DIPEA (0.96 mL, 5.54 mmol, 3 equiv.) and HATU (942 mg, 2.4 mmol, 1.3 equiv.) and stirred the reaction overnight at room temperature. While TLC indicated consumption of amine starting material, LCMS analysis of reaction mixture showed presence of carboxylic acid starting material. Therefore, added another 0.5 equiv. of amine starting material, 0.5 equiv. of HATU, and another 1 equiv. of DIPEA and stirred overnight at room temperature. While LCMS still indicated presence of carboxylic acid starting material, the reaction was quenched. Concentrated reaction and diluted with EtOAc and washed the organic layer with H_2O and then with saturated NH_4Cl (aq.). Dried the organic layer, concentrated, and purified the crude via column chromatography with a gradient of 2% MeOH-98% DCM to 4% MeOH-96% DCM. Obtained ~1 g red viscous liquid (>100% yield). Repurification by column chromatography using gradient of 20% EtOAc-80% hexanes to 35% EtOAc-65% hexanes afforded 518 mg of a yellow oil (68% yield).

TLC system: 1:1 EtOAc-Hex (*From left to right: sm = amine starting material, rxn = reaction, co = cospot*)

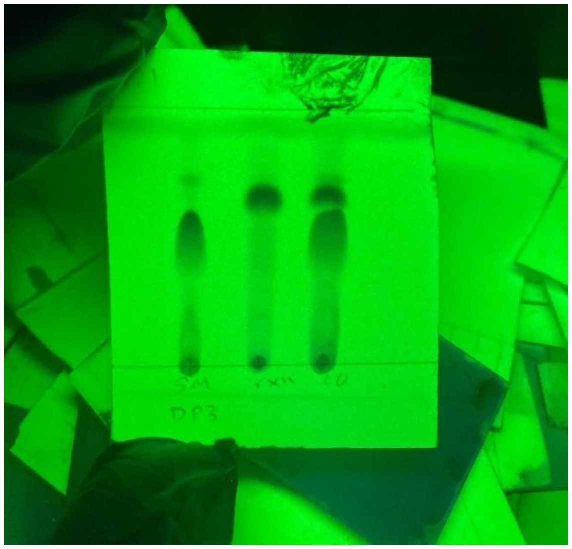

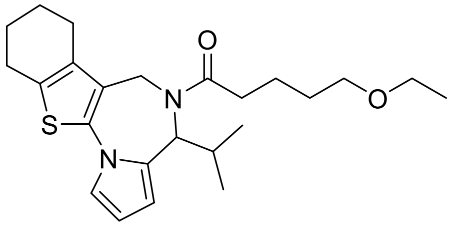

I2 5-ethoxy-1-[7-(propan-2-yl)-17-thia-2,8-diazatetracyclo[8.7.0.0^{2,6}.0^{11,16}]heptadeca-1(10),3,5,11(16)-tetraen-8-yl]pentan-1-one

Mixture of rotamers:

^{1}H NMR (400 MHz, $CDCl_3$) δ 0.73-0.74 (d, 5H, 6.6 Hz), 0.84-0.86 (d, 3H, 6.56 Hz), 0.97-0.99 (d, 4H, 6.4 Hz), 1.14-1.21 (m, 8H), 1.49, 1.85 (m, 19H), 2.10-2.19 (m, 2H), 2.33-2.52 (m, 8H), 2.58-2.74 (m, 5H), 3.39-3.49 (m, 9H), 3.96-4.00 (d, 1H, 17.76 Hz), 4.40-4.43 (d, 2H, 10.96 Hz), 5.48-5.52 (d, 1H, 17.72 Hz), 5.98-6.00 (m, 1H), 6.08-6.10 (t, 1H, J = 6.4 Hz), 6.13-6.14 (d, 1.5 H, 2.52 Hz), 6.82-6.83 (t, 0.5H, 2.36 Hz), 6.85-6.86 (m, 1H)

^{13}C NMR (125 MHz, $CDCl_3$) δ 15.23, 15.26, 20.05, 20.53, 20.78, 22.04, 22.12, 22.23, 22.28, 23.01, 23.11, 24.37, 24.42, 24.58, 28.04, 29.39, 33.54, 34.10, 40.42, 62.23, 66.08, 66.10, 70.26, 70.32, 108.99, 109.51, 109.91, 120.49, 121.06, 121.40, 127.68, 128.94, 131.71, 132.39, 133.33, 133.48, 173.29

Purity (LC-UV trace): 93%

APCI-MS $[M+1]^+$: 415.2

SPECTRA

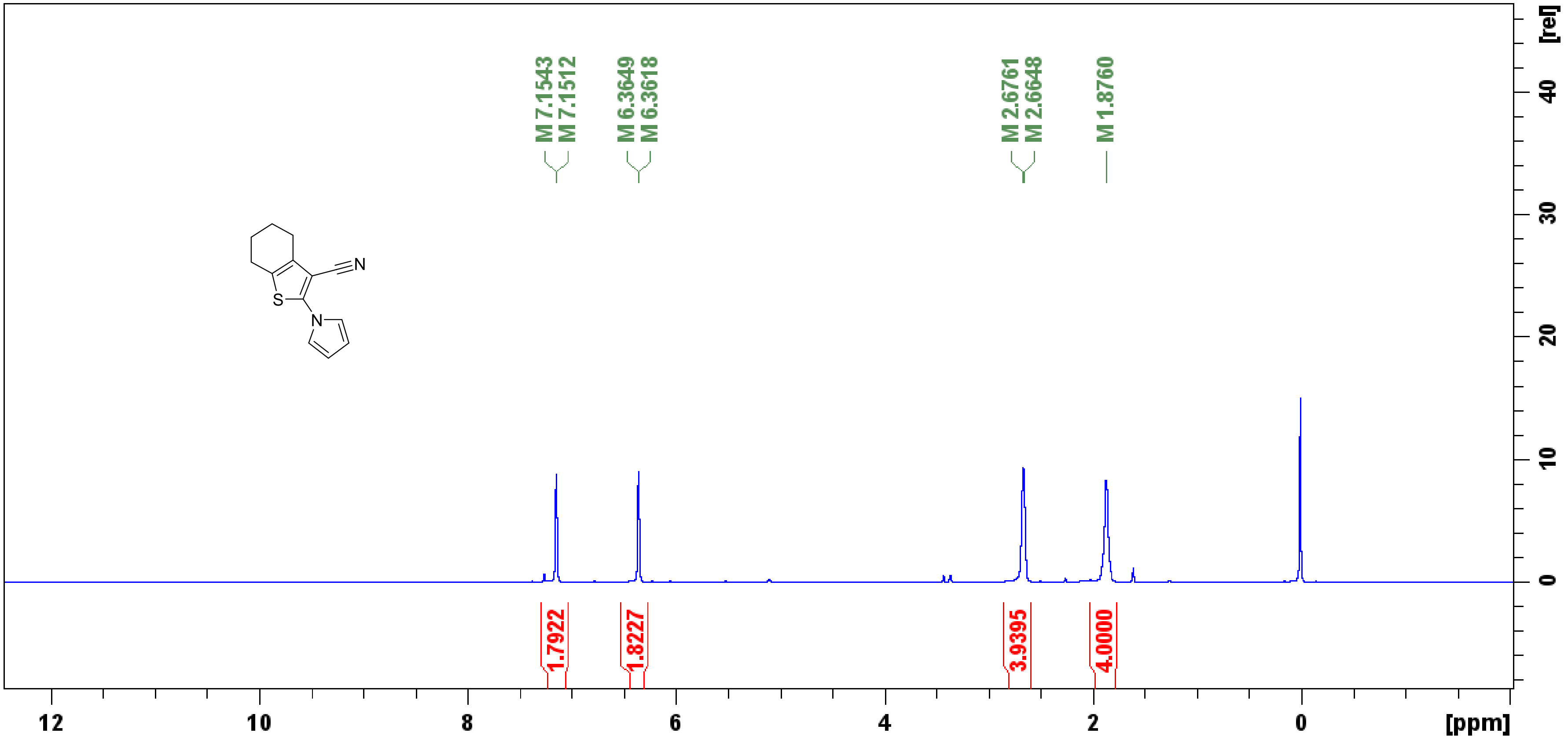

M 7.1543
M 7.1512
M 6.3649
M 6.3618
M 2.6761
M 2.6648
M 1.8760
1.7922
1.8227
3.9395
4.0000
[rel]
40
30
20
10
0
12
10
8
6
4
2
0
[ppm]

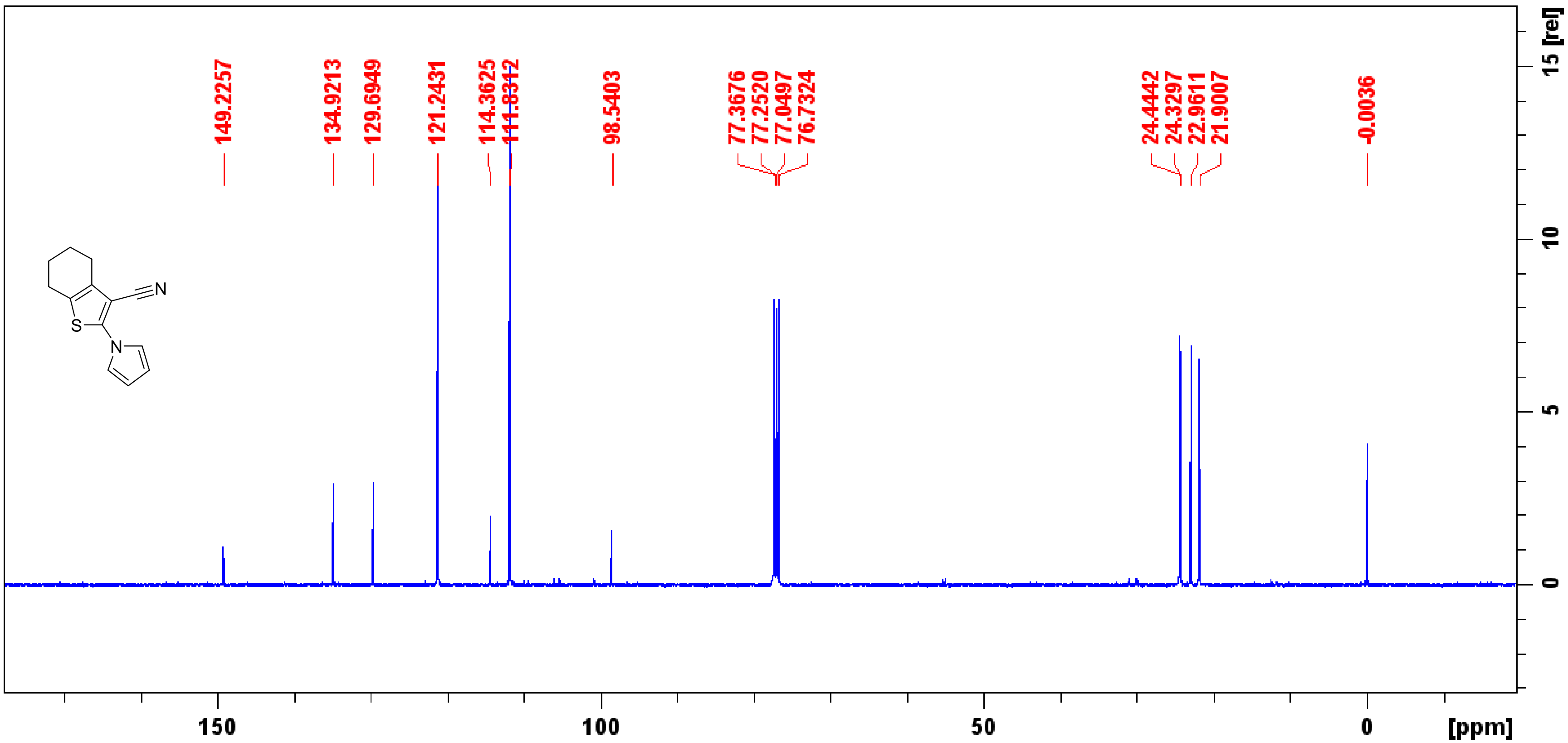

149.2257
134.9213
129.6949
121.2431
114.3625
111.8312
98.5403
77.3676
77.2520
77.0497
76.7324
24.4442
24.3297
22.9611
21.9007
-0.0036
S
N
N
150
100
50
0
[ppm]
15 [rel]
10
5
0

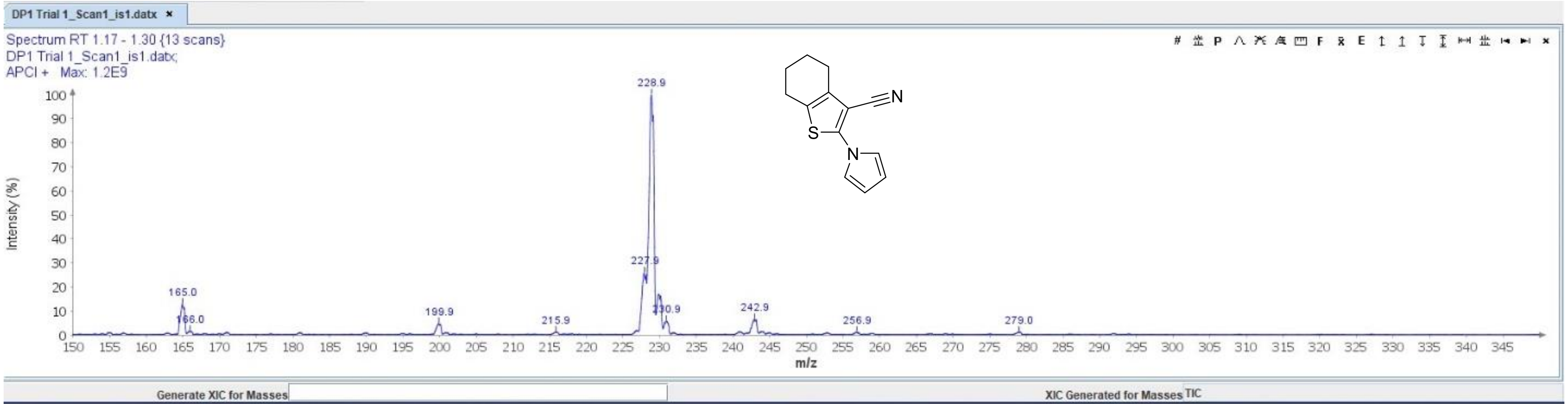
DP1 Trial 1_Scan1_is1.datx
Spectrum RT 1.17 - 1.30 {13 scans}
DP1 Trial 1_Scan1_is1.datx;
APCI + Max: 1.2E9
Intensity (%)
m/z
228.9
227.9
165.0
166.0
199.9
215.9
230.9
242.9
256.9
279.0
S
N
N
Generate XIC for Masses
XIC Generated for Masses TIC

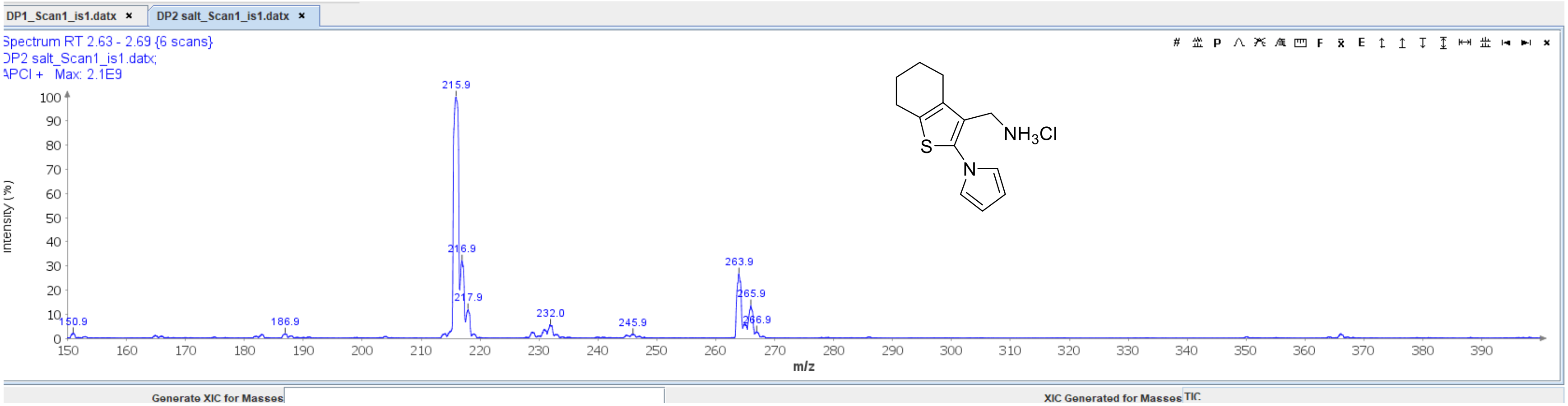
DP1_Scan1_is1.datx
DP2 salt_Scan1_is1.datx
Spectrum RT 2.63 - 2.69 {6 scans}
DP2 salt_Scan1_is1.datx;
APCI + Max: 2.1E9
Intensity (%)
m/z
215.9
216.9
217.9
150.9
186.9
232.0
245.9
263.9
265.9
266.9
S
N
NH_3Cl
Generate XIC for Masses
XIC Generated for Masses TIC

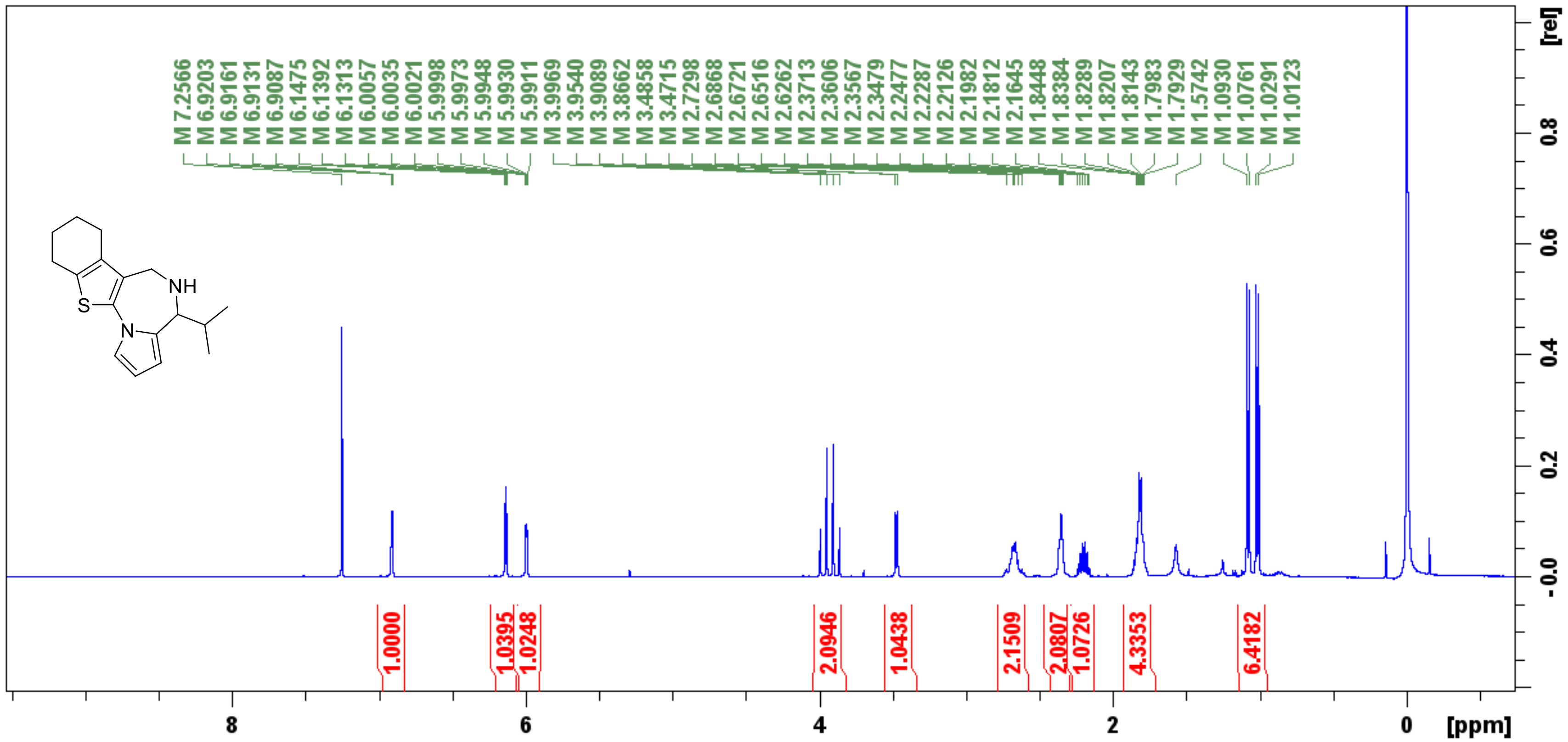

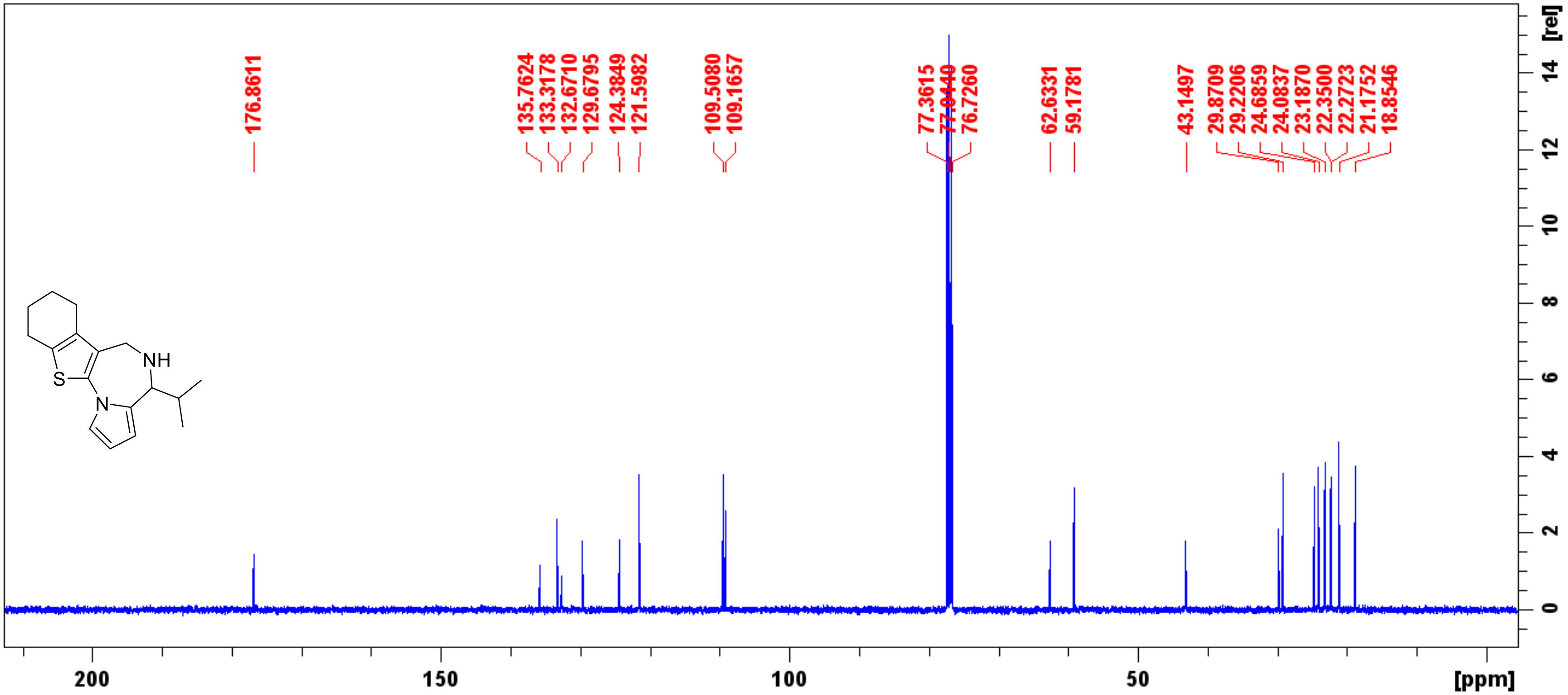
176.8611
135.7624
133.3178
132.6710
129.6795
124.3849
121.5982
109.5080
109.1657
77.3615
77.0440
76.7260
62.6331
59.1781
43.1497
29.8709
29.2206
24.6859
24.0837
23.1870
22.3500
22.2723
21.1752
18.8546
[rel]
[ppm]
S
NH
N

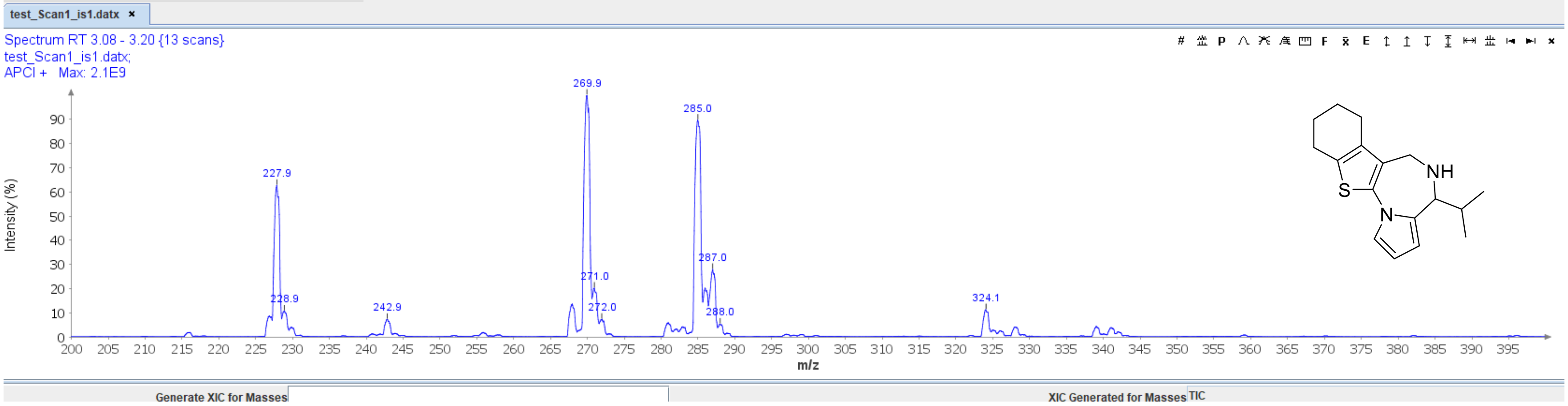
test_Scan1_is1.datx
Spectrum RT 3.08 - 3.20 {13 scans}
test_Scan1_is1.datx;
APCI + Max: 2.1E9
Intensity (%)
m/z
227.9
228.9
242.9
269.9
271.0
272.0
285.0
287.0
288.0
324.1
Generate XIC for Masses
XIC Generated for Masses TIC
S
NH
N

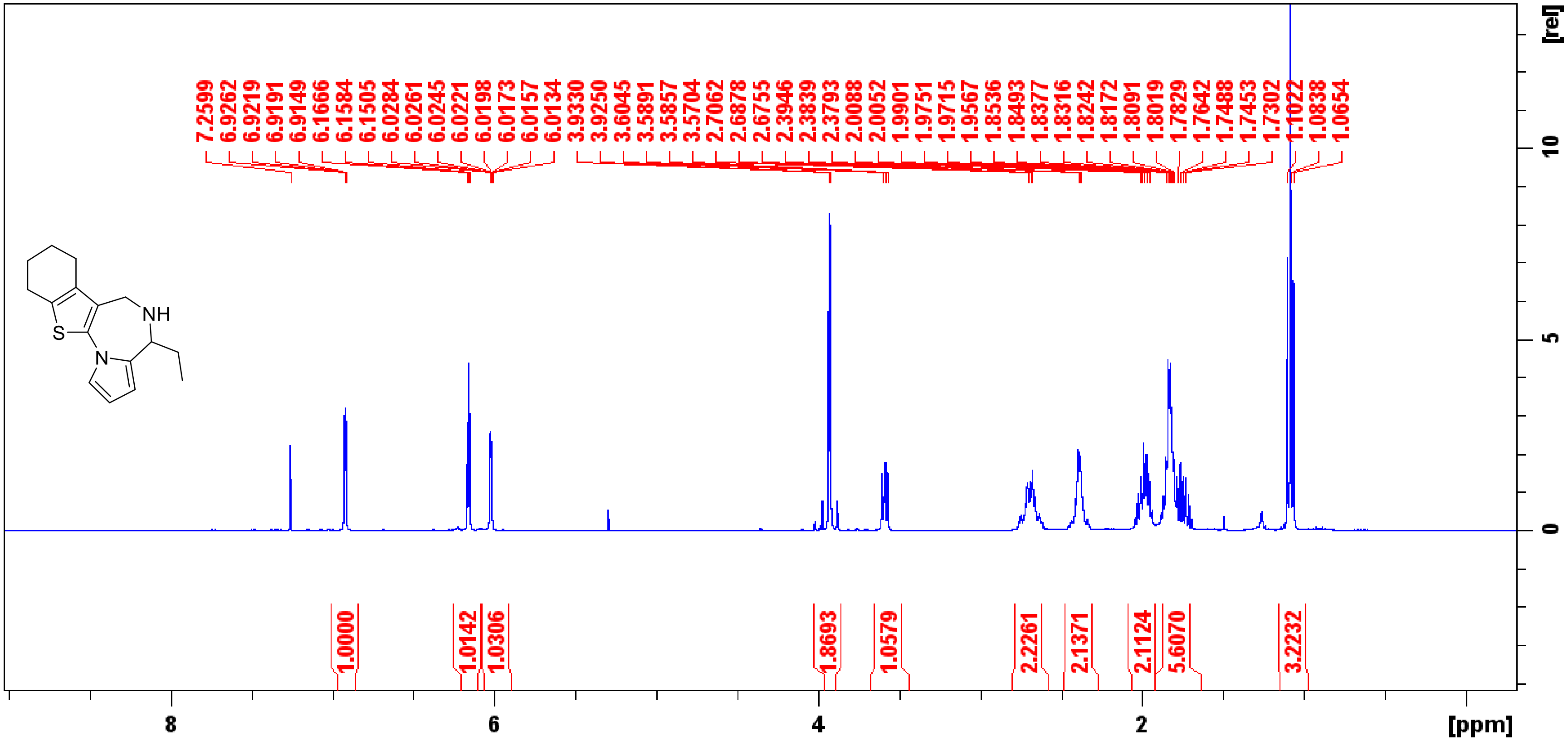
7.2599
6.9262
6.9219
6.9191
6.9149
6.1666
6.1584
6.1505
6.0284
6.0261
6.0245
6.0221
6.0198
6.0173
6.0157
6.0134
3.9330
3.9250
3.6045
3.5891
3.5857
3.5704
2.7062
2.6878
2.6755
2.3946
2.3839
2.3793
2.0088
2.0052
1.9901
1.9751
1.9715
1.9567
1.8536
1.8493
1.8377
1.8316
1.8242
1.8172
1.8091
1.8019
1.7829
1.7642
1.7488
1.7453
1.7302
1.1022
1.0838
1.0654
NH
S
N
1.0000
1.0142
1.0306
1.8693
1.0579
2.2261
2.1371
2.1124
5.6070
3.2232
8
6
4
2
[ppm]
[rel]
10
5
0

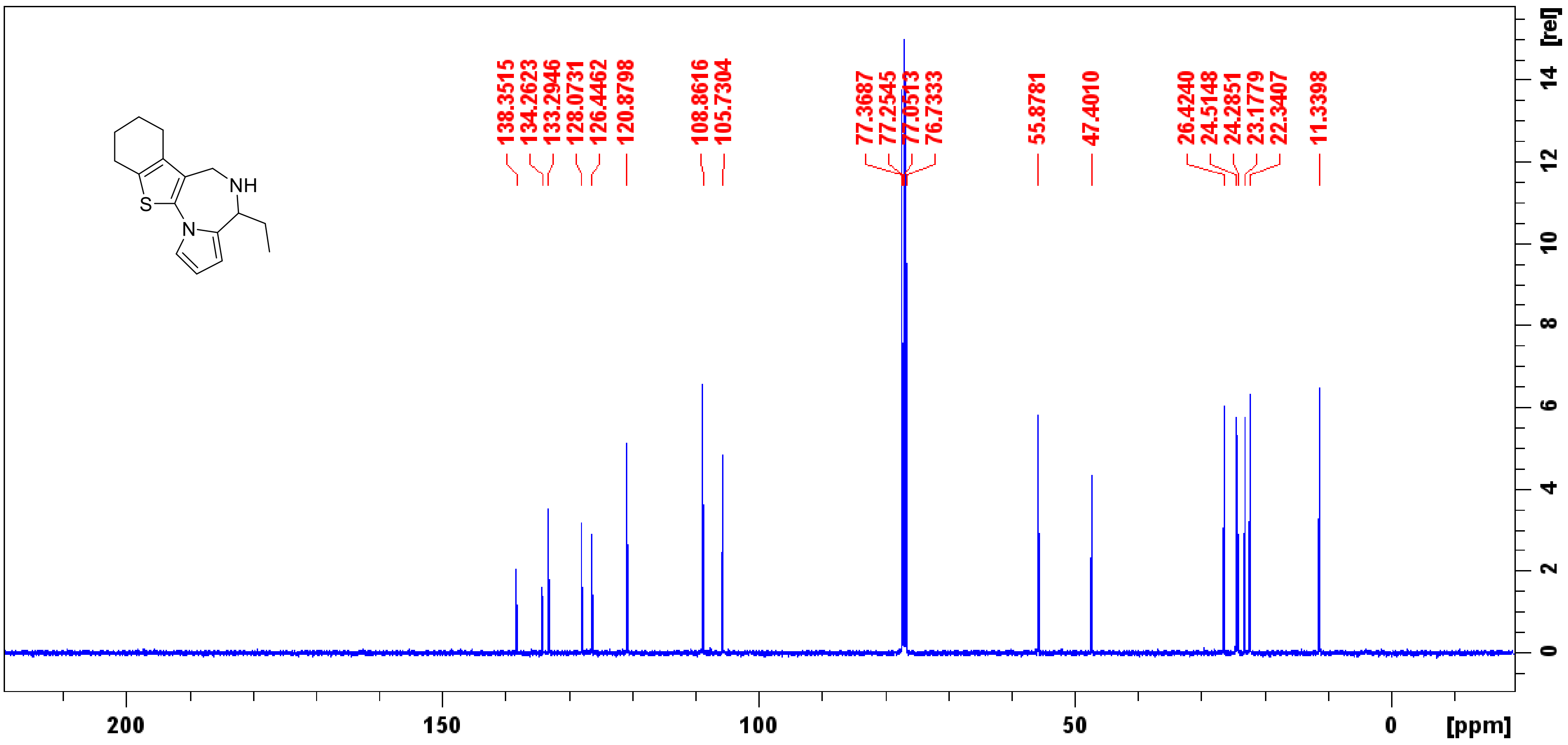

138.3515
134.2623
133.2946
128.0731
126.4462
120.8798
108.8616
105.7304
77.3687
77.2545
77.0513
76.7333
55.8781
47.4010
26.4240
24.5148
24.2851
23.1779
22.3407
11.3398
[rel]
[ppm]
NH
S
N

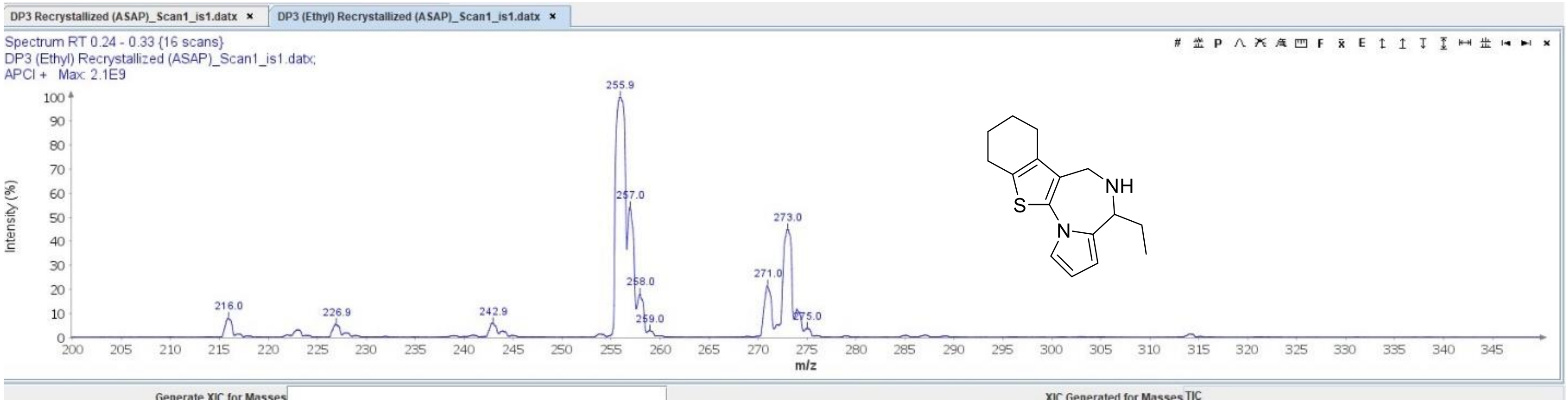

DP3 Recrystallized (ASAP)_Scan1_is1.datx
DP3 (Ethyl) Recrystallized (ASAP)_Scan1_is1.datx
Spectrum RT 0.24 - 0.33 (16 scans)
DP3 (Ethyl) Recrystallized (ASAP)_Scan1_is1.datx;
APCI + Max: 2.1E9
Intensity (%)
m/z
255.9
257.0
258.0
259.0
216.0
226.9
242.9
271.0
273.0
275.0
NH
S
N

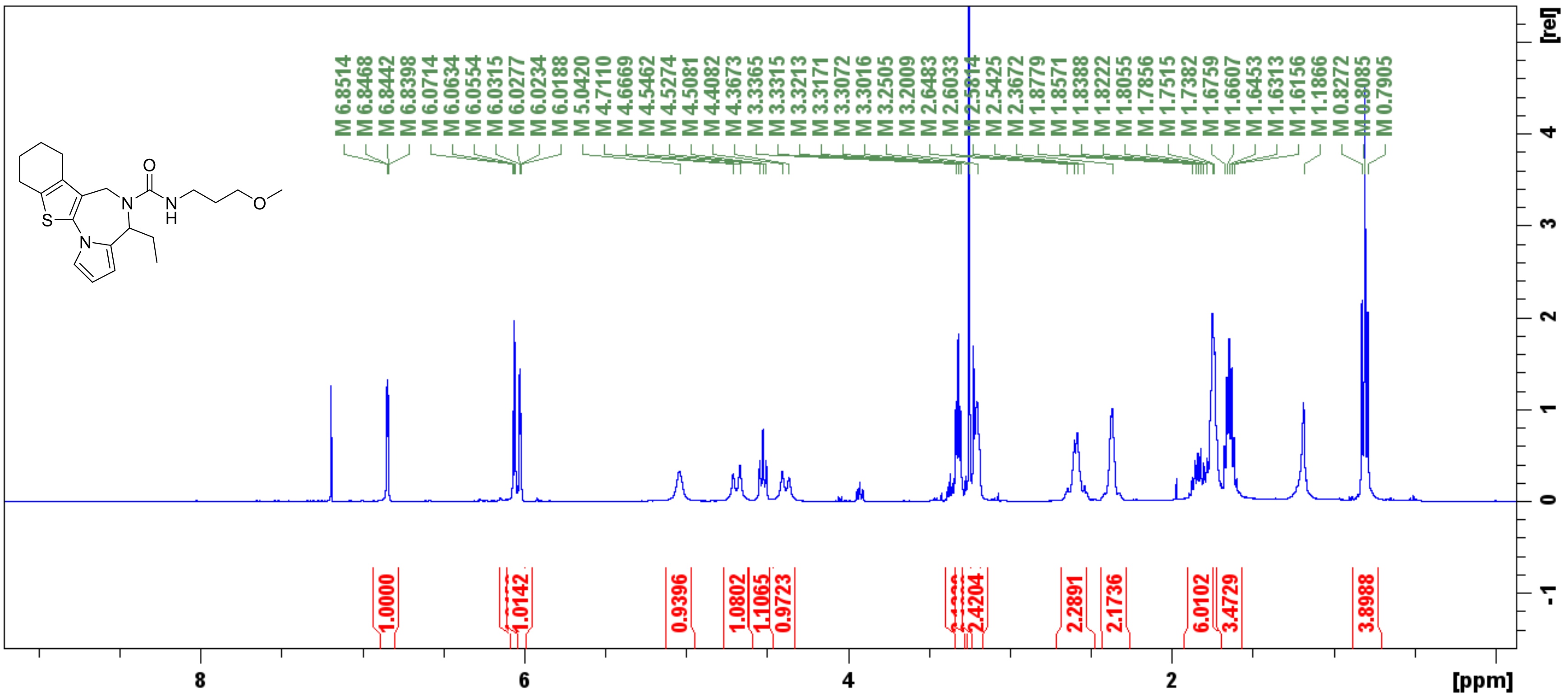
M 6.8514
M 6.8468
M 6.8442
M 6.8398
M 6.0714
M 6.0634
M 6.0554
M 6.0315
M 6.0277
M 6.0234
M 6.0188
M 5.0420
M 4.7110
M 4.6669
M 4.5462
M 4.5274
M 4.5081
M 4.4082
M 4.3673
M 3.3365
M 3.3315
M 3.3213
M 3.3171
M 3.3072
M 3.3016
M 3.2505
M 3.2009
M 2.6483
M 2.6033
M 2.5425
M 2.3672
M 1.8779
M 1.8571
M 1.8388
M 1.8222
M 1.8055
M 1.7856
M 1.7515
M 1.7382
M 1.6759
M 1.6607
M 1.6453
M 1.6313
M 1.6156
M 1.1866
M 0.8272
M 0.7905
1.0000
1.0142
0.9396
1.0802
1.1065
0.9723
2.4204
2.2891
2.1736
6.0102
3.4729
3.8988
[rel]
[ppm]

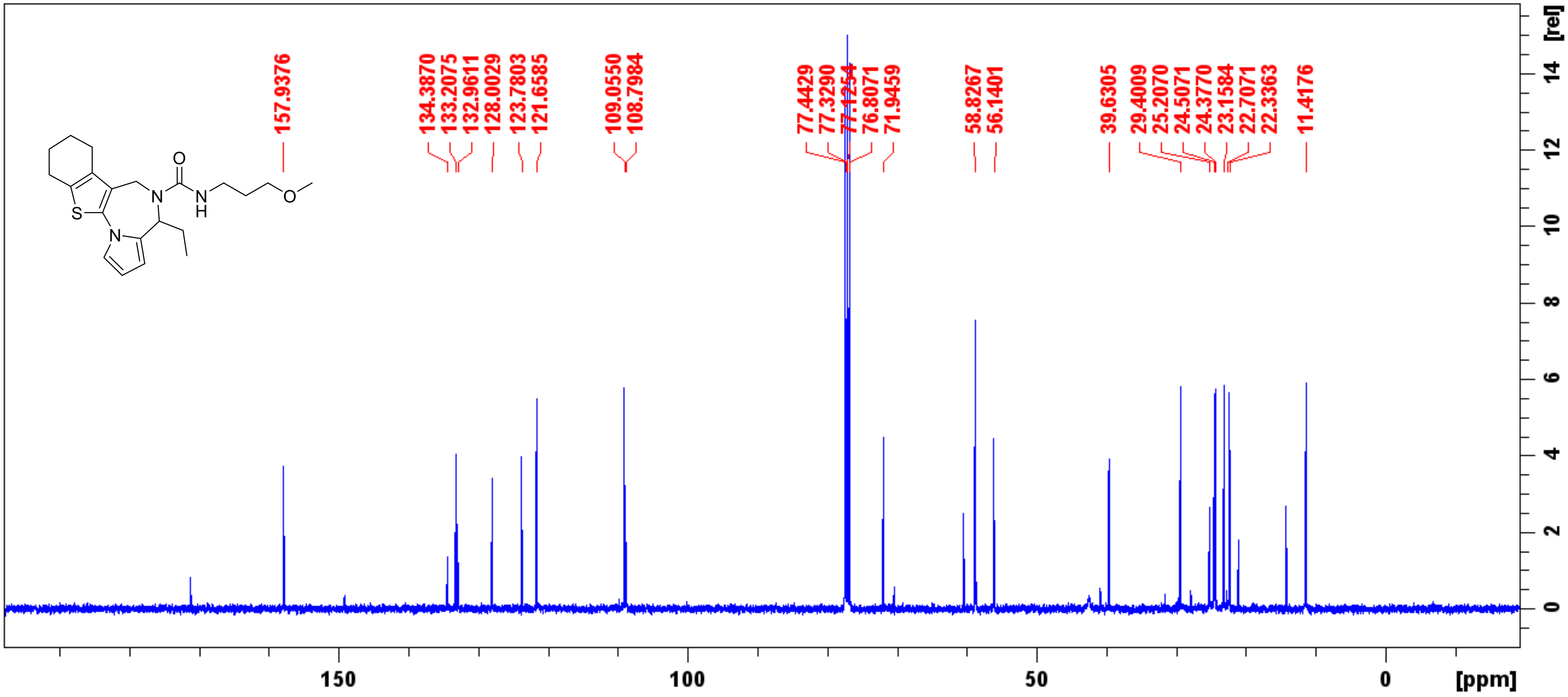
157.9376
134.3870
133.2075
132.9611
128.0029
123.7803
121.6585
109.0550
108.7984
77.4429
77.3290
77.1254
76.8071
71.9459
58.8267
56.1401
39.6305
29.4009
25.2070
24.5071
24.3770
23.1584
22.7071
22.3363
11.4176
[rel]
[ppm]
O
N
N
H
O
S
N

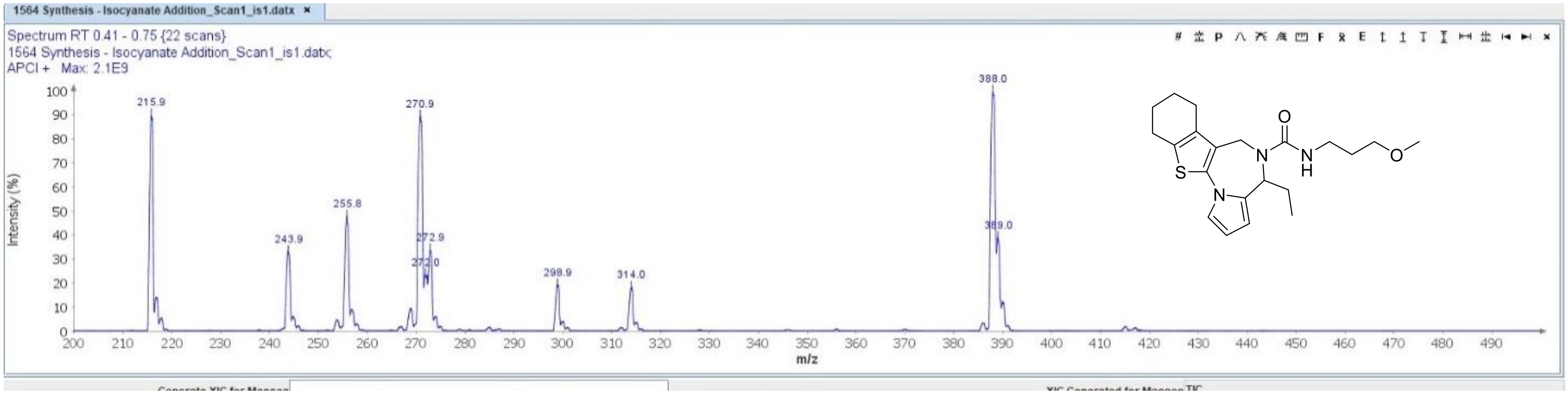
1564 Synthesis - Isocyanate Addition_Scan1_is1.datx
Spectrum RT 0.41 - 0.75 (22 scans)
1564 Synthesis - Isocyanate Addition_Scan1_is1.datx
APCI + Max: 2.1E9
Intensity (%)
m/z
215.9
243.9
255.8
270.9
272.9
298.9
314.0
388.0
389.0

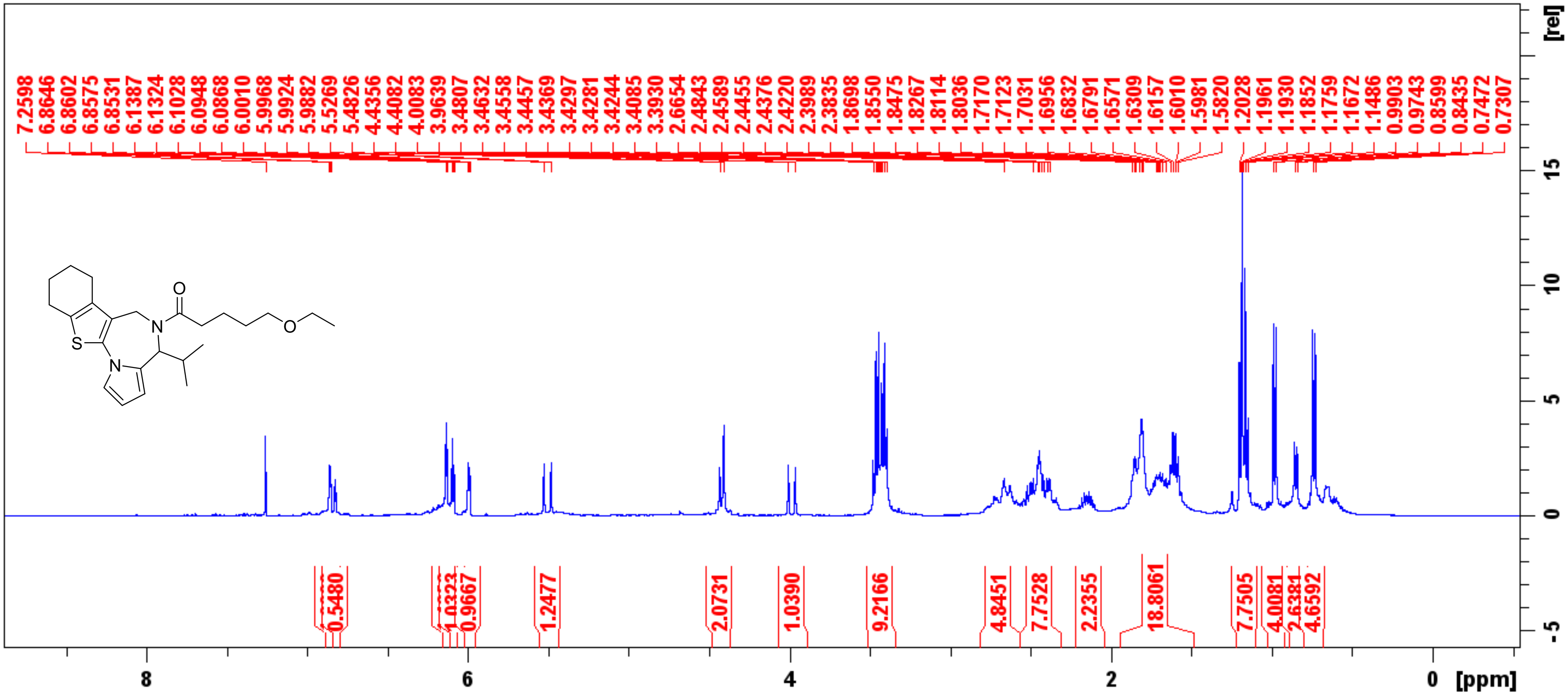
7.2598
6.8646
6.8602
6.8575
6.8531
6.1387
6.1324
6.1028
6.0948
6.0868
6.0010
5.9968
5.9924
5.9882
5.5269
5.4826
4.4356
4.4082
4.0083
3.9639
3.4807
3.4632
3.4558
3.4457
3.4369
3.4297
3.4281
3.4244
3.4085
3.3930
2.6654
2.4843
2.4589
2.4455
2.4376
2.4220
2.3989
2.3835
1.8698
1.8550
1.8475
1.8267
1.8114
1.8036
1.7170
1.7123
1.7031
1.6956
1.6832
1.6791
1.6571
1.6309
1.6157
1.6010
1.5981
1.5820
1.2028
1.1961
1.1930
1.1852
1.1759
1.1672
1.1486
0.9903
0.9743
0.8599
0.8435
0.7472
0.7307
[rel]
0.5480
1.0323
0.9667
1.2477
2.0731
1.0390
9.2166
4.8451
7.7528
2.2355
18.8061
7.7505
4.0081
2.6381
4.6592
8
6
4
2
0
[ppm]
15
10
5
0
-5
O
N
O
S
N

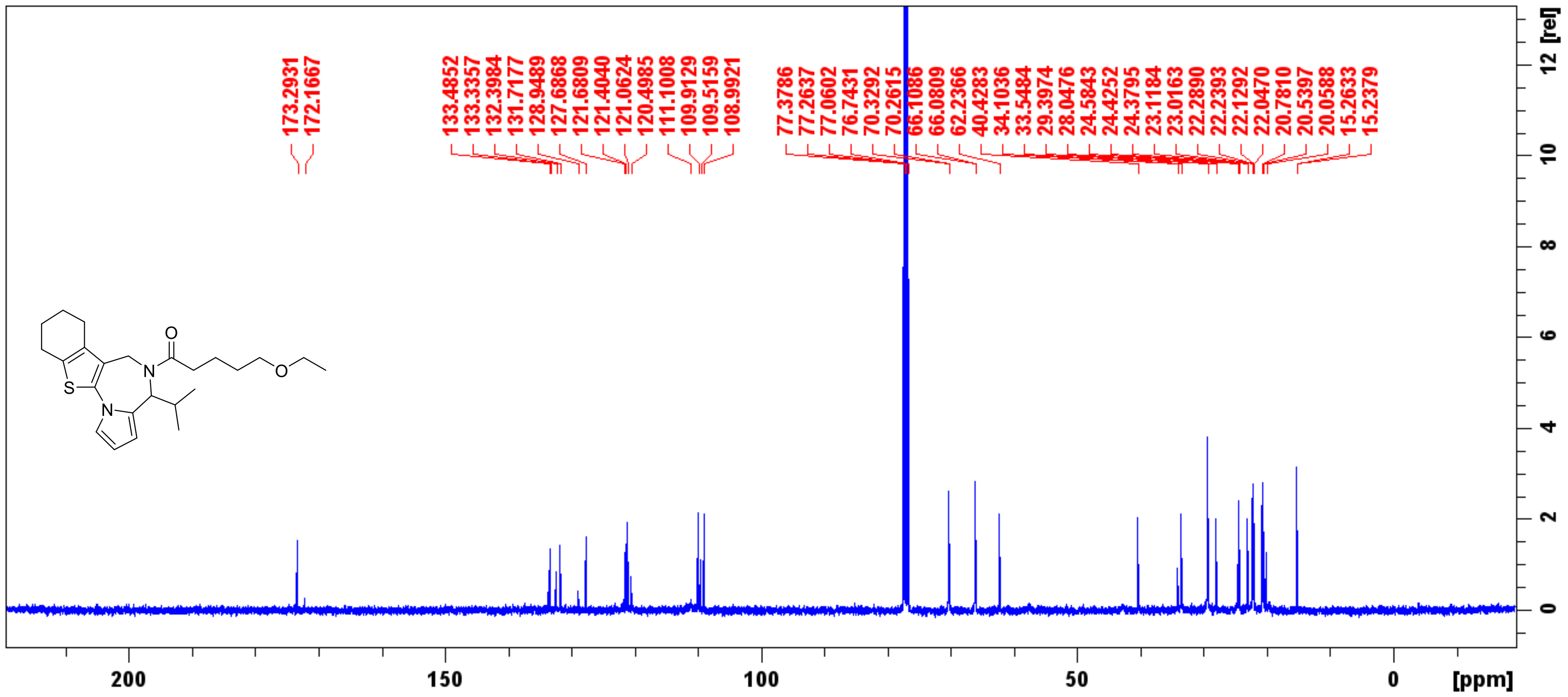
173.2931
172.1667
133.4852
133.3357
132.3984
131.7177
128.9489
127.6868
121.6809
121.4040
121.0624
120.4985
111.1008
109.9129
109.5159
108.9921
77.3786
77.2637
77.0602
76.7431
70.3292
70.2615
66.1086
66.0809
62.2366
40.4283
34.1036
33.5484
29.3974
28.0476
24.5843
24.4252
24.3795
23.1184
23.0163
22.2890
22.2393
22.1292
22.0470
20.7810
20.5397
20.0588
15.2633
15.2379
[rel]
[ppm]

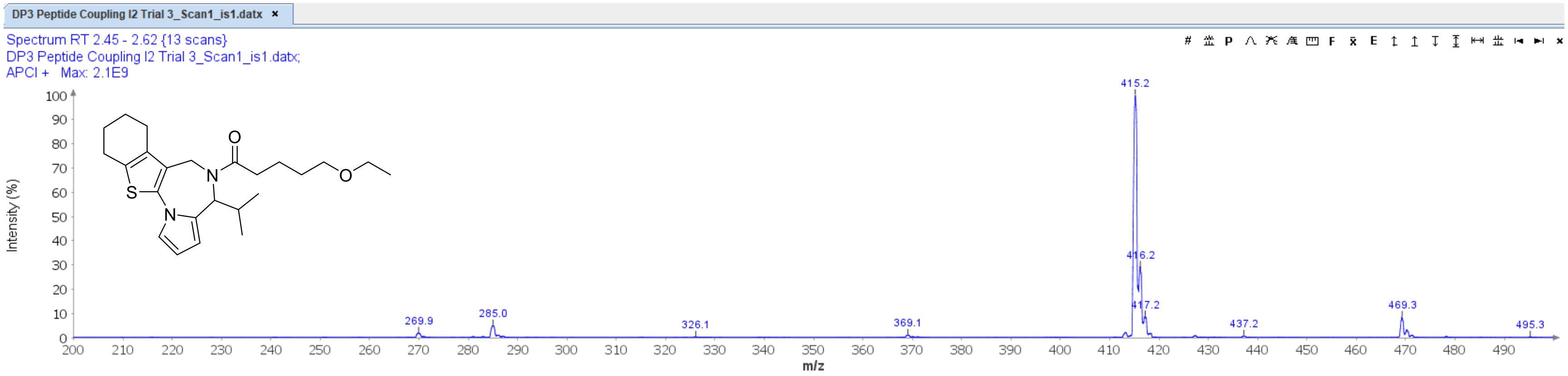
DP3 Peptide Coupling I2 Trial 3_Scan1_is1.datx
Spectrum RT 2.45 - 2.62 {13 scans}
DP3 Peptide Coupling I2 Trial 3_Scan1_is1.datx;
APCI + Max: 2.1E9
Intensity (%)
m/z
415.2
416.2
417.2
269.9
285.0
326.1
369.1
437.2
469.3
495.3

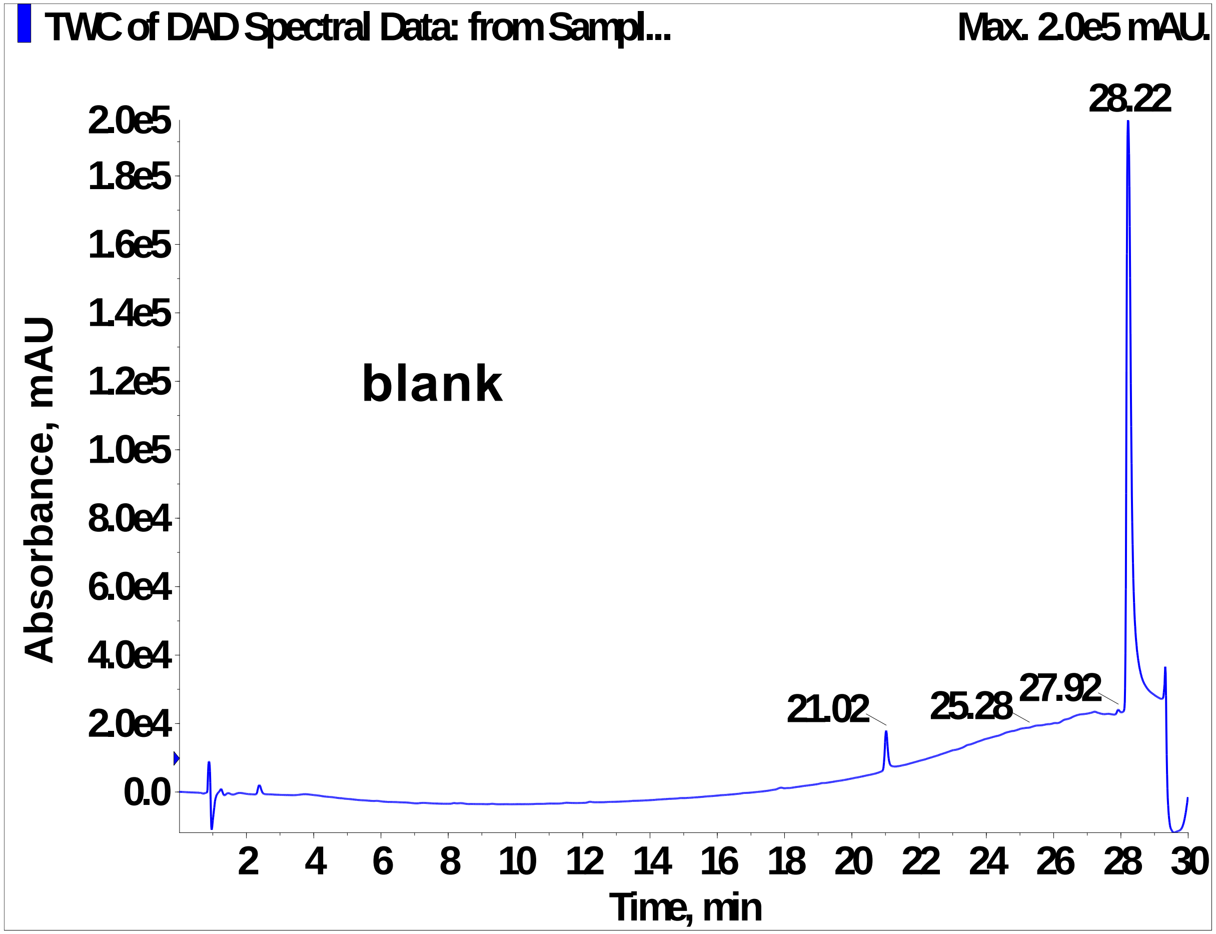
TWC of DAD Spectral Data: from Sampl...
Max. 2.0e5 mAU.
blank
28.22
27.92
25.28
21.02
Absorbance, mAU
2.0e5
1.8e5
1.6e5
1.4e5
1.2e5
1.0e5
8.0e4
6.0e4
4.0e4
2.0e4
0.0
2 4 6 8 10 12 14 16 18 20 22 24 26 28 30
Time, min

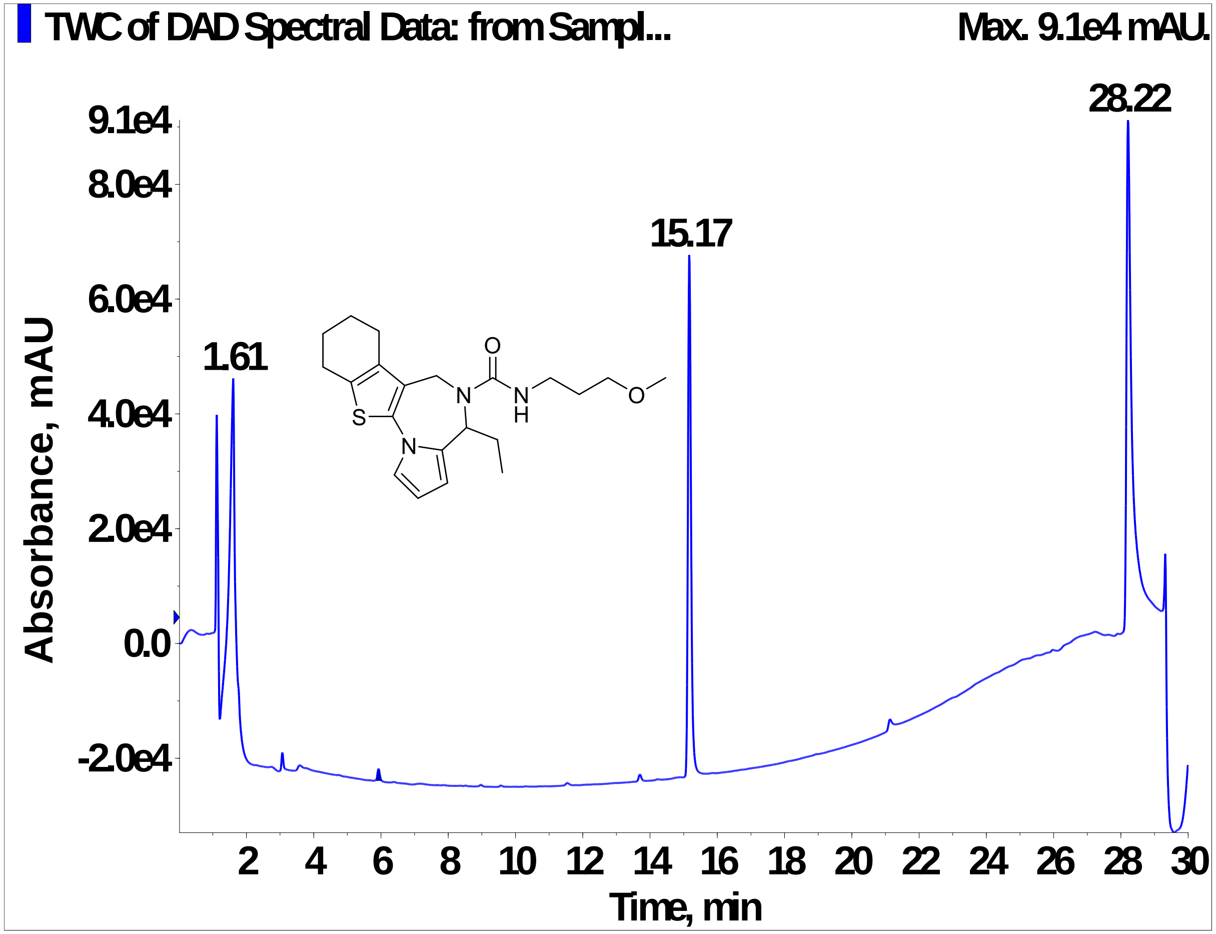

TWC of DAD Spectral Data: from Sampl...
Max. 9.1e4 mAU.
9.1e4
8.0e4
6.0e4
4.0e4
2.0e4
0.0
-2.0e4
Absorbance, mAU
1.61
15.17
28.22
O
N
N
H
O
S
N
2
4
6
8
10
12
14
16
18
20
22
24
26
28
30
Time, min

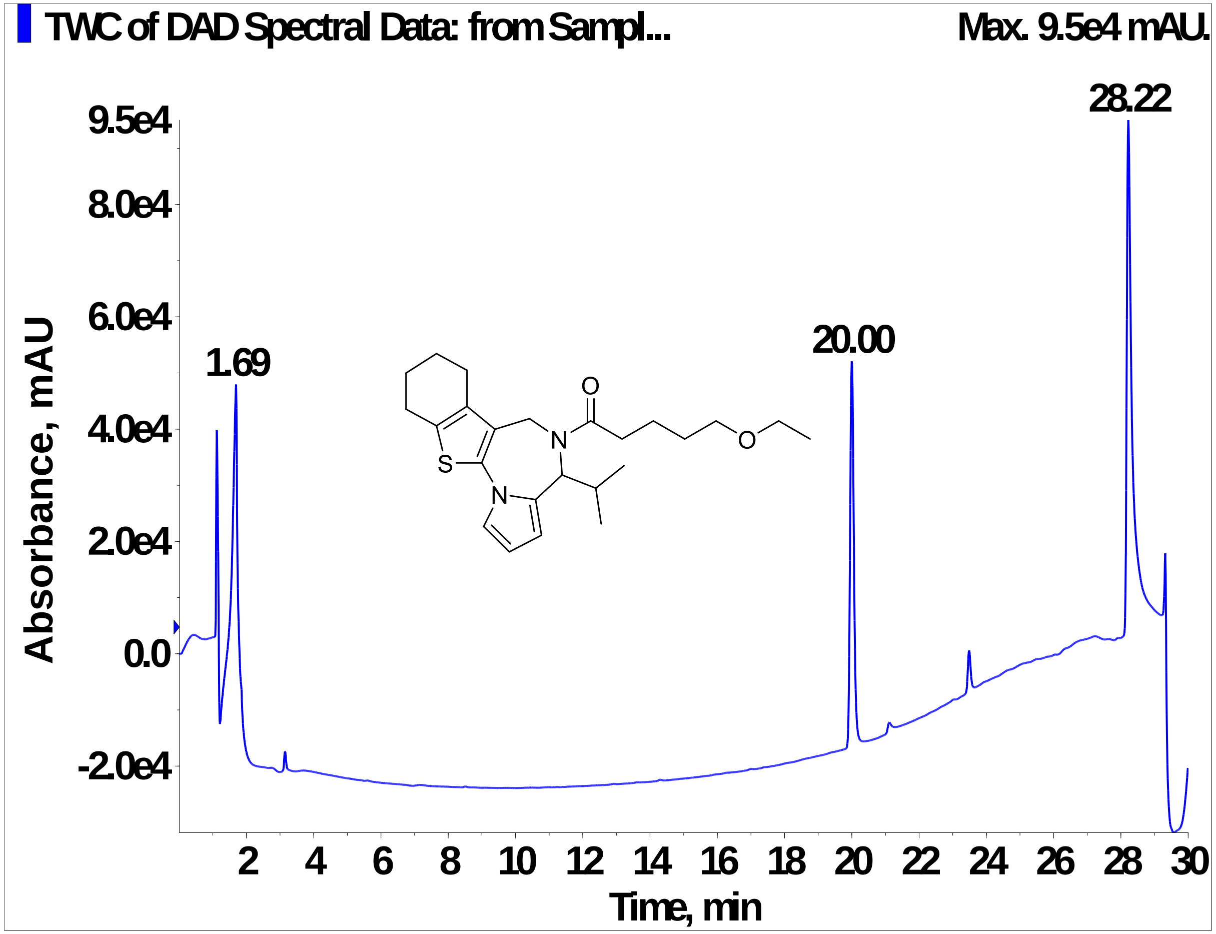

TWC of DAD Spectral Data: from Sampl...
Max. 9.5e4 mAU.
Absorbance, mAU
9.5e4
8.0e4
6.0e4
4.0e4
2.0e4
0.0
-2.0e4
1.69
20.00
28.22
2
4
6
8
10
12
14
16
18
20
22
24
26
28
30
Time, min
O
N
O
S
N

Expression and purification of recombinant importin-α (ImpA)

Recombinant ImpA (residues 70-529) with a C-terminal $(His)_6$-tag (GenScript) was expressed in E. coli BL21(DE3)pLys cells. The ImpA was incubated in LB media containing kanamycin at 37°C and induced by 1.0 mM isopropyl β-D-thiogalactopyranoside (IPTG) at $O.D._{600}$ at 16 °C as described [2]. Cells were harvested and lysed by sonication for 20 minutes in lysis buffer containing 20 mM Tris pH 8.0, 150 mM NaCl, 10 % glycerol, and 25 mM imidazole. The lysate was centrifuged at 19000g for 30 min at 4°C, then the supernatant was loaded onto a Histrap HP column (GE Healthcare). ImpA was eluted with a linear gradient of 25−500 mM imidazole containing 20 mM Tris pH 8.0, 150 mM NaCl, and 10% glycerol buffer solution. The pooled peaks were loaded onto a Mono Q column (GE Healthcare), washed with 10 mM Tris-HCl pH 8.0 buffer, and eluted with a linear gradient of 0−0.5 M KCl in 10 mM Tris-HCl pH 8.0 buffer. The ImpA was centrifuged using a 50-kDa Amicon Centrifugal filter for protein concentration and buffer exchange in the final buffer (50 mM Tris pH 8.0, 150 mM NaCl, 1% glycerol). The isolation of ImpA was determined as >95% pure by SDS-PAGE.

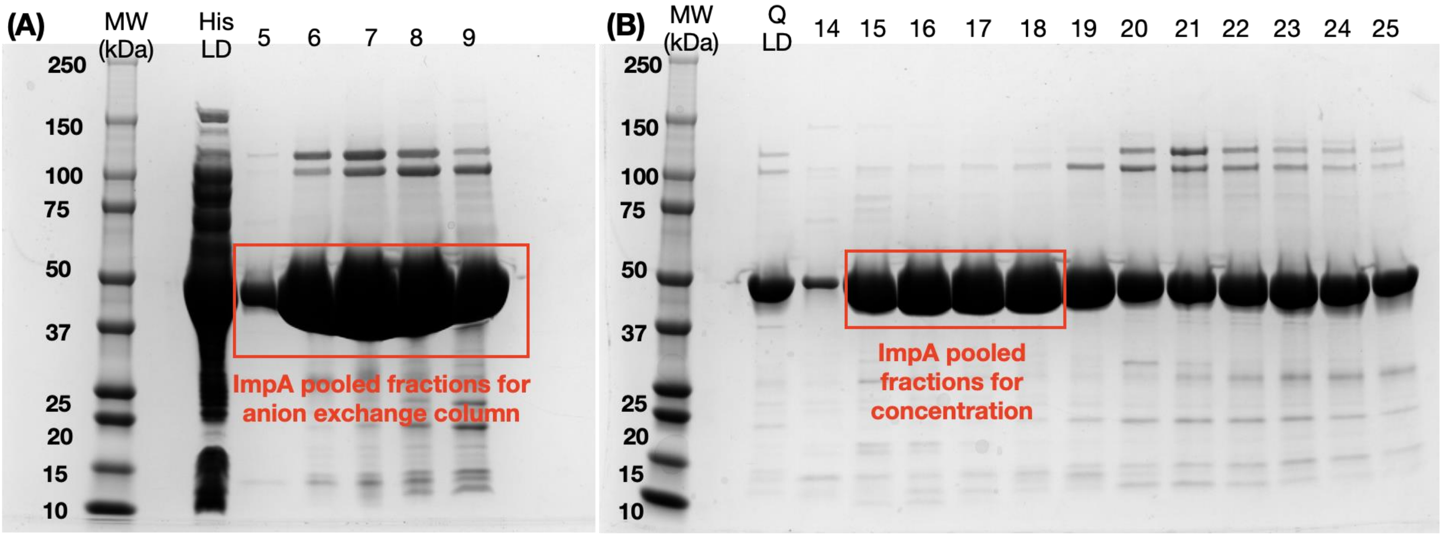

Figure 1. ImpA SDS-PAGE gel. The purification of ImpA from HisTrap (A) and Mono Q anion exchange column was evaluated by an SDS-PAGE gel.

ImpA sequence (residues 70-529)

MNQGTVNWSVDDIVKGINSSNVENQLQATQAARKLLSREKQPPIDNIIRAGLIPKFVSFLGRTDCSPIQFESAWALTNIASGTS
EQTKAVVDGGAIPAFISLLASPHAHISEQAVWALGNIAGDGSVFRDLVIKYGAVDPLLALLAVPDMSSLACGYLRNLTWTLS
NLCRNKNPAPPIDAVEQILPTLVRLLHHDDPEVLADTCWAISYLTDGPNERIGMVVKTGVVPQLVKLLGASELPIVTPALRAI

GNIVTGTDEQTQVVIDAGALAVFPSLLTNPKTNIQKEATWTMSNITAGRQDQIQQVVNHGLVPFLVSVLSKADFKTQKEAV
WAVTNYTSGGTVEQIVYLVHCGIIEPLMNLLTAKDTKIILVILDAISNIFQAAEKLGETEKLSIMIEECGGLDKIEALQNHENES
VYKASLSLIEKYFSVEEEEDQNVVPETTSEGYTFQVQDGAPGTFNF

FLAG-tagged NLS (12-mer) sequence used in AlphaScreen
DYKDDDDK**EGPSAKKPKKEA**

AlphaScreen Inhibition Assay

The inhibitory assay of ImpA in the presence of compounds I1 and I2 was determined by AlphaScreen assay in a 384-well Perkin-Elmer white opaque-bottom plate. 1 μL of a 1:10 dilution of the acceptor beads, 1 μL of 2.5% bovine serum albumin (BSA), and 1 μL of a 1:10 dilution of the donor beads was prepared in a dark room. Assays were performed in a total reaction volume of 25 μL containing 5 μL of His-tagged ImpA [2.5 nM], 5 μL of FLAG-tagged NLS [60 nM], 5 μL of I1 or I2 in various concentrations [0-800 μM], and 7 μL of 1xPBS pH 7.2 buffer in the presence of 3 μL of donor and acceptor beads. [3] The plate was incubated at room temperature away from light for 30 minutes and subsequently read on a Tecan Spark 10 M Spectrometer with Alpha Assay protocol. Samples were prepared in 6 replicates and IC_{50} values were calculated by fitting to a four-parameter nonlinear regression curve in GraphPad Prism 10.

References

(1) Byeon, H.-J., Jung, K.-H., Moon, G.-S., Moon, S.-K., and Lee, H.-Y. (2020) A Facile and Efficient Method for the Synthesis of Crystalline Tetrahydro-β-Carbolines via the Pictet-Spengler Reaction in Water. *Sci Rep*, 10, 1057.

(2) Zheng, W, Wang, R, Liu, X, Tian, S., Yao, B., Chen, A., Jin, S., and Li, Y. (2018) Structural insights into the nuclear import of the histone acetyltransferase males-absent-on-the-first by importin α1. Traffic. 19, 19–28.

(3) Wagstaff, K. M. and Jans, D. A. (2006) Intramolecular masking of nuclear localization signals: Analysis of importin binding using a novel AlphaScreen-based method, *Analytical Biochemistry* **348**, 49-56,
